# An investigation of Y chromosome incorporations in 400 species of *Drosophila* and related genera

**DOI:** 10.1371/journal.pgen.1007770

**Published:** 2018-11-02

**Authors:** Eduardo G. Dupim, Gabriel Goldstein, Thyago Vanderlinde, Suzana C. Vaz, Flávia Krsticevic, Aline Bastos, Thadeo Pinhão, Marcos Torres, Jean R. David, Carlos R. Vilela, Antonio Bernardo Carvalho

**Affiliations:** 1 Departamento de Genética, Universidade Federal do Rio de Janeiro, Rio de Janeiro, RJ, Brazil; 2 Departamento de Genética e Biologia Evolutiva, Universidade de São Paulo, São Paulo, SP, Brazil; 3 CIFASIS, CONICET, Rosario, Santa Fe, Argentina; 4 Laboratoire Evolution, Génomes et Spéciation (LEGS), CNRS, France; Stowers Institute for Medical Research, UNITED STATES

## Abstract

Y chromosomes are widely believed to evolve from a normal autosome through a process of massive gene loss (with preservation of some male genes), shaped by sex-antagonistic selection and complemented by occasional gains of male-related genes. The net result of these processes is a male-specialized chromosome. This might be expected to be an irreversible process, but it was found in 2005 that the *Drosophila pseudoobscura* Y chromosome was incorporated into an autosome. Y chromosome incorporations have important consequences: a formerly male-restricted chromosome reverts to autosomal inheritance, and the species may shift from an XY/XX to X0/XX sex-chromosome system. In order to assess the frequency and causes of this phenomenon we searched for Y chromosome incorporations in 400 species from *Drosophila* and related genera. We found one additional large scale event of Y chromosome incorporation, affecting the whole *montium* subgroup (40 species in our sample); overall 13% of the sampled species (52/400) have Y incorporations. While previous data indicated that after the Y incorporation the ancestral Y disappeared as a free chromosome, the much larger data set analyzed here indicates that a copy of the Y survived as a free chromosome both in *montium* and *pseudoobscura* species, and that the current Y of the *pseudoobscura* lineage results from a fusion between this free Y and the neoY. The 400 species sample also showed that the previously suggested causal connection between X-autosome fusions and Y incorporations is, at best, weak: the new case of Y incorporation (*montium*) does not have X-autosome fusion, whereas nine independent cases of X-autosome fusions were not followed by Y incorporations. Y incorporation is an underappreciated mechanism affecting Y chromosome evolution; our results show that at least in *Drosophila* it plays a relevant role and highlight the need of similar studies in other groups.

## Author summary

In contrast to other chromosomes (X and autosomes), which are present in males and females, Y chromosomes spend all time in males. Hence it is not surprising that along evolution they became male specialized, *e*.*g*., containing a disproportionate amount of male-fertility genes. Interestingly it was found in 2005 that in *Drosophila pseudoobscura* the Y chromosome reverted to "male-female existence", being incorporated into an autosome. These "Y chromosome incorporations" have important consequences on sex-chromosome evolution, and allow the study of the evolutionary forces that shaped Y chromosomes as they act backwards. As *D*. *pseudoobscura* was the second *Drosophila* species investigated in this respect, it is likely that other cases exist, and that perhaps it is a common phenomenon. In order to answer this question we studied 400 *Drosophila* species. We found one additional case of Y incorporation, which occurred in the ancestor of *Drosophila montium*, and currently affects a large number of species; overall 13% of the species we sampled (52/400) have Y incorporations. We also found that a previously suggested cause of Y incorporations (X-autosome fusions) is not a general explanation. Our results show that in *Drosophila* Y incorporations play a relevant role and highlight the need of similar studies in other groups.

## Introduction

Sex-chromosomes are believed to originate from an ordinary pair of autosomes, after one of them acquires a strong male (or female) determining gene ("*M*"), becoming a proto-Y (or proto-W; for the sake of simplicity we will refer to both W and Y chromosomes as "Y chromosome"). Natural selection would then favor the accumulation of sex-antagonistic alleles (male-beneficial / female-detrimental) in the vicinity of the *M* locus, and the gradual suppression of recombination between the proto-X and proto-Y. The absence of recombination ultimately leads to massive gene loss in the proto-Y, with preservation of a few genes; a few male-related genes are also usually acquired from the other chromosomes. The proto-Y becomes a "mature" Y chromosome, containing the male sex-determining gene, some other male related genes (*e*.*g*., male-fertility factors), a few relic house-keeping genes (most of them with homologs in the X), and a large amount of repetitive DNA [1–3]. This "canonical route" is best illustrated and empirically supported by mammalian Y chromosomes: the human Y encodes only 27 proteins (among them, the product of the master sex-determining gene *SRY*) and 18 of them are shared with the X, whereas the X has a gene content compatible with its size (~1100 genes; [4,5]). Hence the mammalian Y is largely an impoverished ("degenerated") version of the X chromosome [5,6]. This process seems to have occurred independently in many other groups, such as birds, fishes, and plants [1,7,8]. It must be noted, however, that the most informative data—gene content of the Y—is well known in very few species, even after genome sequencing: as with other repeat-rich regions, Y chromosomes are very hard to assemble, requiring special methods that are time consuming and/or expensive [5,9–16]. When such data became available in *Drosophila* it revealed striking discrepancies with the canonical route (reviewed in ref [17]).

Given its male-restricted condition it is not surprising that the gene content of Y chromosomes usually is heavily male-biased [5,11]. Furthermore, for Y chromosomes that evolved through the canonical model, accumulation of male-beneficial alleles lies at the heart of their origin. Hence cases in which Y chromosomes revert to autosomal inheritance are particularly interesting because (i) they offer the unique opportunity to study the forces that shaped the Y chromosome (lack of recombination; male-restricted status, reduced effective population size) after they disappear or are reversed; (ii) they allow the study of the origin of Y chromosomes (below). That such reversals occur is hinted by the observation of turnover of sex-chromosomes (*e*.*g*., refs. [18–20]). One of the best known cases was discovered in 2005: in the *D*. *pseudoobscura* lineage the ancestral Y chromosome became part of an autosome, and was replaced by a Y chromosome with unknown origin; the formerly Y-linked genes now have autosomal inheritance [21–23]. As a consequence, the current *D*. *pseudoobscura* Y chromosome shares no genes with the ancestral *Drosophila* Y (which is present in *D*. *melanogaster* and most species), despite their similarities (both pair with the X, are heterochromatic, and are essential for male fertility). Following Ellison [24], we use here the term "Y incorporation" (meaning "Y chromosome incorporation into an autosome or the X") to distinguish this phenomenon from the usual Y-autosome fusions, in which the ancestral Y keeps its sex-chromosome and male-restricted state, and the fused autosome become a neoY [25–27]. Y incorporations and replacements have a direct bearing on the origin and evolution of these chromosomes, and are the main subject of the present paper. We aim here to answer two questions: (i) How frequent are Y incorporations, as happened in the *D*. *pseudoobscura* lineage? (ii) It has been suggested that in *D*. *pseudoobscura* lineage the Y incorporation was an adaptive response to a (presumably) previous event of X-autosome fusion, which created X-Y segregation problems [21]. Is this a robust explanation? The sample of 12 *Drosophila* species analyzed before [28,29] is insufficient to answer these questions. Here we report a search for Y incorporations and individual gene movements in 400 species of *Drosophila* and related genera.

## Results

**Fig 1:**
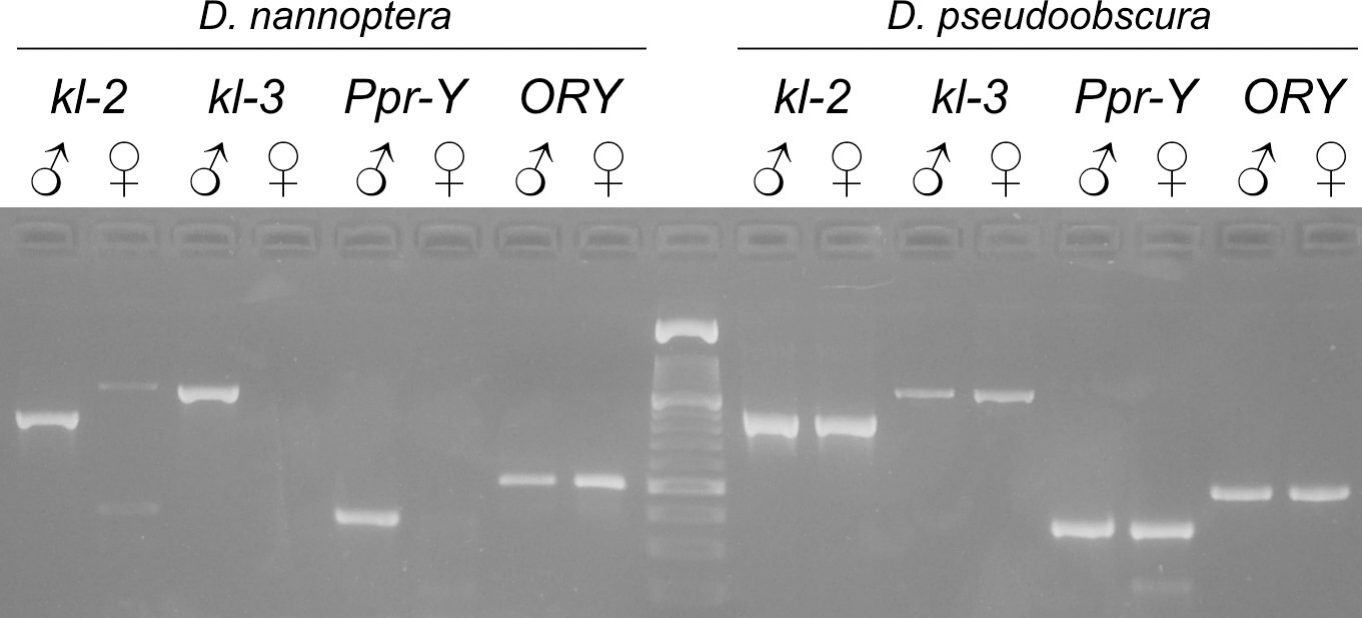
Primary data for detection of Y incorporations and individual gene movements. A) Degenerated PCR of four genes (*kl-2*, *kl-3*, *Ppr-Y* and *ORY*) in males and females of *D*. *nannoptera* and *D*. *pseudoobscura*. These four genes are part of the ancestral Y chromosome of the *Drosophila* genus. Note that in *D*. *nannoptera* the *ORY* gene shifted to amplification on both sexes, indicating an individual gene movement, whereas in *D*. *pseudoobscura* all genes were affected, indicating a Y incorporation event. Analogous experiments were performed for nine genes in 400 species. *kl-2* primers: kl2_KVME_F1 / kl2_QMQE_R1 (860 bp); *kl-3*: kl3_DKMD_F / kl3_EMQD_R (1140 bp); *Ppr-Y*: PprY_FVEH_ns_F1 / PprY_MHGE_R1 (380 bp); *ORY*: ORY_YKNI_F1 / ORY_IEKE_R1 (500 bp).

**Fig 2:**
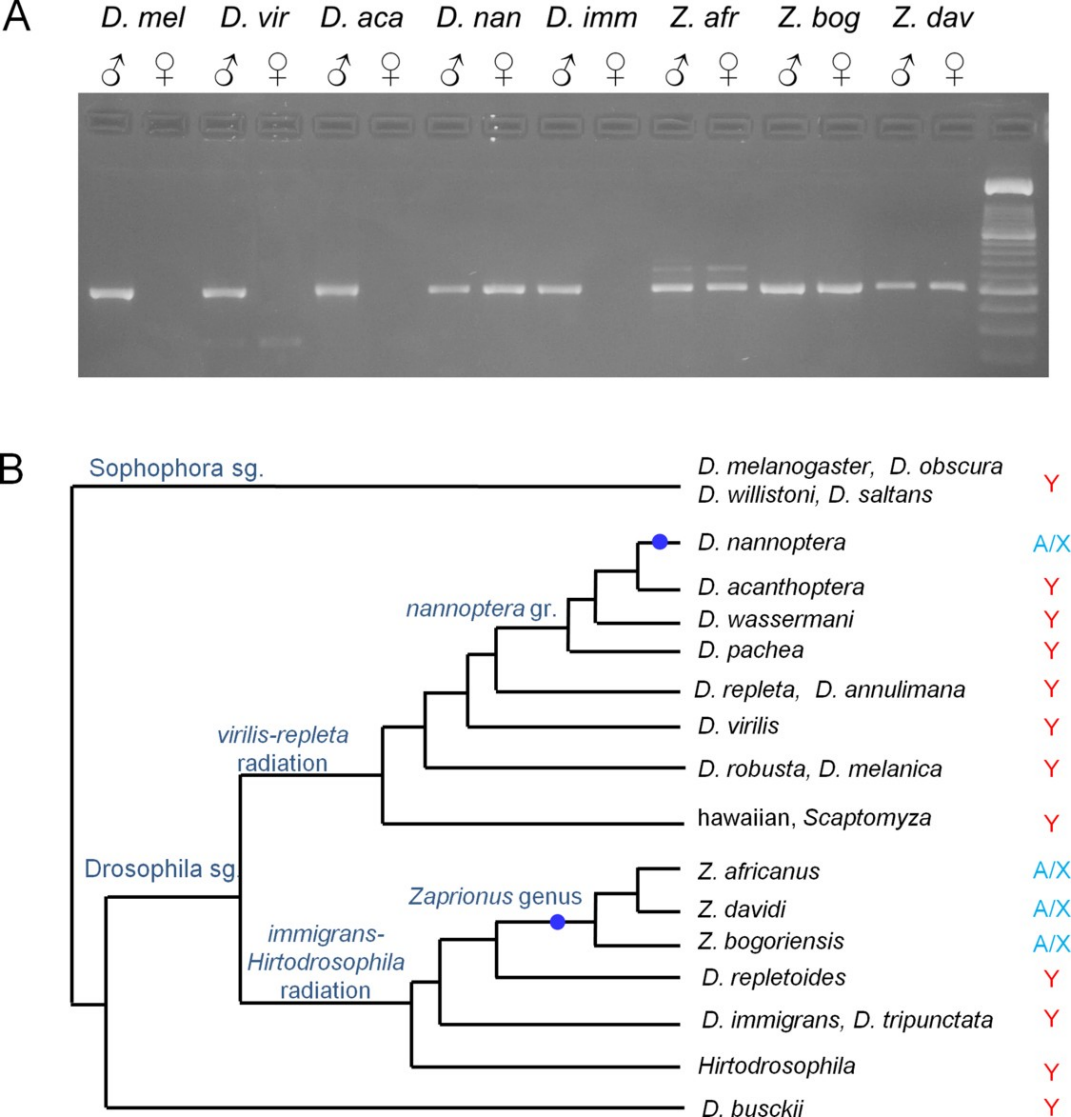
Phylogenetic interpretation of gene movements. A) Degenerated PCR of the *ORY* gene shows that it is autosomal or X-linked in three *Zaprionus* species and in *D*. *nannoptera*, and Y-linked in the remaining species. Species in the order of appearance: *D*. *melanogaster*, *D*. *virilis*, *D*. *acanthoptera*, *D*. *nannoptera*, *D*. *immigrans*, *Z*. *africanus*, *Z*. *bogoriensis*, and *Z*. *davidi*. B) Given the known phylogeny of the species [66,70] the data is best explained by two individual gene movements (blue dots) of the *ORY* gene from the Y chromosome to an autosome or the X ("A/X"), *ORY* being ancestrally Y-linked; the alternative hypothesis of *ORY* being ancestrally A/X implies more than 10 movements to the Y. Note also that the A/X status of the three *Zaprionus* species traces back to only one independent event, at the root of the genus (marked with the blue dot). For the sake of clarity we omitted the Y chromosome incorporation events. PCR primers: ORY_YKNI_F1 / ORY_IEKE_R1 (500 bp).

As we are searching for potentially rare events we used the largest possible sample (400 species;Table A in S1 File). All these species are phylogenetically nested within the *Drosophila* genus, although some of them formally belong to other genera (*e*.*g*., *Zaprionus*; Methods); for the sake of simplicity we will refer to all of them as *Drosophila*. Genome sequencing at this scale is too expensive, so in order to detect Y incorporations we used degenerate PCR, targeting nine known Y-linked genes (Methods). As shown in Fig 1, in Y incorporations all tested genes shift from male-specific PCR amplification (Y-linkage) to male and female amplification (X or autosomal linkage; hereafter, "X/A" linkage; [21]). Besides this large-scale events, there are also individual gene losses from the Y, which in *Drosophila* occur by two mechanisms: (*i*) more commonly, the formerly Y-linked gene moves to an autosome (or the X), in which case a single gene shifts to male and female amplification ("Y chromosome gene transfer"); (ii) the formerly Y-linked gene is lost from the genome, in which case PCR systematically fails in both sexes ("genomic gene loss") [28]. The PCR approach has some limitations, but they do not affect the detection of Y incorporations (Supporting Information). It was very reliable: 94% (3378/3593) of the degenerate PCRs worked (Table B in S1 File), and we readily detected the previously known Y incorporation of the *D*. *pseudoobscura* lineage (Table A in S1 File). These raw PCR results (exemplified in Fig 2A for the *ORY* gene) must be placed into a phylogenetic context in order to identify the independent events (Fig 2B). An analogous procedure was applied to the nine genes in the 400 species (Table A in S1 File). Overall, in the whole dataset we found 21 independent events in which one or more formerly Y-linked genes shifted from male-only to male-female PCR amplification (Table C in S1 File).

### Events affecting one Y-linked gene

A total of 17 Y chromosome gene loss events affected only one gene; 16 of them were Y chromosome gene transfers, and one was a genomic gene loss (the *Ppr-Y* gene in Hawaiian *Drosophila*; ref [28]). Individual gene losses are a well known phenomenon in Y chromosome evolution in *Drosophila* and other organisms [6,28,30]; in the 400 species dataset our best estimate of the rate of gene loss is 0.000985 gene lost / gene / Myr (95% CI: 0.000525–0.001685), which is not significantly different (*P* > 0.5; two-tailed exact test for the ratio of two Poisson means; ref [31]) from a previous estimate based on a much smaller sample (0.001026 gene lost / gene / Myr, 95% CI: 0.000124–0.003707; [14,28]; Supporting Information). We also detected some gene gains by the Y (Supporting Information), but since they cannot be unbiasedly detected by our PCR approach they will not be further discussed.

Besides these 17 events that affected one gene, we found one event simultaneously affecting two genes: in the ancestor of the *obscura* group the *PRY* and *JY-alpha* genes most likely moved from the Y chromosome to the X in a single event, because the two genes are side-by-side on the *D*. *pseudoobscura* and *D*. *obscura* X-chromosomes [32,33]; it would be unlikely that two independent events moved these formerly Y-linked genes to another chromosome in the same position (Fig A in S1 File). Given this, we conservatively counted the *PRY* / *JY-alpha* movements as a single gene loss (counting as two losses, or excluding them, does not change any conclusion).

### Events affecting all Y-linked genes

In three events all (or nearly all) genes formerly present in the Y chromosome became present in females. (Table C in S1 File). These are the most interesting cases, since they strongly indicate Y incorporations. The three cases are described below.

The case of the *D*. *pseudoobscura* lineage is known [21], and indeed was the main motivation for the present study.

The second event affected one species of the *repleta* group, *D*. *limensis*. However, examination of the mitotic chromosomes strongly indicates that *D*. *limensis* does not represent a naturally occurring case of Y incorporation, but rather a laboratory artifact which happened along the decades in which the *D*. *limensis* stock has been maintained in culture (Supporting Information).

**Fig 3:**
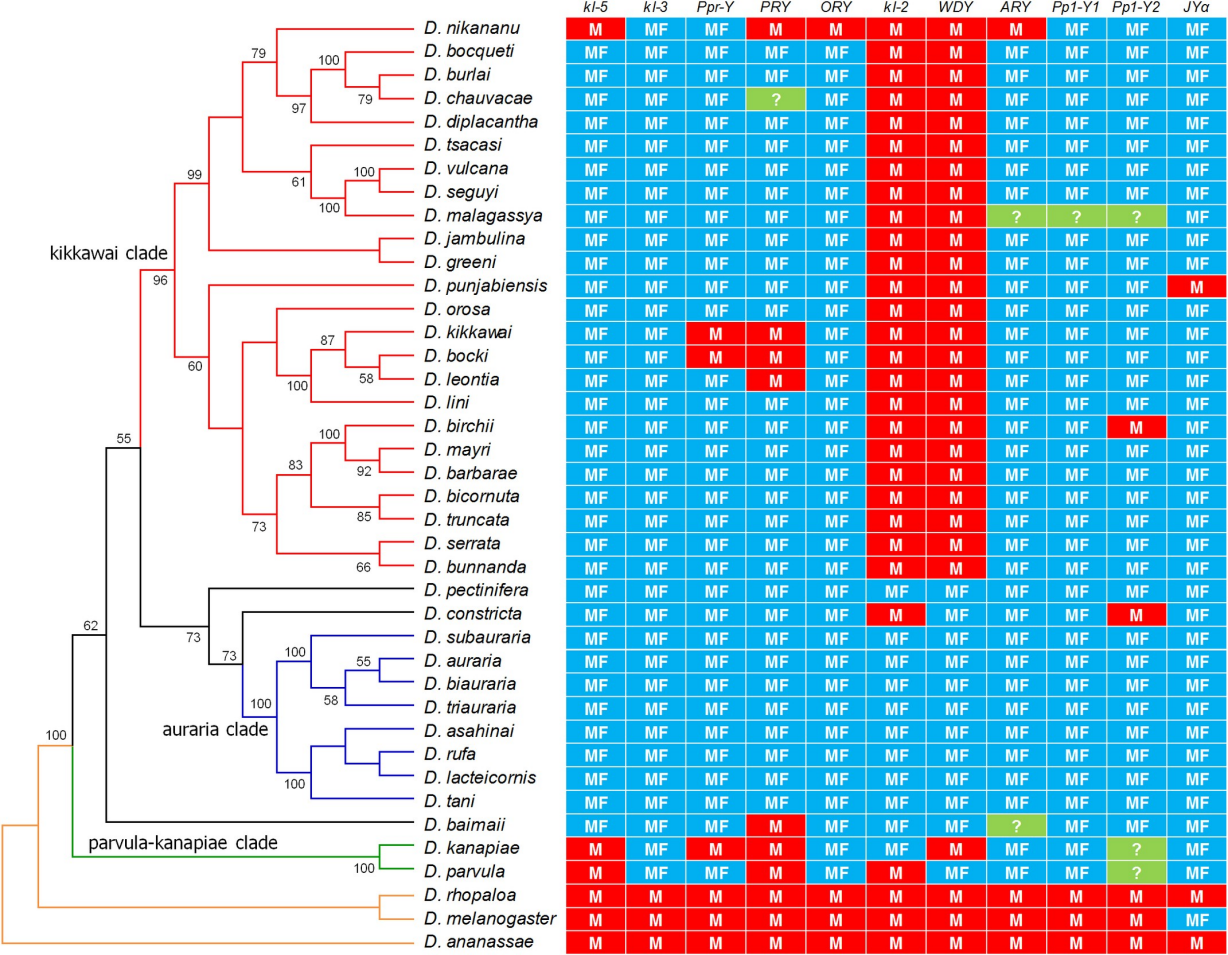
Phylogeny and gene content of the Y chromosome in the *montium* subgroup. “M” means amplification only in males (*i*.*e*., Y-linkage), whereas “MF” means amplification in both sexes (autosomal or X-linkage). The ancestral state of the *montium* subgroup is inferred to be similar to *D*. *ananassae* and *D*. *rhopaloa* (*i*.*e*., all 11 genes Y-linked; orange branches). Note that all species of the “clade *kikkawai*” (red branches) have *kl-2* and *WDY* genes only in males, while the species of the “clade *auraria*” (blue branches) all ancestrally Y-linked genes are present in both sexes. The basal clade “parvula-kanapiae” (green branches) shared *kl-5* and *PRY* genes only in males. Phylogeny based on *Amyrel* gene sequences (ML, bootstrap 1,000 replicates).

The third event affected nearly all tested species of the *montium* subgroup (*melanogaster* group), and hence presumably occurred at the common ancestor of this large taxon (~100 described species;[34]). The event clearly affected the whole Y chromosome, since in many species all genes that are Y-linked in the *montium* ancestor became present in females (Fig 3). Many *montium* species have been investigated cytogenetically; all are XY/XX and have an X chromosome with a single arm [35,36]. These observations make the *montium* case very interesting, because the absence of a two-armed X implies that the current Y chromosome cannot be a neoY chromosome, as has been suggested for *D*. *pseudoobscura* [21,22]. As detailed in the next section, the case of the *montium* subgroup proved to be even more interesting.

### Y incorporation in the *montium* subgroup

As we sampled more *montium* species and genes we obtained an initially puzzling result: most of the ancestrally Y-linked genes sporadically "re-acquire" their Y-linkage along the phylogeny. For example, *ORY* is Y-linked in one of the 40 tested species; *PPr-Y* in three species, and so forth. We attributed these initial cases to independent re-acquisition of male genes by the Y chromosome, but in order to clarify the phenomenon we made an effort to increase the number of *montium* species (by asking colleagues and stock centers) and tested genes (by adding non-degenerate primers based on the *D*. *kikkawai* genome sequence [37]; Supporting Information). We also made a molecular phylogeny of the *montium* species we studied, using *Amyrel* gene sequences produced by DaLage and co-workers [34] and by ourselves (accession numbers MH707309- MH707325). The resulting phylogeny is similar to previous studies [38,39], which do not contain all species we tested. Fig 3 integrates the phylogeny and Y-linkage data. Three main phylogenetic clades of *montium* species appear, and each has a characteristic Y-linked gene content. In the *auraria* clade (eight species in our sample) all formerly Y-linked genes have A/X linkage, supporting the pattern of "complete Y incorporation" seen before in *D*. *pseudoobscura* [21]. In the *kikkawai* clade (24 species) the *WDY* and *kl-2* genes are always Y-linked; besides them, several other genes are Y-linked in a few scattered species. The third phylogenetic clade, composed by *D*. *kanapiae* and *D*. *parvula*, has *kl-5* and *PRY* (as well as several other genes) on the Y chromosome. The gene content of the Y chromosomes seems to have no correlation with their morphologies, which are quite variable among *montium* species (Table D in S1 File). Similarly, *D*. *lini* and *D*. *kikkawai* have been shown to carry B chromosomes [40,41], which are non-essential supernumerary chromosomes that have been suggested as a possible origin of the *Drosophila* Y chromosome [17,42]. Note that the Y-linked gene content of these two species (Fig 3) is similar to other species of the "*kikkawai* clade" (which do not carry B chromosomes); this suggests that the two phenomena (changes in Y-linked gene content and presence of B chromosomes) are unrelated, although we must note that we have not examined the specific strains used in the present work for the presence of B chromosomes. Finally, using the *D*. *melanogaster* Y chromosome cytogenetic map as a proxy for the *montium* species, we found no association between the changes in linkage shown in Fig 3 and the cytogenetic position of the genes (Fig B in S1 File).

We can summarize the pattern we found in *montium* species as follows: previously Y-linked genes seem to "re-appear" in the Y chromosome, closely related species tending to share the same genes on the Y (Fig 3). Before further investigation of this pattern it is worth to re-examine the *D*. *pseudoobscura* case.

### Y incorporation in the *D*. *pseudoobscura* lineage revisited

**Fig 4:**
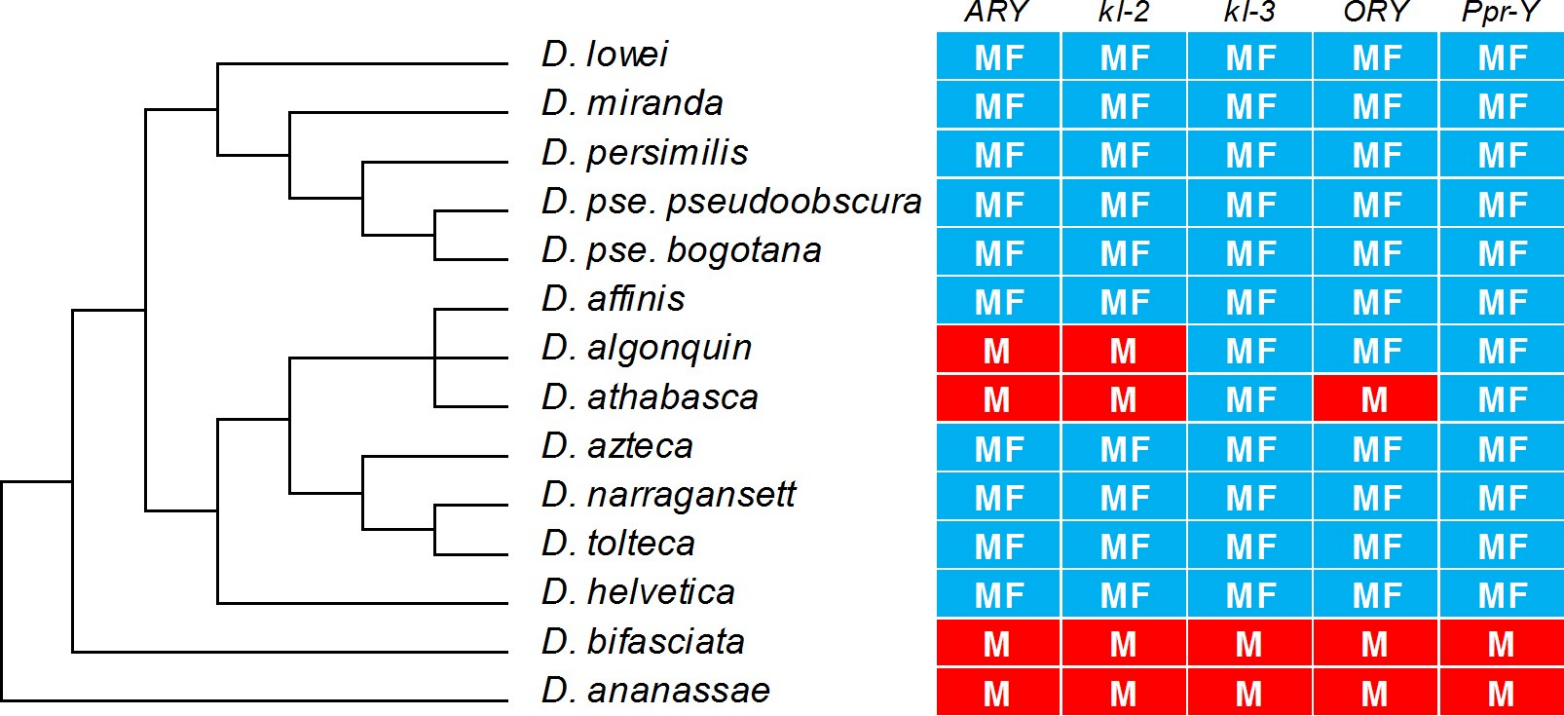
Phylogeny and gene content of the Y chromosome in the *obscura* group. “M” means amplification only in males (*i*.*e*., Y-linkage), whereas “MF” means amplification in both sexes (autosomal or X-linkage). The ancestral state is all genes in the Y chromosome, and is represented by *D*. *ananassae* (*melanogaster* group) and *D*. *bifasciata* (*obscura* subgroup). All remaining species belong to the *pseudoobscura* and *affinis* subgroups, and carry the Y incorporation described in ref [21]. Note that three genes (*ARY*, *kl-2* and *ORY*) seem to have re-acquired Y-linkage in a few species (see Results for the interpretation).Only genes that are Y-linked in the ancestor of the *obscura* group are shown. The *D*. *athabasca* strain came from Rochester, NY, and hence most likely belongs Eastern A subspecies [71]. Phylogeny taken from ref [72].

Previous data strongly suggests that the Y incorporation in the *D*. *pseudoobscura* lineage (*i*.*e*., the *pseudoobscura* and *affinis* subgroups) is complete and irreversible: in all tested species (*D*. *pseudoobscura*, *D*. *persimilis*, *D*. *miranda*, *D*. *azteca*, and *D*. *affinis*) all formerly Y-linked genes (*kl-2*, *kl-3*, *PPr-Y*, *ORY*, and *ARY*) acquired autosomal linkage [21]; mapping and sequencing experiments showed that they moved to the small "dot chromosome" [22], amazingly remaining side-by-side [21,23]. However, given the findings on the *montium* species, one may also think that the *pseudoobscura* lineage is similar to what we would had observed in *montium* if we had sampled only the *auraria* clade (Fig 3). In other words, it is possible that Carvalho and Clark [21] have not sampled enough species to detect the "re-appearance of Y-linkage" in the *pseudoobscura* and *affinis* subgroups. Hence we made an effort to obtain "difficult" species of these subgroups (*D*. *helvetica*, *D*. *athabasca*, *D*. *narragansett*, and *D*. *lowei*; we are indebted to our colleagues listed in Table A in S1 File, who sent us these and other samples). Indeed, we found in two species of the *D*. *pseudoobscura* lineage the same "re-appearance of Y-linkage" phenomenon we observed in the *montium* subgroup (Fig 4).

### Why genes seem to return to the Y chromosome after Y incorporations?

As we commented before we initially attributed these sporadic re-acquisitions of Y-linkage to the normal process of acquisition of male genes by the *Drosophila* Y chromosome. However, in the whole set of *montium* species, nine out 11 genes are Y-linked in at least one species (Fig 3). This seems too frequent to be explained by independent gene gains in a rather short time span (the *montium* / *melanogaster* split occurred 41 Myr ago; [43]), unless the gene gain rate by the Y chromosome is very high in the *montium* subgroup. We formally evaluated this hypothesis as follows. Suppose that the *montium* data is explained by a high rate of A/X to Y gene movements. This being true, the *montium* Y should had acquired other male genes besides the nine mentioned above. In order to test this, we took a sample of male genes that are autosomal in the ancestor of the *montium* subgroup, and that can be acquired by the Y (because they are Y-linked in other *Drosophila* species), and check if they moved to the Y of *montium*. We tested four such genes (Y-linked in *D*. *virilis*: *CG11719*, *CG2964*; Y-linked in *D*. *willistoni*: *CG18155*, *CG14339*; ref [14]), and in all four genes there is no case of A/X to Y movement in the whole set of 40 *montium* species. The difference between 9 genes out 11 and 0 genes out 4 is statistically significant (*P* = 0.011, Fisher exact test; Table 1; see also Supporting Information, section "Statistical test of the re-acquisition of Y-linked genes"). Hence the re-appearance of Y-linkage cannot be explained by a high gene gain rate by the Y chromosome, because only genes that formerly were Y-linked are affected.

**Table 1:** Re-appearance of Y-linked genes in the *montium* subgroup.

	Moved to the Y	Not moved to the Y
Genes present in the *montium* ancestor Y ^a^	9 ^b^	2 ^c^
Genes absent in the *montium* ancestor Y	0	4 ^d^

The hypothesis of general increase in gene gain is rejected (*P* = 0.011; two-tailed Fisher's exact test); only formerly Y-linked genes seem to move to the Y.

^a^ Fig 3 data

^b^ genes *kl-2*, *kl-5*, *PRY*, *PPr-Y*, *ORY*, *WDY*, *Pp1-Y2*, *ARY*, *JY-alpha*

^c^ genes *kl-3*, *Pp1-Y1*

^d^ genes *CG11719*, *CG2964*, *CG14339*, *CG18155*

A similar observation holds for the *D*. *pseudoobscura* lineage: again there is statistically significant evidence that only formerly Y-linked genes re-acquired Y-linkage (*P* = 0.035, Fisher exact test; Table E in S1 File).

**Fig 5:**
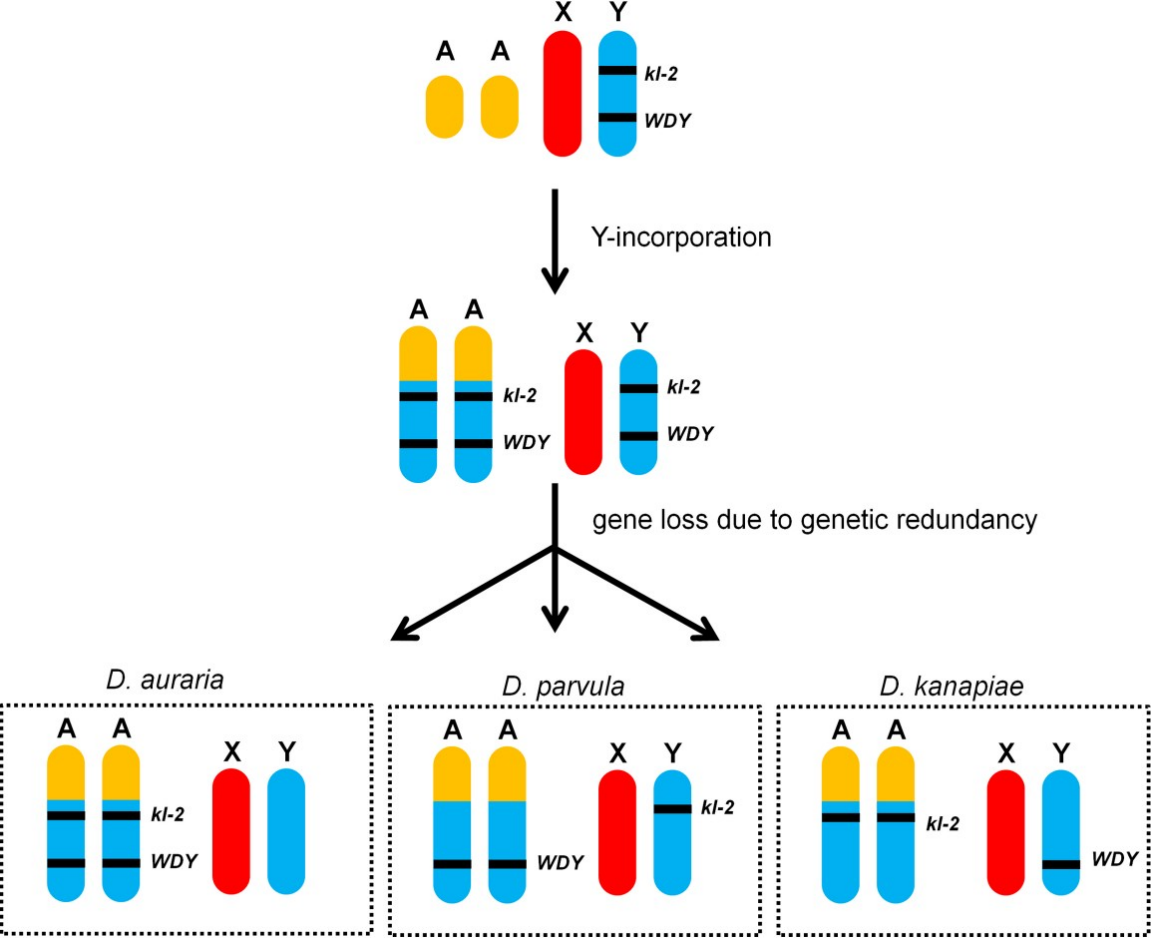
The duplicated Y hypothesis as an explanation for the re-appearance of Y-linkage after Y incorporations. The ancestor of the *montium* subgroup was similar to *D*. *melanogaster* (top). Then an incorporation of the Y chromosome into an autosome would have generated an intermediate stage with two genetically redundant copies of each formerly-linked gene (middle). Random losses of one redundant copy of each gene would result in species like *D*. *auraria*, which is devoid of any Y-linked gene, and *D*. *parvula* and *D*. *kanapiae*, in which some genes seem to have re-acquired Y-linkage (bottom). This hypothesis explains why only formerly Y-linked genes seem to have moved to the Y chromosome (Table 1), and why different *montium* species may carry different subsets of these Y-linked genes (Fig 3); it also explains the *D*. *pseudoobscura* lineage data (Table E in S1 File and Fig 4).

Using the *montium* species as an example, the most parsimonious hypothesis to explain the pattern found in both cases is that a free copy of the Y remained in the genome when the Y chromosome was incorporated into an autosome (or the X) in the ancestor of the *montium* subgroup (Fig 5). Each formerly Y-linked gene would then have two non-allelic copies: the Y-linked and the A/X-linked one. The outcome of this genetic redundancy would have been a more or less random loss of either the free Y copy, or the A/X-linked one [44]. This process would have occurred along with the diversification of the *montium* subgroup, generating the current pattern in which previously Y-linked genes seem to "re-appear" in the Y chromosome, closely related species tending to share the same genes on the Y (Fig 3). As shown in Fig 5, by chance some species would had lost all Y-linked copies (*e*.*g*., the *auraria* clade), whereas others would had lost most of the autosomal copies (*e*.*g*., *D*. *nikananu*). The same explanation, called hereafter the "duplicated Y" hypothesis, applies to the *D*. *pseudoobscura* lineage. We will return to this point in the Discussion.

## Discussion

Incorporation of the Y chromosome into other chromosomes is a very interesting phenomenon with important consequences: (i) formerly male-restricted genes revert to autosomal inheritance and become present in females; (ii) the species may shift from a XY/XX to X0/XX sex-chromosome system, or a new Y chromosome may appear; (iii) the evolutionary forces and conditions that shaped Y chromosome evolution (lack of recombination; male-restricted status, reduced effective population size) disappear or are reversed. These changes have a direct bearing on important aspects of sex-chromosome evolution theory, such as the role of sex-antagonistic genes on the evolution of Y chromosomes [45,46], and the origin of Y chromosome itself.

### Y incorporations occurred at least twice in the *Drosophila* clade

The present work was motivated by the finding that in the second sequenced *Drosophila* species (*D*. *pseudoobscura*; ref [47]) there was a Y incorporation [21]. This strongly suggested that other cases exist, and that perhaps it is a common phenomenon. The answer to these two questions are respectively "yes" and a qualified "no": while studying 400 species (6,529 Myr of evolution), we indeed found one additional case of Y incorporation (the *montium* subgroup); on the whole, 13% of our sample (52 species out of 400) has a Y incorporation; the rate is 0.000893 Y incorporations / Myr (Supporting Information). This rate is similar to the rate of individual gene loss per gene calculated above for the same 400 species, *i*.*e*., a given *Drosophila* Y-linked gene has roughly the same chance of being lost individually, or by a Y chromosome incorporation event. Y chromosome incorporations, as individual gene losses in the *Drosophila* Y, are rather rare events; however, if we consider a broader time scale (*e*.*g*., the Diptera order) they may be frequent enough to explain, for example, the lack of homology between the Y chromosome of *Drosophila* and more distant Diptera such as Tephritidae and mosquitoes [28,48]. Analogously, the identity of the X chromosome (Muller element A) is conserved across the Drosophilidae family, but not in Diptera [49].

Are there additional cases of Y incorporations in the phylogenetic clade studied here? Probably yes. For example, although not represented in our sample, there are *Samoaia* and *Hirtodrosophila* species with X0/XX sex determination [24,50], in which presumably the ancestral Y (and its genes) was incorporated into another chromosome (the *Samoaia* and *Hirtodrosophila* species we sampled have XY males, and the normal gene content in the Y; Table A in S1 File). This is exactly what we found in X0 *Chymomyza* species (*e*.*g*., *C*. *procnemis*; ref [51]): the ancestrally Y-linked genes are present in males and females (unpublished).

It is also interesting to consider the relevance of Y incorporations outside *Drosophila*. The discovery of Y incorporations usually requires genome sequencing and a detailed investigation of the Y-linked genes, so they easily went unnoticed, as shown by the cases of *D*. *pseudoobscura* (a species studied since the 1920's) and *D*. *kikkawai* (which Y incorporation was not detected, despite the species being sequenced a few years ago; [37]); this suggests that there may be cases even in fairly well known species. On the other hand, species with a sex-determining Y chromosome (*e*.*g*., many vertebrates and Diptera) most likely are less prone to Y incorporations because such events are expected to disrupt the sex-ratio. However, note that in many such groups there had been cases of turnover of the sex-determination system (*e*.*g*., [52–54]), which most likely had similar effects on the sex-ratio. Hence, the relevance of Y incorporations outside *Drosophila* remains to be determined, and it would be desirable to perform similar studies in other groups.

### Natural selection and Y incorporations

Our study offers some clues on the possible fitness advantages of Y chromosome incorporations. Carvalho and Clark [21] found that the Y incorporation is present in the closely related *affinis* and *pseudoobscura* subgroups (which are known to carry an X-A fusion), and is absent in the more distantly related *obscura* subgroup (which does not carry the X-A fusion), and suggested that the Y incorporation was an adaptive response to the X-A fusion. Namely, X-A fusions are known to cause meiotic problems because three (instead of two) centromeres must correctly pair and segregate in males: the X (fused with one member of the autosomal pair), the ancestral Y, and the other member of the autosomal pair (called "neoY"); incorporation of the ancestral Y into another autosome would solve this problem (see Fig 3 of ref [21]). This is an attractive hypothesis because it seems to explain very well the puzzling phenomenon found in the *D*. *pseudoobscura* lineage. However two lines of evidence found here suggest that the causal link between X-A fusions and Y chromosome incorporations is, at best, weak. First, there are 35 species (nine independent events) with an X-A fusion similar to *D*. *pseudoobscura* in our sample of 400 species, and all carry the ancestral *Drosophila* Y (Table F in S1 File). Therefore Y incorporation is not an obligatory consequence of X-A fusions. Second, in the other case of Y incorporation we found (*montium* subgroup) the X chromosome is not fused to any autosome (it has a single arm). Therefore Y incorporations can occur in the absence of X-A fusions. Thus, at this point, the relationship between X-A fusions and Y incorporation is unclear. Related to this, we do not know if Y chromosome incorporations are selection-driven events, as proposed by Carvalho and Clark [21] or, alternatively, mutations fixed by genetic drift; it is also possible that some were selection-driven events (perhaps *D*. *pseudoobscura*), whereas others were fixed by drift (perhaps in species devoid of X-A fusions). It is interesting to note that in other chromosomal mutations such as inversions and translocations the relative roles of selection and drift are also unclear [26,27,55].

Finally, given the data shown in Fig 4 and Table E in S1 File, it seems likely that the current Y chromosome of the *D*. *pseudoobscura* lineage actually is an impoverished copy of the ancestral Y chromosome. If this is true, then the explanation offered by Carvalho and Clark [21] for the Y incorporation would be less compelling (for there would had been a free Y chromosome all the time), but not invalidated: one of the fitness costs caused by the X-A fusion is the generation of some sterile X0 sons; the Y incorporation ameliorates this because it would rescue the fertility of these sons.

### Alternative hypothesis for the re-appearance of Y-linkage in the *montium* and *pseudoobscura* lineages

The duplicated Y hypothesis (Fig 5) is bold, but as far as we can see it is the best explanation for the data. The only alternative hypothesis we could think to explain the pattern shown in Table 1 and Table E in S1 File is that the free Y really disappeared (eventually being replaced by something else) or lost all its genes after its incorporation into an autosome or the X, but there was a high fitness cost of the new location of these formerly Y-linked genes. This would have "accelerated" the movement to the Y (*i*.*e*., increased the fixation probability) only for this set of genes, which would explain the data shown in Table 1 and Table E in S1 File. However, if there is such strong selection favoring the Y-linkage of these particular genes, it is difficult to explain how the Y would get lost (or emptied) at first place.

Perhaps the main difficulty of the duplicated Y hypothesis is that it requires that several genes retained their two copies (A/X and Y-linked) for rather long periods across the phylogeny. For example, the re-appearance of Y-linkage for several genes in *D*. *nikananu* (*kl-5*, *PRY*, *ORY*, *ARY*), coupled with their A/X status in the sister species *D*. *diplacantha*, would require that the two copies of these genes had persisted since the Y incorporation (at least ~19 Myr ago) until the *D*. *nikananu* / *D*. *diplacantha* divergence (~5.9 Myr ago; Fig 3 and Fig C in S1 File). A similar reasoning applies to several other genes and sections of the phylogeny. This retention of duplicate genes for ~13 Myr seems too long, since the half-life of duplicated genes in *Drosophila* has been estimated as 0.66 Myr (ref [56]) and 3.2 Myr (ref [44]). However, there is a major factor that may explain the discrepancy between these half-life estimates and ~13 Myr: while the former were calculated from single gene (or small segment) duplications, in the later a whole chromosome was duplicated. So, in the Y incorporations the genes were duplicated along with all long-range regulatory regions and chromatin state signals, which most likely favors a long survival. In this sense Y incorporations resemble polyploidizations, which are known to have an unexpectedly long preservation of duplicated genes (tens to hundreds of millions of years; ref [57], pp. 207–208, and references cited therein).

### Possible tests of the duplicated Y hypothesis

There are several ways to test this hypothesis. First, as it implies that the locations of the "re-acquired" Y-linked genes actually are ancestral, it predicts that gene order in the Y chromosome of species of the *montium* subgroup (*e*.*g*., *D*. *nikananu* and *D*. *kanapiae*; Fig 3) should be conserved among themselves and perhaps with *D*. *melanogaster* [58,59], although we should keep in mind that nothing is known about the rate of rearrangements in the *Drosophila* Y. Such test must be done by fluorescent in situ hybridization (FISH), or by a long-read assembly [23], since Sanger or Illumina assemblies are too fragmented to provide synteny information of repetitive regions. Second, since two gene copies (Y and autosomal/X-linked) seem to have co-existed for a long time, one might expect to see both in some species. To date *D*. *kikkawai* and *D*. *serrata* are the only *montium* species that has been sequenced [37,60], and we found no signs of two gene copies either in the assembled scaffolds or the raw traces (not shown), but both assemblies are not good for this purpose (see section "Genomic effects of the Y incorporation in *montium* subgroup species"). We are now obtaining improved assemblies of both species to search for duplicated genes. Finally, full identification of the Y-linked genes (*e*.*g*., ref [14]) in the *montium* subgroup and in *D*. *pseudoobscura* (below) from may help to elucidate the origin of their current Y chromosomes. We are now pursuing some of these approaches.

### On the origin of the *D*. *pseudoobscura* Y

**Fig 6:**
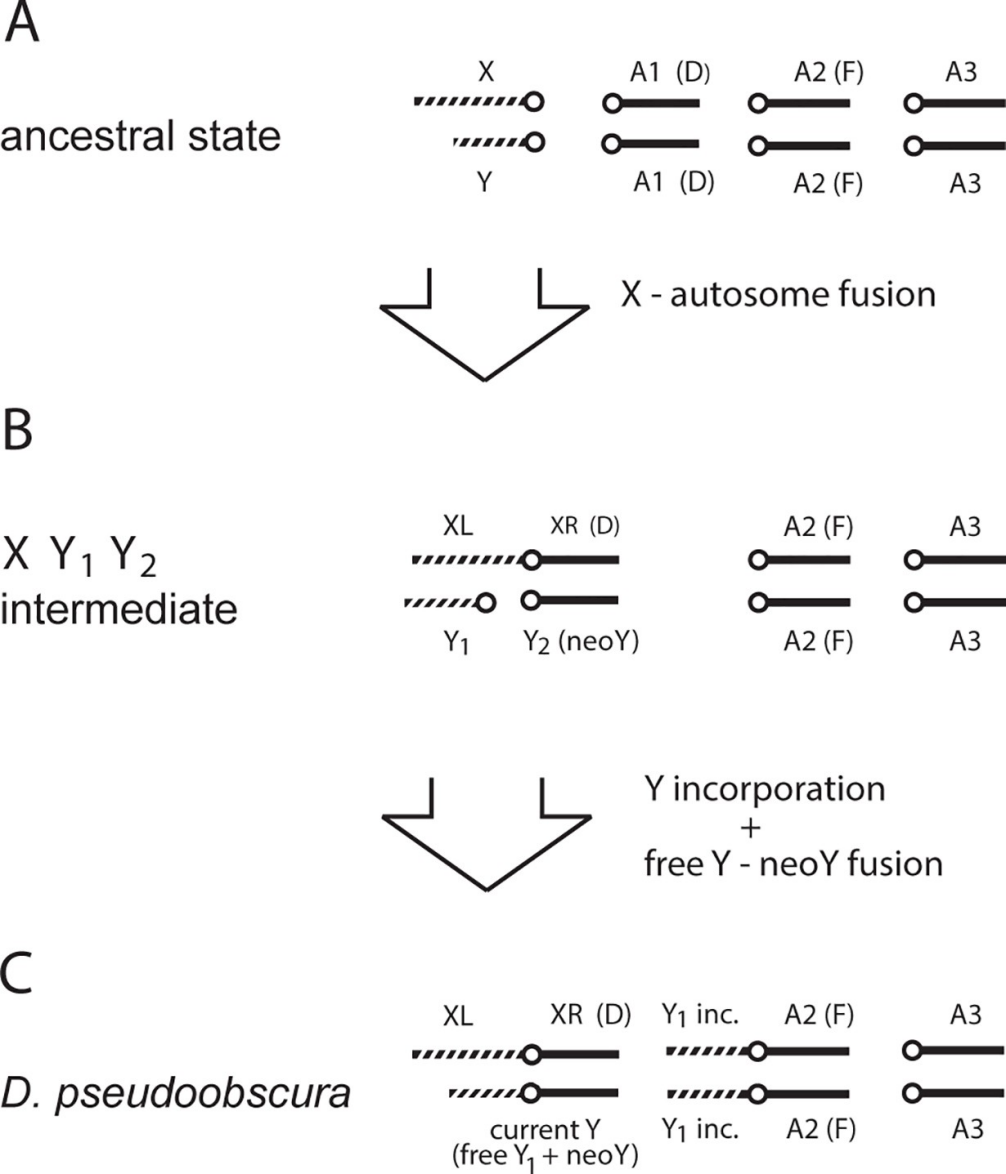
Updated model for the origin of *D*. *pseudoobscura* sex chromosomes. Autosomes are shown as solid bars (shown three pairs, A1, A2 and A3), sex chromosomes as striped bars, and centromeres as open circles. (A) The ancestral state configuration is typical for *Drosophila* (*e*.*g*., *obscura* subgroup species). (B) A centric fusion between the X and one autosome (Muller element D, corresponding to chromosome 3L in *D*. *melanogaster*; [73]) generated an X Y_1_ Y_2_ sex-determination system, which was transient in the *D*. *pseudoobscura* lineage, but exists in other species [26]. (C) A Y incorporation event placed the ancestral Y (Y_1_) within another autosome (Muller element F, corresponding to chromosome 4 in *D*. *melanogaster*; [21–23]). The present work strongly suggests that a copy of the ancestral Y (Y_1_) survived also as a free chromosome and fused with the neoY (Fig 4 and Table E in S1 File), originating the current Y chromosome of the *D*. *pseudoobscura* lineage. The order of the events represented by the two large arrows is hypothetical. Also, the Y incorporation and the free Y—neoY fusion events (represented here by a single arrow) probably were two separate events. Modified from reference [21].

The results described in this work shed light on the origin of the current Y chromosome of the *D*. *pseudoobscura* lineage, a subject that has been somewhat controversial. Carvalho and Clark [21] suggested that it may be the neoY (*i*.*e*., the remnant of the homolog of the Muller-D autosome that got fused with the X), but noted that direct evidence (*e*.*g*., a concentration of Muller D-derived genes in the Y) was lacking, and that other origins are possible. Other authors assumed that the current Y is a neoY. As we noted above, the data presented in this work strongly suggest that the current Y of this lineage is a very impoverished ancestral Y. However, ongoing work in our lab identified four functional protein coding genes in the *D*. *pseudoobscura* Y; three of them have orthologs in *D*. *melanogaster* which are located in the Muller D (*CG6661*, *CG6845*, and *CG32181*; the fourth gene, *GA27172*, does not have a clear ortholog; [61]). So, as shown in Fig 6, the current Y of *D*. *pseudoobscura* lineage seems to be both a remnant of the ancestral Y (as indicated by the "re-appearance" of Y-linkage) and a remnant of the neoY (as indicated by the three Muller-D derived genes). Actually, this dual nature of the *D*. *pseudoobscura* Y was suggested by M.J.D. White more than 40 years ago: "Most probably, this loss of the Y_2_ [the Muller-D derived neoY] took place through its fusion with the original Y (= Y_1_) and subsequent heterochromatinization." (ref [26], p. 350). White was not aware of the incorporation of the ancestral Y into an autosome [21–23], which is a major phenomenon, but other than this his suggestion probably is correct.

### Genomic effects of the Y incorporation in *montium* subgroup species

Y incorporations offer the unique opportunity to study the effect of the forces that shaped the Y chromosome (lack of recombination; male-restricted status; reduced effective population size) as they disappear or are reversed. We will focus here on two characteristics of *Drosophila* Y-linked genes: intron size and intergenic distance. *Drosophila* Y-linked genes contain some Mbp-sized introns, filled with repetitive DNA [59,62]. These huge introns cause assembly breaks, so multi-exon genes are never assembled in a single scaffold; intergenic sequences are also large and repeat-rich, so we seldom observe two genes in the same scaffold [11,28]. Interestingly, in the incorporated Y of *D*. *pseudoobscura* the intron sizes became fairly normal and intergenic distances were reduced [21,23]. As a consequence, even in a Sanger assembly large genes (*kl-2*, *kl-3*) were fully assembled, and most genes are present in scaffolds containing more than one gene [21]. So it seems that after being incorporated into an autosome the former Y is acquiring genomic characteristics of euchromatic regions, *i*.*e*., we would be observing backwards some of the processes of *Drosophila* Y evolution. Larracuente and Clark [63] suggested that positive selection to reduce intron sizes played a role in this process: the incorporated Y chromosome of *D*. *pseudoobscura* shows a strong reduction of genetic variability (which is compatible with recurrent selective sweeps), in the absence of detectable positive selection in the coding sequence of the genes (which suggest that non-coding regions were the target of selection).

The discovery of the case of Y incorporation in the *montium* subgroup allows us to further investigate this phenomenon. As shown in Table G in S1 File (columns 5–6), the incorporated Y region of both *montium* species with sequenced genomes (*D*. *kikkawai* and *D*. *serrata* [37,60]) seems to be much more similar to free Y chromosomes (*e*.*g*., *D*. *melanogaster*) than to the incorporated Y of *D*. *pseudoobscura*: the genes are heavily fragmented (a sign of large, repeat-rich introns) and in only one case is there more than one gene in the same scaffold (a sign of large, repeat-rich intergenic spaces).

If confirmed, the above findings would be particularly interesting because the Y incorporation in *montium*, which occurred 19 to 41 Mya (Fig C in S1 File), seems to be older than the *D*. *pseudoobscura* event (12.7 to 20.8 Mya; ref [64]), and hence the "euchromatinization" would be proceeding with much slower speed in *montium* species, or not occurring at all. However, there is a big caveat: the genome assemblies of both *montium* species are much more fragmented and/or have assembly issues that make them not directly comparable to *D*. *pseudoobscura* (Table G in S1 File, columns 7–8). Namely, *D*. *kikkawai* assembly used short reads (Illumina and Roche/454) which is not good for repetitive regions [65], whereas the *D*. *serrata* PacBio assembly is also fairly fragmented and with other assembly problems in the incorporated Y genes (*e*.*g*., out-of-frame indels and missing exonic sequences; Table G in S1 File). The *D*. *pseudoobscura* assemblies, which were based in Sanger or in PacBio [21,23], do not have these problems. It will be very interesting to study the incorporated Y region in improved assemblies of *montium* species.

## Concluding remarks

The finding of a Y incorporation in the second *Drosophila* species that was sequenced (*D*. *pseudoobscura*; refs [21,22]) strongly suggested that there are other cases, and that this may be a common phenomenon. These questions can only be answered empirically. We did this with a sample of 400 *Drosophila* species, and found one additional case (the *montium* subgroup), besides the previously known event in the *D*. *pseudoobscura* lineage. These two events affect 13% of the sampled species (52/400); they happened in the last ~60Myr, and projected into larger time scale, may explain the complete lack of Y chromosome homology between the *Drosophila* and more distant Diptera such as mosquitoes and Tephritidae [48]. In both the *montium* and *pseudoobscura* lineages the Y incorporation resulted in XY/XX species in which the Y seems to be derived from the ancestral Y chromosome; ongoing work on the *D*. *pseudoobscura* lineage suggests that its Y also contain the remnants of the neoY. Finally, the data presented here show that the formerly suggested adaptive explanation for Y incorporations [21] is not general, and hence this remains an open question. These findings, the discoveries that X chromosomes are also replaced [49], and that the evolution of the *Drosophila* Y is dominated by gene gains instead of gene losses [14,28], show that sex-chromosome evolution in Diptera is a dynamic and complex process, and that there is a lot more to be learned about it.

## Methods

### Species studied and DNA extraction

The 400 Drosophilid species (Table A in S1 File) belong to the following formally recognized genera (subgenera): *Dettopsomyia*, *Drosophila* (*Dorsilopha*, *Drosophila*, *Phloridosa*, *Siphlodora*, *Sophophora*), *Hirtodrosophila*, *Mycodrosophila*, *Samoaia*, *Scaptomyza* (*Bunostoma*, *Parascaptomyza*), *Zaprionus* (*Anaprionus*, *Zaprionus*) and *Zygothrica*, which form a natural group (*i*.*e*., a monophyletic clade) with fairly well known phylogenetic relationships [66,67]. We used all samples we could obtain (Table A in S1 File), provided that we got at least one male and one female from the same species. Most samples came from isofemale lines. Unculturable species were represented by copulating pairs (when available) or by wild-caught material, both identified by an experienced *Drosophila* taxonomist (usually Carlos R. Vilela). DNA was extracted separately from males and females (one to ~ four individuals of each sex); in most cases females may had been inseminated so we used only their thorax to avoid contamination from stored sperm. We used standard phenol-chloroform extraction or the Puregene kit (Qiagen cat # 158667), following the manufacturer's instructions; DNA was quantified with Qubit (Invitrogen cat # Q32857), usually diluted to ~ 1 ng/ul, and frozen at -20 ^o^C until use.

### Gene choice and degenerate PCR

The whole set of species was studied with degenerate PCR primers for nine genes: six Y-linked genes that are present in the ancestral *Drosophila* Y (*kl-2*, *kl-3*, *ORY*, *PPr-Y*, *PRY*, *JY-alpha*), plus the *kl-5*, *WDY* and *CG11719* genes [14,28,68]. These nine genes were chosen by two criteria: being informative (*i*.*e*., Y-linked in a large number of species), and allowing the design of reliable degenerate primers. In the two cases of suspected Y incorporation, additional genes (that do not yield reliable degenerate primers) were tested for Y-linkage with normal PCR primers, designed using a closely related sequenced species: genes *Pp1-Y1*, *Pp1-Y2* and *ARY* in the *montium* subgroup (based on the *D*. *kikkawai* genome sequence), and the *ARY* gene in the *pseudoobscura* lineage (based on *D*. *pseudoobscura*). Note that Fig 3 shows only 11 genes (instead of 12) because the *CG11719* gene is non-informative in the Sophophora subgenus (it is ancestrally autosomal). Degenerate primers were designed using the Codehop method [69], targeting protein regions that are conserved among the 12 sequenced *Drosophila* species (Supporting Information). The PCR protocols were deposited in dx.doi.org/10.17504/protocols.io.szyef7w.

### Confirmation of PCR results

As contamination of female DNA by male DNA, or spurious PCR amplification might lead to errors in linkage ascertainment (namely, a Y-linked gene be considered autosomal / X-linked), all suspected cases of gene movements were confirmed with at least three PCR experiments, using different strains when available, and also re-testing the closest related species. If they passed these tests we confirmed the result by sequencing the PCR product in females or in both sexes (Supporting Information). Another layer of error control is provided by phylogenetic consistency: among the 21 independent events of linkage changes, 11 affected several or many species, which provides a cross-check. As can be seen in Table A in S1 File, the data are highly consistent. All sequencing was performed at Macrogen (Korea).

## Supporting information

S1 File Supporting information. (DOC)

S1 Dataset Excel file used to estimate the loss rate of Y-linked genes. (XLS)
