# Supplementary material for: An investigation of Y chromosome incorporations in 400 species of Drosophila and related genera
Source: PLoS Genet. 2018 Nov 2;14(11):e1007770. doi: 10.1371/journal.pgen.1007770 (PMC6235401; doi:10.1371/journal.pgen.1007770)
Supplement: S1 File — (DOC) [file pgen.1007770.s001.doc]

v.5nov2018

**Supporting Information**

**E. G. Dupim et al. An investigation of Y chromosome incorporations in 400 species of *Drosophila* and related genera**

Index

[Limitations of the degenerate PCR approach. 2](#__RefHeading___Toc524538646)

[Estimation of the length of the 400 species tree. 4](#__RefHeading___Toc524538647)

[Estimation of the gene loss rate of the Y chromosome. 6](#__RefHeading___Toc524538648)

[Gene gains by the Y chromosome in the 400 species sample. 8](#__RefHeading___Toc524538649)

[The case of *D. limensis*. 8](#__RefHeading___Toc524538650)

[Statistical test of the re-acquisition of Y-linked genes. 9](#__RefHeading___Toc524538651)

[Gene choice and primer design. 10](#__RefHeading___Toc524538652)

[Confirmation of identity of PCR products. 12](#__RefHeading___Toc524538653)

[Supporting Figures 14](#__RefHeading___Toc524538654)

[Supporting Tables 20](#__RefHeading___Toc524538655)

[References 47](#__RefHeading___Toc524538656)

Limitations of the degenerate PCR approach. The degenerate PCR approach allows large samples, and will very reliably detect Y chromosome incorporations, which is the main purpose of the present paper. However, it has some limitations while detecting events affecting individual genes when compared with the previous approach, which requires genome sequencing .

Regarding individual gene gains by the Y chromosome, the degenerate PCR method can detect some of them, but it is not possible to obtain an unbiased estimate of the rate of gene gain. The reason is that it can detect gene gains by the Y only if the specific gene is Y-linked in either *D. melanogaster* or *D. virilis* (given that we designed PCR primers only for this class of genes; see SI section "Gene choice and primer design") and autosomal or X-linked in other branches of the phylogeny (so we can observe a A/X to Y movement). These factors lead to a gross underestimation of the gene gain rate, an error that can only be avoided by the exhaustive identification of Y-linked genes. An example may help here: using an efficient method for the identification of Y-linked genes, Carvalho and Clark detected four A/X to Y movements in the *D. virilis* lineage, whereas only one (affecting the *kl-5* gene) would had been detected by the "PCR approach" targeting the previously known Y-linked genes from *D. melanogaster*. Although not useful to estimate gene gain rate, the cases of gene gains detected here provide useful information about Y chromosome gene composition, so we listed them in Table H in S1 File and SI section "Gene gains by the Y chromosome in the 400 species sample".

Regarding individual gene losses by the Y chromosome there are three limitations.

(i) If the gene loss from the Y was caused by a genomic gene loss (instead of by a movement to an autosome or the X) and if it affects very few species, it probably will go undetected because it would be difficult to tease it apart from a simple PCR failure. Note, however, that events affecting a lineage are reliably detected (*e.g.*, the *Ppr-Y* gene loss in the Hawaiian *Drosophila*, described in ref ; see Table A and Table C in S1 File). Furthermore, half of the Y-linked genes of *D. melanogaster* (the best known species) are essential for male fertility , and hence are very unlikely to be lost from the genome, which attenuates this source of error.

(ii) Even a gene loss caused by a gene movement from the Y to an autosome or the X may be undetected if by chance the degenerate PCR failed for this particular species and gene. Specifically, as on average 6% of the degenerate PCR failed (215/3593; Table B in S1 File), the number of detected gene losses in the Y due to gene movements is expected to be underestimated by this value (we would had found 19 losses, instead of 18).

(iii) PCR would not detect a Y-linked copy of a gene in the presence of an autosomal or X-linked copy. This situation is expected to occur shortly after a gene movement to or from the Y (before one of the two copies degenerate due to genetic redundancy; ref. ), and indeed has been detected by sequencing in two cases: the *FDY* gene of *D. melanogaster* and the *kl-5* gene of *D. orientacea* . However, except for very recent movements such as the above cases, this limitation probably is not important: no duplicated genes (or pseudogenes) associated with individual Y-linked gene movements have been detected in the 12 *Drosophila* genome sequences , implying a fast elimination of one of the two copies of the genes.

The third limitation probably is negligible. The first two, while probably not quantitatively important, will cause a downward bias in the estimated rate of gene losses. Although it is not possible to precisely estimate this bias, we may approach the problem by recalculating the gene loss rate using only essential genes (which abolishes the first limitation), with very high PCR success rate (which reduces the second). These high-confidence genes are *kl-2*, *kl-3*, *kl-5* and *WDY*; their loss cause male sterility, and their average PCR success is 98.9% (1582/1600). If the first two limitations were causing a relevant downward bias we would expect that the estimated loss rate would increase if we use only the high-confidence genes, but the opposite happens (gene loss rate per gene using all nine genes: 0.000453 genes lost / gene / Myr ; using high confidence genes: 0.000171 genes lost / gene / Myr ; the difference is not statistically significant: *P*=0.15, Poisson conditional test; see also SI section "Estimation of the gene loss rate of the Y chromosome"). Hence the available data suggest that the number of detected gene losses in the present work is close to the real one (as we will see in SI section "Estimation of the gene loss rate of the Y chromosome" there is more uncertainty in the *rate* of gene loss, caused by uncertainties in the tree length).

We must stress that none of limitations discussed above apply to the Y incorporations because they would be detected even if, say, half of the genes have no data due to degenerate PCR failure.

Estimation of the length of the 400 species tree. In order to estimate the rate of Y incorporations and the rate of gene loss by the Y we would need the total tree length (in Myr) in the 400 species phylogeny. Unfortunately, there is no gene that has been sequenced in all 400 species, which precludes the use of a simple molecular clock. However, the divergence times among several species are approximately known (; Fig D in S1 File), and may be used as a "backbone" to estimate the total tree length, using either a simple Yule model or the more sophisticated PASTIS method .

The first solution uses the divergence times shown in Fig D in S1 File and assume that diversification at each terminal node followed the Yule model. For example, the *ananassae* subgroup (16 species in our sample) diverged from *melanogaster* 44.2 Myr ago ; according to the Yule model, a lineage with 16 species that started diversifying from a common ancestor 44.2 Myr ago has an expected total branch length of 297.6 Myr (Eqn(5) of ref . The total tree length estimated by this method is 4,907 Myr.

The above method in unnecessarily crude because it ignores any phylogenetic information that is available about the diversification at each terminal node of Fig D in S1 File. For example, in most cases there is sequence information for at least several species, and those without it may be known to belong to (or to be excluded of) some monophyletic group. The PASTIS method combines in a Bayesian framework all this partial phylogenetic information to estimate a tree. We run PASTIS using sequences from the *Amyrel* gene, which DaLage and co-workers have sequenced in many *Drosophila* species, the backbone shown in Fig D in S1 File, and phylogenetic information extracted from the literature. The estimated total tree length was 6,529 Myr. As a consistency check, we also estimated the total branch length using a standard molecular clock approach and compared it with the Yule and PASTIS results (this could only be done for clades where we have sequences of the same gene (*Amyrel*) in all or nearly all species). As can be seen in Table I in S1 File, the PASTIS and particularly the Yule methods seem to over-estimate the tree length, although we must take into account that divergence time estimates have wide sampling errors.

Finally, we may estimate the tree length using only the species for which we have *Amyrel* sequences (this is the gene with wider coverage of *Drosophila* species), which circumvents the need of PASTIS (or Yule's model), at the price of excluding part of our sample. Using the RelTime molecular clock and the available *Amyrel* sequences (184 species), we obtained a total tree length of 2,240 Myr. This value cannot be compared with the previous ones (since it uses a smaller number of species), but will be used in SI section "Estimation of the gene loss rate of the Y chromosome" to estimate a loss rate without the possible bias brought by PASTIS or Yule's model.

It worth to finish by noting that the uncertainty in the estimates of *Drosophila* divergence times (e.g., we used as the reference backbone tree is not a problem here because our main point is to compare the previous estimates of gene loss with the present one; both are based on the same divergence time estimates , and hence the errors in divergence time cancel out.

Estimation of the gene loss rate of the Y chromosome. Given the total tree length (SI section "Estimation of the length of the 400 species tree") and the number of gene losses (Table C in S1 File), we can estimate the gene loss rate as follows. As mentioned in the main text, we conservatively counted the *PRY* / *JY-alpha* movements in the *obscura* group as a single gene loss; counting as two losses, or excluding them, does not change any conclusion.

Since we are not following all Y-linked genes in the 400 species (either because we do not know them, or because they were not included in the set of nine genes we tested), it is better to measure the loss rate in "genes lost per gene per Myr", instead of "genes lost / Myr": if we had sampled more than nine genes, we certainly would had observed more than 18 events of gene loss, but this dependency disappears by measuring gene loss as "genes lost per gene per Myr". In order to obtain this quantity, as detailed in ref , the number of observed gene losses (18 in our case) must be divided by the product "number of sampled genes × branch length", which is called *exposure* in the context of Poisson regression . Supporting Dataset_S1.xls details these procedures; we have 18 individual gene losses and a total exposure of 39773.1 genes × Myr, which yields a loss rate of 0.000453 genes lost / gene / Myr.

Two previous estimates are available : 0.001026 (2 losses; exposure: 1948.6; *D. melanogaster* data and 0.001645 (3 losses; exposure 1823.8; *D. virilis* data). These values are more easily compared in Fig E in S1 File. The differences between these previous values and the present one are not statistically significant (*P*=0.48 and *P*=0.12, respectively; Poisson conditional test). As the nominal values of previous estimates are 2.3 to 3.6 fold higher than the estimate presented here (albeit, as noted before, the differences lack statistical significance), it is useful to consider which one is expected to be closer to the truth.

The present estimate was based on a much larger sample size than the previous ones (400 species, exposure of 39,773.1 versus 12 species, exposure of 1,948.6 and 1,823.8 , respectively), and hence has a much lower sampling error (note the 95% confidence intervals in Fig E in S1 File). However, as we discussed before, it suffers from downward bias from two sources, which effect we evaluated as follows.

The first bias is an underestimation of the number of gene losses due to the occasional failure of degenerate PCR. As detailed in the SI section "Limitations of the degenerate PCR approach", this may be mitigated by estimating the gene loss ratio with a set of four high confidence genes that are much less prone to this problem. Using them we obtained a gene loss rate that is even smaller (0.000171 gene lost / gene /Myr), which strongly suggest that this is not a relevant bias.

The second possible bias is an over-estimate the tree length by the PASTIS method, which was used to allow the inclusion of species without sequenced genes suitable for a molecular clock (SI section "Estimation of the length of the 400 species tree" and Table I in S1 File). We addressed this possible bias by removing these species; the reduced dataset has 184 species (total tree length: 2,240 Myr; exposure: 13,194.6), in which we observed 13 gene losses. The gene loss rate is 0.000985 (95% CI: 0.000525- 0.001685), which is more similar to previous estimates based on sequenced genomes . Given the arguments presented in this section, we think it reasonable to consider this the current best estimate of the rate of gene loss by the *Drosophila* Y.

Using the same set of 184 species, the rate of Y chromosome incorporation is 0.000893 incorporations / Myr (2 events per 2,240 Myr; 95% CI: 0.000108-0.003225), which is similar to the above estimated rate of gene loss (0.000985gene lost / gene /Myr). In other words, a *Drosophila* Y-linked gene has approximately the same chance of ending up in an autosome by moving alone or by a Y incorporation event.

Gene gains by the Y chromosome in the 400 species sample. We found (or re-discovered) several cases of gene gains by the Y chromosome, which provide useful information about Y chromosome gene composition (Table H in S1 File). Three cases seem to represent simple events: the two *kl-5* gains inside the *Drosophila* genus , and the *CG11719* gain in the ancestor of the *Zaprionus* genus. There are several cases of apparent gene gain within the *affinis* subgroup, and many within the *montium* subgroup; they all occurred after a Y chromosome incorporation and the implications of this pattern were tackled at the Results. The remaining two cases (*PRY* gene inside the *repleta* group) occurred after an opposite movement (Y to autosome/X; ref ), and may be due to a mechanism analogous to the discussed for the *pseudoobscura* and *montium* lineages: Y to autosome/X movement, with retention of the two copies of the gene, followed by loss of either copy. This retention of the two copies seem to have occurred with *kl-5* gene in the *testacea* group: the gene move to an autosome or the X at the root of the *testacea* group, but the species *D. orientacea* has both a Y-linked and an autosomal copy . However, as in the *PRY* cases we found, it is difficult to distinguish this from an independent return to the Y, after a Y to autosome movement. At any rate, this uncertainty would affect only the estimation of gene gains by the Y, which is not what we are investigating here.

The case of *D. limensis*. All tested genes are PCR-positive in females (and males) of *D. limensis*, which strongly suggests that there was a Y incorporation event. This species belongs to the *repleta* subgroup, being closely related to *D. repleta*, *D. neorepleta*, and *D. canapalpa* . The described karyotype for these species (and also *D. limensis*) has five autosomes and the XY pair ; the *D. limensis* Y chromosome has been described as "being as short as one of the small rod-shaped chromosomes" , or as "quite short" . Examination of the mitotic chromosomes of the available live strain (15084-1591.02, from SDSC) showed that males carry a very small Y chromosome (clearly the smallest chromosome), and that an unpaired chromosome of similar size and shape is present in the females (Fig F in S1 File). The extra chromosome (probably derived from the Y chromosome) was not observed in the two previous studies, which were carried out 70 years ago . These results suggest that the females we tested were XXY, perhaps due to sex-chromosome non-disjunction or some sort of translocation, and that this genetic alteration occurred in the lab, along the decades that the *D. limensis* stock has been maintained in culture. Although stock 15084-1591.02 has unknown origin, it is suspected to derive from stock 15084-1591.00, which was collected by C. Pavan in Lima, Peru, in 1945 (Carlos R. Vilela, personal communication), and presumably was used in the species description and in the cytogenetic studies. The two stocks produce identical PCR results. We searched for *D. limensis* in a sample of living flies that was collected in 2011 in the same general environment mentioned in the species description (a fruit market in Lima, Peru), but could find only *D. repleta*. We also unsuccessfully tried to identify a XX female in the *D. limensis* stock, in order to confirm that the PCR amplification of Y-linked genes in females was indeed caused by the extra-chromosome. In summary, the available evidence suggest that *D. limensis* does not represent a naturally occurring case of Y chromosome incorporation, but rather a case of XY non-disjunction or Y-translocation occurring in the laboratory. It would be very interesting to examine freshly collected lineages of *D. limensis*.

Statistical test of the re-acquisition of Y-linked genes. One reviewer correctly pointed out that the statistical test we used assumes independence of events, and that there is room for non-independence in the dataset. There are two possible sources of non-independence: (i) Many changes affect more then one closely related species, and most likely were not independent; (ii) in species that have two or more Y-linked genes, all genes might have moved in a single event. We addressed these by using a robust and conservative approach.

Regarding the first problem (non-independence of species), we considered "one gene that moved in any point in the phylogeny" as one event, irrespective of how many species were affected. This effectivelly remove the first problem. We should note that we are being conservative because simple inspection of the phylogeny (Fig 3) shows that several genes would had been independently re-acquired two or more times. For example, there would be two re-acquisitions in *kl-5* (*D. kanapiae*/*D. parvula* and *D. nikananu*), two in *Ppr-Y* (*D. kikkawai*/*D. bocki* and *D. kanapiae*), and so forth*.*  This conservative approach additionally protects the test from non-independence arising from uncertainties in the phylogeny.

The second potential problem is that if two or more genes moved at the same time they should had been counted as one event (*i.e.*, non-independence of the genes). A particularly clear case is provided by the *kl-2* and *WDY* genes in the *kikkawai* clade (Fig 3). However, these two genes also moved alone in other parts of the phylogeny (*kl-2*: *D. constricta*; *WDY*: *D. kanapiae*), so they certainly must be counted as two events. The same reasoning applies to all genes except *ARY* (see Fig B in S1 File, column 2), but even if we assume that the *ARY* movement was not independent (which amounts to not counting it in Table 1), the qualitative result of the test is the same (the *P* value changes from 0.011 to 0.015). To summarize, the conclusion that "the re-appearance of Y-linkage cannot be explained by a high gene gain rate by the Y chromosome, because only genes that formerly were Y-linked are affected " is robust.

Gene choice and primer design. Degenerate PCR primers were designed for the six Y-linked genes that are present in the ancestral *Drosophila* Y (*kl-2*, *kl-3*, *ORY*, *PPr-Y*, *PRY*, *JY-alpha*), plus the *kl-5*, *WDY* and *CG11719* genes . These nine genes were chosen because they are informative (*i.e.*, Y-linked in a large number of species), and allowed the design of reliable degenerate primers. In species in which we suspected that the whole Y chromosome was incorporated into an autosome, additional genes (that do not yield reliable degenerate primers) were tested for Y-linkage with normal PCR primers designed using a closely related sequenced species (if available). This approach was used with the *Pp1-Y1*, *Pp1-Y2* and *ARY* genes in the *montium* subgroup (designed with the *D. kikkawai* genome sequence; ), and the *ARY* gene in the *obscura* group (designed with the *D. pseudoobscura* genome sequence; ). The above set of 12 genes was identified in the Y chromosomes of either *D. melanogaster* or *D. virilis* . Finally, as shown in Table 1 and Table E in S1 File, in a small set of species we used degenerate primers for two genes recently identified in the Y chromosome of *D. willistoni* (*CG18155* and *CG14339*; A. Bernardo Carvalho and Joao Ricchio, unpublished). The full list of primers, and the PCR conditions, are in Table J and Table K in S1 File, respectively.

Degenerate primers were designed using the Codehop method , targeting protein regions that are conserved among the 12 sequenced *Drosophila* species; when the orthologs are found, we included outgroups such as *Glossina*, *Anopheles* and *Aedes* . Y-linked genes frequently have autosomal paralogs; in order to avoid cross-amplification great care was taken to avoid protein regions that are conserved with these paralogs. Also, introns in Y-linked genes tend to be very large (sometimes gigantic; so we never attempted to cross exon boundaries. Degenerate PCR can be tricky , but we achieved a very high success rate: for nearly all genes the degenerate PCRs worked in more than 90% of the species (Table B in S1 File). Besides careful primer design, two procedures usually recommended in the literature were critical to this high success rate: use of hot-start Taq (we used Thermo Fisher’s Amplitaq® Gold DNA Polymerase cat #N8080247 and Promega GoTaq® Hot-Start Polymerase cat #M5005) and design of several (two or three) forward and reverse primers, so we can test up to nine primer combinations; a preliminary test with two PCR protocols, (both with Touchdown; ref ) usually identify a few primer combinations that reliably amplify the target gene in two distantly related *Drosophila* species (*e.g.*, *D. melanogaster* and *D. virilis*). The best performing primer pairs were then used in the whole sample of species. Table J and Table K in S1 File list the primers and PCR conditions. In our hands reliable amplification of PCR products up to ~1.8 kb are routinely obtained. Besides the critical procedures described above, we made some modifications in the Codehop method that seemed to improve the success rate: (i) when possible, the degenerated core region ("core") was enlarged from 11 bp to up to 17bp; (ii) since Y-linked (and other heterochromatic genes) tend to use AT-rich codons , the clamp region of the Codehop primers was designed using a codon table derived from *Drosophila* Y-linked genes; (iii) when possible, we included a non-degenerated stretch of at least 5 bp at the 3' end of the primers (*i.e.*, we targeted a conserved region including a methionine or a tryptophan). PCRs were performed in ABI Veriti (Thermo Fisher cat # 4375786), or in Robocycler thermocyclers (Stratagene cat # 400880).

Confirmation of identity of PCR products. Spurious PCR amplification can lead to errors in linkage ascertainment (namely, a Y-linked gene be considered autosomal / X-linked). Checking the size of the PCR product in the agarose gel largely prevent this error, and in important cases (*e.g.*, when a gene movement was inferred) we confirmed the result by sequencing the PCR product in females or in both sexes, as follows. The desired PCR band was excised from the gel and its DNA was extracted with GFX™ PCR DNA and Gel Band Purification Kit (GE Healthcare, cat #28-9034-70), according to the instructions of the manufacturer. Since degenerate primers perform poorly as sequencing primers, we submitted the extracted PCR products to second round of PCR, now using a related, but not degenerated PCR primers ("nd primers"; Table J in S1 File). This second PCR effectively replaces the degenerated sequences at the tails of the PCR products by a non-degenerated sequence, suitable for sequencing. The same nd primers were then used in the sequencing. To avoid UV-induced DNA damage, we used SYBR-Safe and a blue-light transilluminator (Thermo Fisher cat #S33102 and S37102) instead of ethidium bromide and the regular UV transilluminator while excising the bands from the gel. The above approach is more convenient than cloning of PCR products, when one has a large number of samples. All sequencing was performed at Macrogen (Korea).

# Supporting Figures

| A | **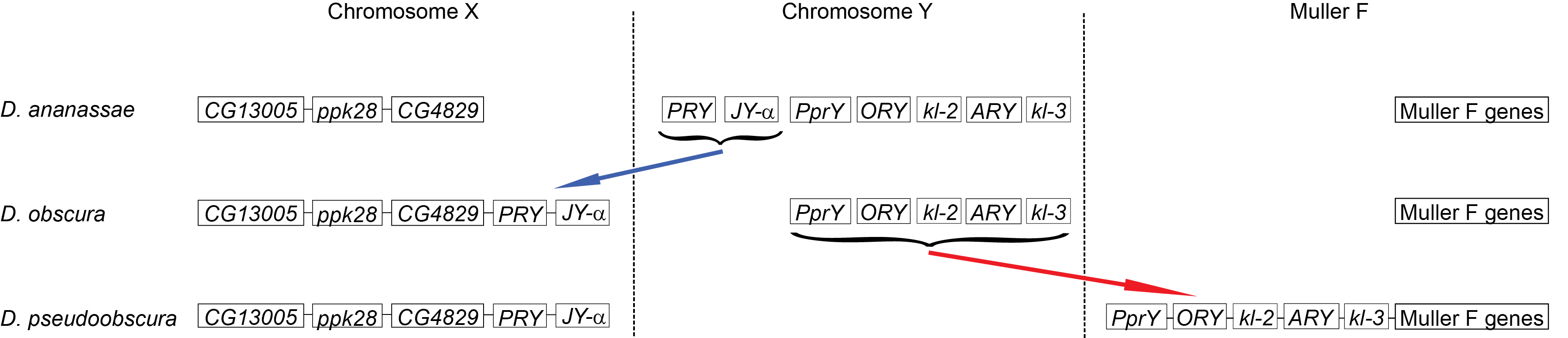** |
| --- | --- |
| B | **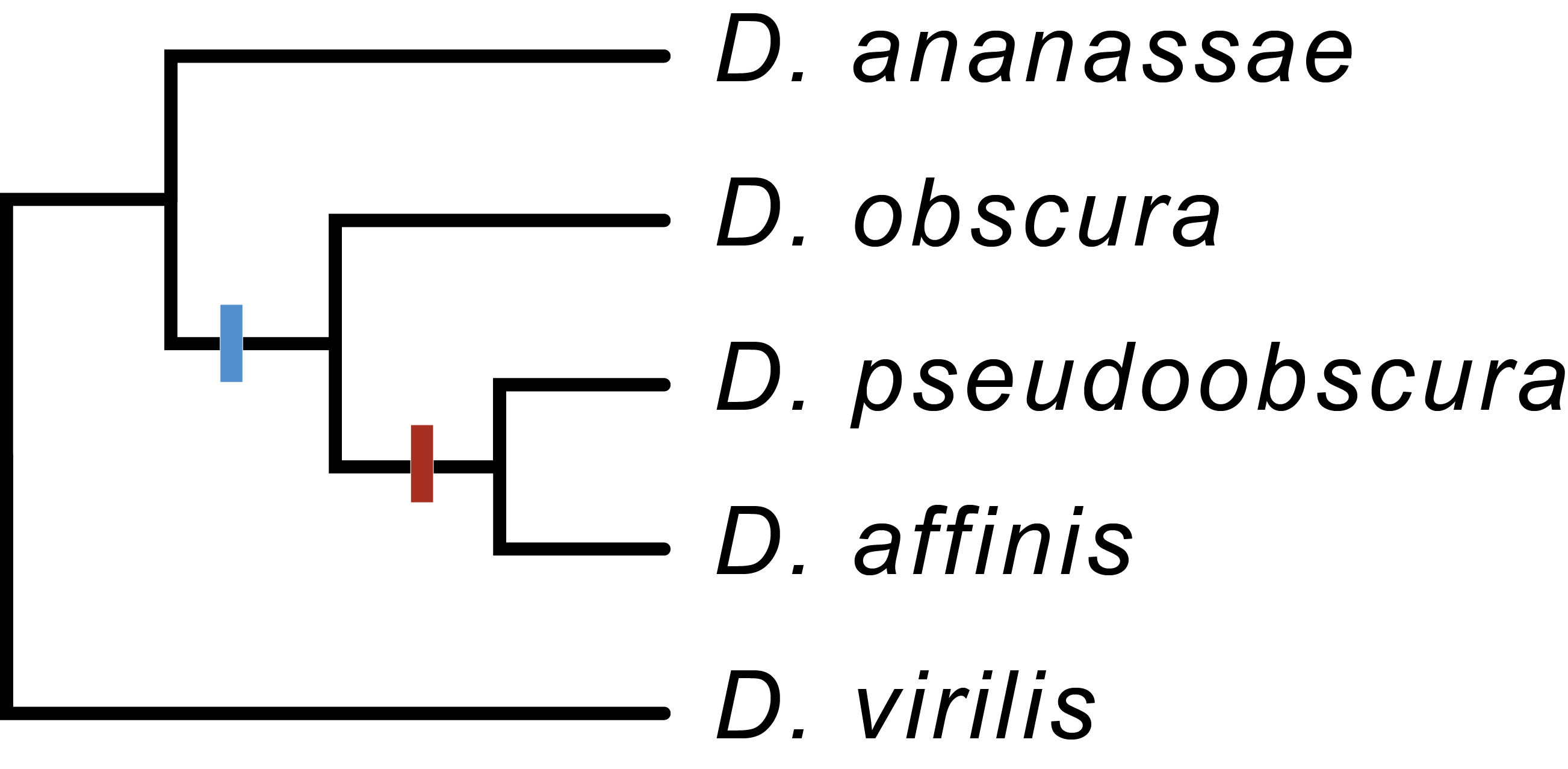** |

**Fig A. *PRY* and *JY-alpha* were lost from the Y in a single event in the ancestor of the *obscura* group.** Panel A: Synteny analysis. In most *Sophophora* species (represented here by *D. ananassae*) the *PRY*, *JY-alpha , kl-2*, *kl-3*, *PPr-Y*, *ORY* and *ARY* genes are Y-linked. In all species of the *obscura* group (represented by *D. obscura* and *D. pseudoobscura*) the *PRY* and *JY-alpha* are located on the X-chromosome, side-by-side in a conserved position; this shows that they moved to the X in a single event (blue arrow), which occurred in the ancestor of the *obscura* group, after it diverged from the *melanogaster* group and before the split between the *obscura* and *pseudoobscura* subgroup (18 to 55 Myr ago). The event of Y incorporation (red arrow), which affected only the *affinis* and *pseudoobscura* subgroups, is more recent (12.7 to 20.8 Myr ago) and placed all Y-linked genes into the Muller F chromosome. The three species were chosen because they had been sequenced (so it is possible to observe synteny), but the PCR data from the other *Sophophora* species is fully compatible with this scenario (Table A in S1 File). Note that gene order inside the Y chromosome is unknown due to assembly fragmentation. Panel B: phylogenetic interpretation, showing the time of occurrence of the *PRY/JY-alpha* loss by the Y chromosome (blue rectangle) and the Y incorporation (red rectangle). The *D. virilis* configuration is nearly identical to *D. ananassae*, which shows that this is the ancestral state. The accession numbers for the *JY-alpha* and *PRY* orthologs of *D. obscura* are XM_022353228 and XM_022353227, and of *D. pseudoobscura* are XM_002133744 and GU937417. Data from references .


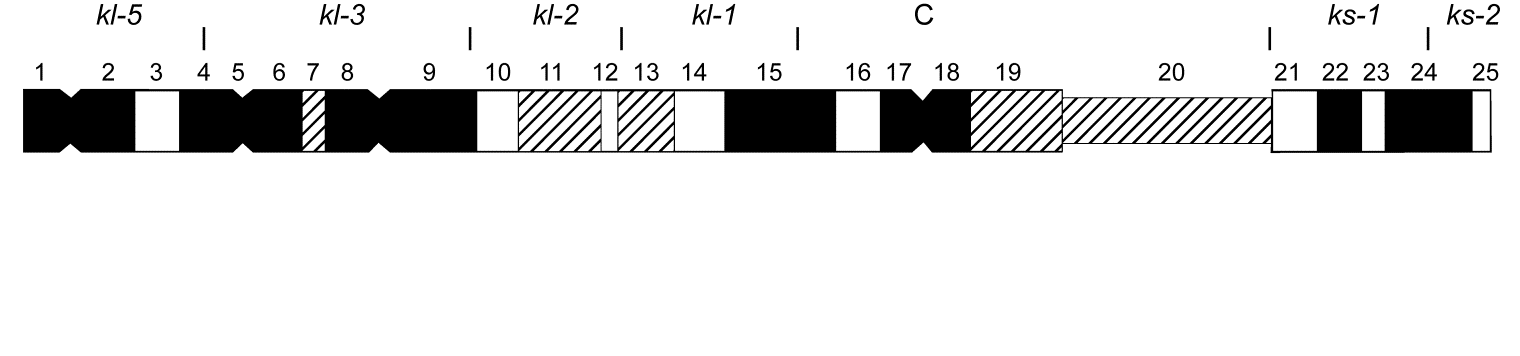


|  |  |  | | | | | | |
| --- | --- | --- | --- | --- | --- | --- | --- | --- |
| Gene | Changes in linkage | Gene location | | | | | | |
| *kl-5* | 2 |  |  |  |  |  |  |  |
| *kl-3* | 0 |  |  |  |  |  |  |  |
| *kl-2* | 3 |  |  |  |  |  |  |  |
| *WDY* | 2 |  |  |  |  |  |  |  |
| *ORY* | 1 |  |  |  |  |  |  |  |
| *Ppr-Y* | 2 |  |  |  |  |  |  |  |
| *ARY* | 1 |  |  |  |  |  |  |  |
| *PRY* | 3 |  |  |  |  |  |  |  |
| *Pp1-Y1* | 0 |  |  |  |  |  |  |  |
| *Pp1-Y2* | 2 |  |  |  |  |  |  |  |

**Fig B. Relationship between gene locations (in *D. melanogaster*) and number of gene movements in *montium* species .** Top: cytogenetic map of the *D. melanogaster* Y chromosome, used here as a proxy for the *montium* species (no data is available for them). The regions essential for male fertility (*kl-5* to *ks-2*), the translocation breakpoints used to define them (small vertical lines), the heterochromatic bands (h1 to h25), and the centromere (labeled as "C") are indicated. The *rDNA* locus is located on band h20. Data from references . Bottom: The table shows the molecularly defined genes (only those studied in this work were included). The number of independent changes in linkage in *montium* species was obtained from Fig 3, and gene locations from references . Note that there seems to be no association between cytogenetic position and changes in linkage: for example, the only genes with zero changes in linkage (*kl-3* and *Pp1-Y1*) are located in the same regions of genes with the highest number of changes (*kl-5* and *Ppr-Y*, respectively).

Fig C. Divergence times in the *montium* subgroup. In *D. nikananu* several genes (*kl-5*, *PRY*, *ORY* and *ARY*) are present only in the Y chromosome, whereas the same genes are present in females of *D. diplacantha* (and hence there is an autosomal or X-linked copy; Fig 3). The duplicated Y hypothesis

implies that the A/X and Y-linked copies of these four genes were maintained since the Y incorporation event (at least 19 Myr ago) until the divergence between *D. nikananu* and *D. diplacantha* (5.9 Myr ago); the A/X copy would had degenerated only in *D. diplacantha*. Phylogeny based on *Amyrel* sequences and divergence times estimated with the RelTime method .


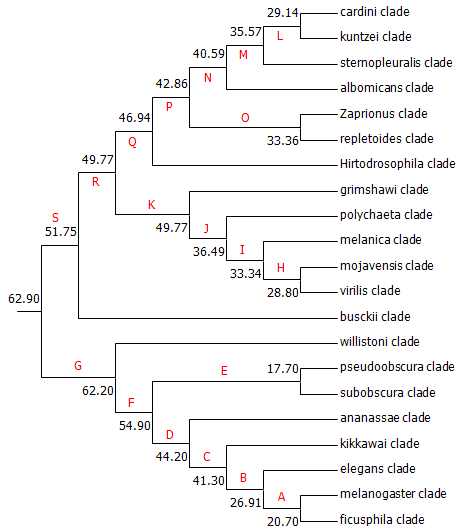


**Fig D. Backbone tree used to estimate total tree length of the 400 species.** Divergence times were taken from ref or, when not available there, were estimated with the RelTime method using *Amyrel* sequences. Numbers are the node age, and precede the corresponding node. Internal branch names are shown in red, and match Supporting Dataset_S1.xls . Each terminal branch actually represents a set of species which total branch length was estimated using the PASTIS or Yule methods (SI section "Estimation of the length of the 400 species tree"); the total length of the tree was obtained by adding the length of these terminal branches to the internal branches.


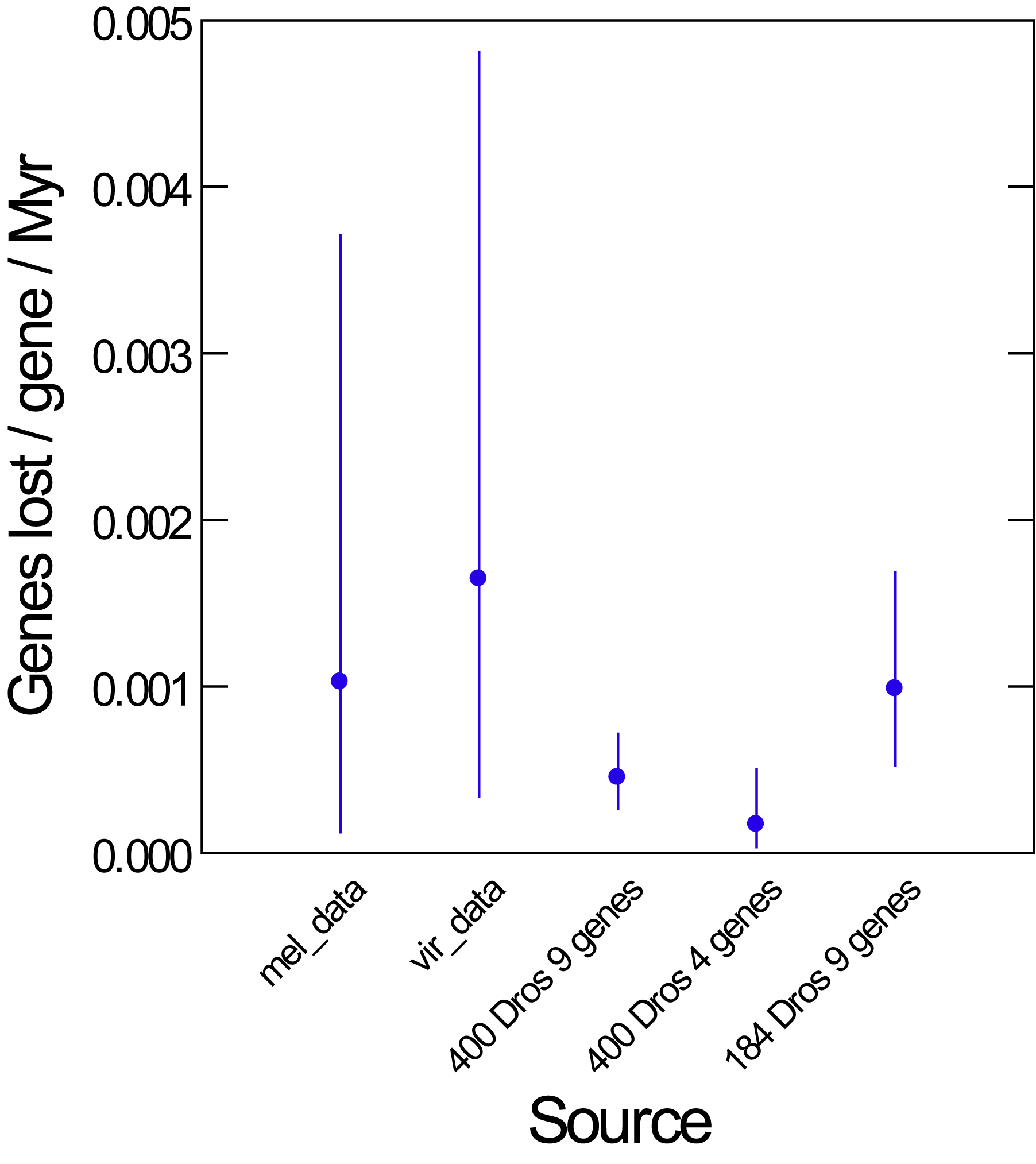


**Fig E. Comparison of estimates of gene loss rate of the Y chromosome.** The first two estimates used only the 12 sequenced species and all known Y-linked genes, and came from references . The last three estimates came from the present work, using all 400 species and the 9 sampled genes ("400 Dros 9 genes"), the 400 species and the subset of four high-confidence genes ("400 Dros 4 genes"), or the 184 species with *Amyrel* sequences and the 9 genes ("184 Dros 9 genes"). See SI section "Estimation of the gene loss rate of the Y chromosome" for details. Bars shows 95% confidence intervals.

| Male larva | Female larva |
| --- | --- |
| 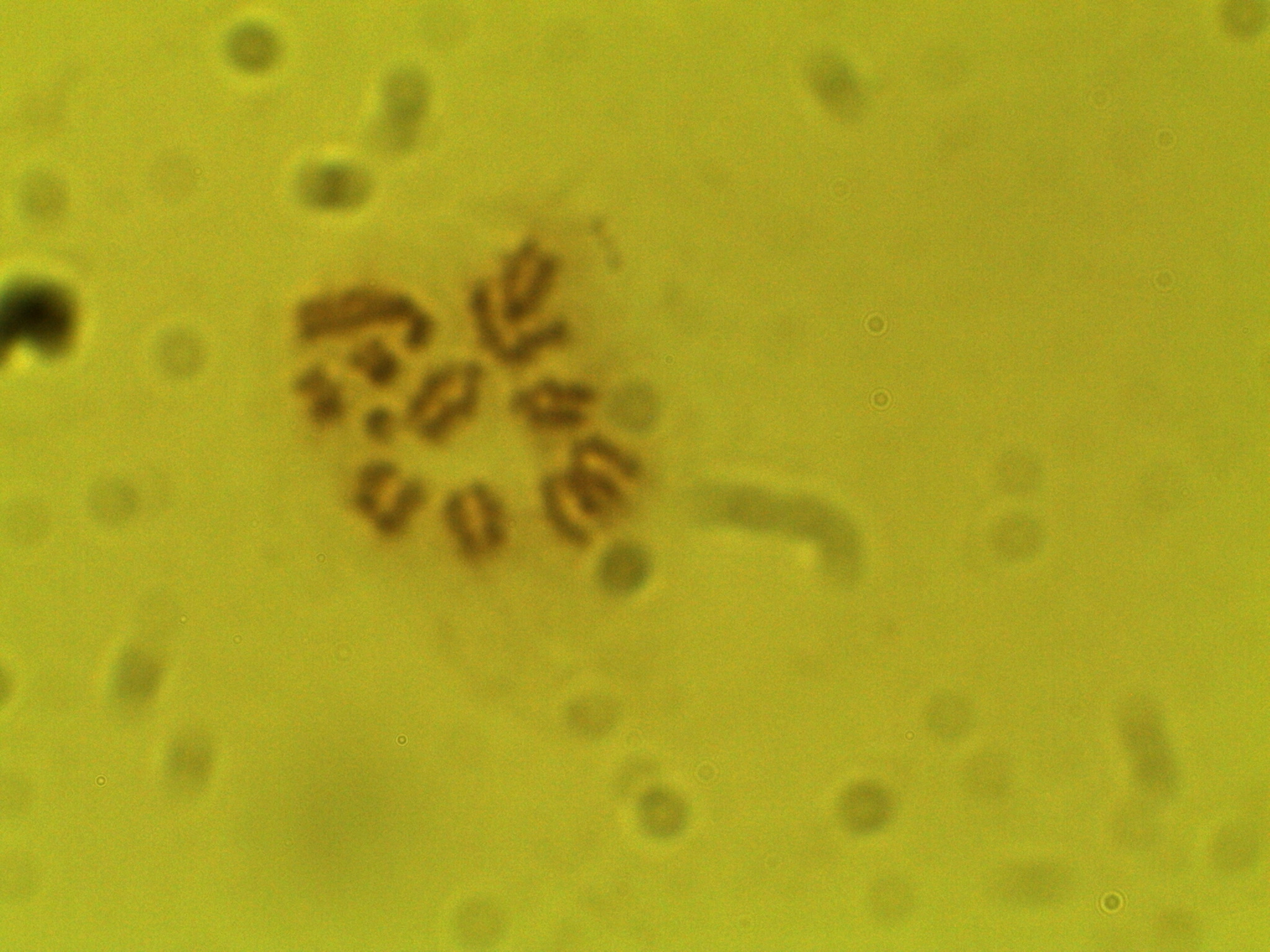 | 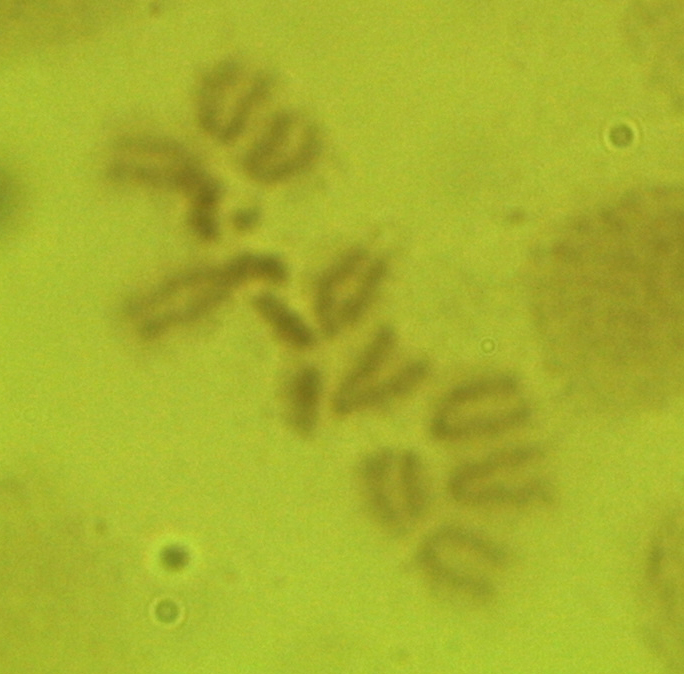 |

**Fig F. Mitotic chromosomes of *D. limensis***. Left: male larva. Note the small chromosome (white arrow) which seems to be the Y. Right: Females have a small unpaired chromosome (white arrow). This unpaired chromosome was not mentioned in the two previous cytogenetic studies of this species, which were performed 70 years ago . Hence it presumably is a mutation that occurred along the decades the stock has been kept in culture. Larvae from strain 15084-1591.02 (National *Drosophila* Stock Center).

.

# Supporting Table**s**

Table A. 400 species of *Drosophila* and related genera: sample origin, and Y-linkage results.

| **Specie name** | **group** | **subgroup** | **Sample Origin** | ***CG11719*** | ***JYα*** | ***kl-2*** | ***kl-3*** | ***kl-5*** | ***ORY*** | ***Ppr-Y*** | ***PRY*** | ***WDY*** | ***Amyrel* Seq** | **PASTIS clade** |
| --- | --- | --- | --- | --- | --- | --- | --- | --- | --- | --- | --- | --- | --- | --- |
| *Drosophila asahinai* | *melanogaster* | *montium* | DSEU (E-12501 AM2K-1) | MF | MF | MF | MF | MF | MF | MF | MF | MF | AF250051 | *kikkawai* |
| *Drosophila auraria* | *melanogaster* | *montium* | DSSC (14028-0471.00) | MF | MF | MF | MF | MF | MF | MF | MF | MF | U96163 | *kikkawai* |
| *Drosophila baimaii* | *melanogaster* | *montium* | DSSC (14028-0481.00) | MF | MF | MF | MF | MF | MF | MF | M | MF | MH707319 | *kikkawai* |
| *Drosophila barbarae* | *melanogaster* | *montium* | DSSC (14028-0491.01) | MF | MF | M | MF | MF | MF | MF | MF | M | AF250053 | *kikkawai* |
| *Drosophila biauraria* | *melanogaster* | *montium* | DSSC (14028-0501.00) | MF | MF | MF | MF | MF | MF | MF | MF | MF | AF136932 | *kikkawai* |
| *Drosophila bicornuta* | *melanogaster* | *montium* | DSSC (14028-0511.00) | MF | MF | M | MF | MF | MF | MF | MF | M | AF136933 | *kikkawai* |
| *Drosophila birchii* | *melanogaster* | *montium* | DSSC (14028-0521.01) | MF | MF | M | MF | MF | MF | MF | MF | M | MH707320 | *kikkawai* |
| *Drosophila bocki* | *melanogaster* | *montium* | Kopp | MF | MF | M | MF | MF | MF | M | M | M | MH707309 | *kikkawai* |
| *Drosophila bocqueti* | *melanogaster* | *montium* | DSSC (14028-0771.00) | MF | MF | M | MF | MF | MF | MF | MF | M | AF049092 | *kikkawai* |
| *Drosophila burlai* | *melanogaster* | *montium* | DSSC (14028-0781.00); Kopp | MF | MF | M | MF | MF | MF | MF | MF | M | AF250059 | *kikkawai* |
| *Drosophila bunnanda* | *melanogaster* | *montium* | DSSC (14028-0781.00) | MF | MF | M | MF | MF | MF | MF | MF | M | MH707323 | *kikkawai* |
| *Drosophila chauvacae* | *melanogaster* | *montium* | DSSC (14028-0761.00) | MF | MF | M | MF | MF | MF | MF | ? | M | AF250056 | *kikkawai* |
| *Drosophila constricta* | *melanogaster* | *montium* | Kopp | MF | MF | M | MF | MF | MF | MF | MF | MF | MH707310 | *kikkawai* |
| *Drosophila diplacantha* | *melanogaster* | *montium* | DSSC (14028-0586.00) | MF | MF | M | MF | MF | MF | MF | MF | M | AF251142 | *kikkawai* |
| *Drosophila greeni* | *melanogaster* | *montium* | DSSC (14028-0712.00) | MF | MF | M | MF | MF | MF | MF | MF | M | AF462602 | *kikkawai* |
| *Drosophila jambulina* | *melanogaster* | *montium* | DSSC (14028-0671.01) | MF | MF | M | MF | MF | MF | MF | MF | M | MH707322 | *kikkawai* |
| *Drosophila kanapiae* | *melanogaster* | *montium* | DSSC (14028-0541.00) | MF | MF | MF | MF | M | MF | M | M | M | MH707311 | *kikkawai* |
| *Drosophila kikkawai* | *melanogaster* | *montium* | DSSC (14028-0561.14) | MF | MF | M | MF | MF | MF | M | M | M | U96156 | *kikkawai* |
| *Drosophila lacteicornis* | *melanogaster* | *montium* | DSSC (14028-0571.00) | MF | MF | MF | MF | MF | MF | MF | MF | MF | MH707312 | *kikkawai* |
| *Drosophila leontia* | *melanogaster* | *montium* | David; Kopp | MF | MF | M | MF | MF | MF | MF | M | M | AF250058 | *kikkawai* |
| *Drosophila lini* | *melanogaster* | *montium* | DSSC (14028-0581.00) | MF | MF | M | MF | MF | MF | MF | MF | M | AF039559 | *kikkawai* |
| *Drosophila malagassya* | *melanogaster* | *montium* | David | MF | MF | M | MF | MF | MF | MF | MF | M | AF250057 | *kikkawai* |
| *Drosophila mayri* | *melanogaster* | *montium* | DSSC (14028-0591.00) | MF | MF | M | MF | MF | MF | MF | MF | M | MH707313 | *kikkawai* |
| *Drosophila nikananu* | *melanogaster* | *montium* | DSSC (14028-0601.00, 14028-0601.01) | MF | MF | M | MF | M | M | MF | M | M | AF251136 | *kikkawai* |
| *Drosophila orosa* | *melanogaster* | *montium* | DSSC (14028-0611.00) | MF | MF | M | MF | MF | MF | MF | MF | M | MH707314 | *kikkawai* |
| *Drosophila parvula* | *melanogaster* | *montium* | DSSC (14028-0621.00) | MF | MF | M | MF | M | MF | MF | M | MF | MH707315 | *kikkawai* |
| *Drosophila pectinifera* | *melanogaster* | *montium* | Kopp | MF | MF | MF | MF | MF | MF | MF | MF | MF | MH707321 | *kikkawai* |
| *Drosophila punjabiensis* | *melanogaster* | *montium* | DSSC (14028-0641.00, 14028-0531.00) | MF | M | M | MF | MF | MF | MF | MF | M | U96165 | *kikkawai* |
| *Drosophila rufa* | *melanogaster* | *montium* | DSSC (14028-0661.00) | MF | MF | MF | MF | MF | MF | MF | MF | MF | AF136935 | *kikkawai* |
| *Drosophila seguyi* | *melanogaster* | *montium* | DSSC (14028 0671.02) | MF | MF | M | MF | MF | MF | MF | MF | M | MH707324 + MH707325 | *kikkawai* |
| *Drosophila serrata* | *melanogaster* | *montium* | DSSC (14028-0681.00) | MF | MF | M | MF | MF | MF | MF | MF | M | AF069756 | *kikkawai* |
| *Drosophila subauraria* | *melanogaster* | *montium* | DSEU (E-14901 ONM29) | MF | MF | MF | MF | MF | MF | MF | MF | MF | MH707317 | *kikkawai* |
| *Drosophila tani* | *melanogaster* | *montium* | DSSC (14020-0011.01) | MF | MF | MF | MF | MF | MF | MF | MF | MF | MH707318 | *kikkawai* |
| *Drosophila triauraria* | *melanogaster* | *montium* | DSSC (14028-0651.00, 14028-0691.00) | MF | MF | MF | MF | MF | MF | MF | MF | MF | AF251141 | *kikkawai* |
| *Drosophila truncata* | *melanogaster* | *montium* | Kopp | MF | MF | M | MF | MF | MF | MF | MF | M | MH707316 | *kikkawai* |
| *Drosophila tsacasi* | *melanogaster* | *montium* | DSSC (14028-0701.00) | MF | MF | M | MF | MF | MF | MF | MF | M | AF251134 | *kikkawai* |
| *Drosophila vulcana* | *melanogaster* | *montium* | DSSC (14028-0711.00) | MF | MF | M | MF | MF | MF | MF | MF | M | AF251132 | *kikkawai* |
| *Drosophila watanabei* | *melanogaster* | *montium* | DSSC (14028 0531.02) | MF | M | M | MF | MF | MF | MF | MF | M |  | *kikkawai* |
| *Drosophila nsp. 1* | *melanogaster* | *montium* | David | MF | MF | M | MF | MF | MF | MF | MF | M |  | *kikkawai* |
| *Drosophila nsp. 2* | *melanogaster* | *montium* | David | MF | MF | M | MF | MF | MF | MF | MF | M |  | *kikkawai* |
| *Drosophila ananassae* | *melanogaster* | *ananassae* | DSSC (14024-0371.03, 14024-0371.13) | MF | M | M | M | M | M | M | M | M | AF024691 | *ananassae* |
| *Drosophila atripex* | *melanogaster* | *ananassae* | Kopp | MF | M | M | M | M | M | M | M | M | U96154 | *ananassae* |
| *Drosophila bipectinata* | *melanogaster* | *ananassae* | DSSC (14024-0381.00) | MF | M | M | M | M | M | M | ? | M | AF136936 | *ananassae* |
| *Drosophila ercepeae* | *melanogaster* | *ananassae* | Kopp | MF | M | M | M | M | M | M | M | M | U96155 | *ananassae* |
| *Drosophila malerkotliana malerkotliana* | *melanogaster* | *ananassae* | DSSC (14024-0391.00) | MF | M | M | M | M | M | M | ? | M | AY733053 | *ananassae* |
| *Drosophila malerkotliana pallens* | *melanogaster* | *ananassae* | DSSC (14024-0392.00) | MF | M | M | M | M | M | M | ? | M | AY733054 | *ananassae* |
| *Drosophila merina* | *melanogaster* | *ananassae* | Kopp | MF | M | M | M | M | M | M | M | M | AY733057 | *ananassae* |
| *Drosophila monieri* | *melanogaster* | *ananassae* | Kopp | MF | M | M | M | M | M | M | M | M | AF250052 | *ananassae* |
| *Drosophila ochrogaster* | *melanogaster* | *ananassae* | Kopp | MF | M | M | M | M | M | M | M | M | AY736485 | *ananassae* |
| *Drosophila pallidosa* | *melanogaster* | *ananassae* | DSSC (14024-0433.00) | MF | M | M | M | M | M | M | M | ? | AF136931 | *ananassae* |
| *Drosophila parabipectinata* | *melanogaster* | *ananassae* | DSSC (14024-0401.00) | MF | M | M | M | M | M | M | ? | M | AY736489 | *ananassae* |
| *Drosophila phaeopleura* | *melanogaster* | *ananassae* | DSSC (14024-0434.00) | MF | M | M | M | M | M | M | M | ? | AY736491 | *ananassae* |
| *Drosophila pseudoananassae nigrens* | *melanogaster* | *ananassae* | DSSC (14024-0411.00) | MF | M | M | M | M | M | M | M | M | AY736497 | *ananassae* |
| *Drosophila pseudoananassae pseudoananassae* | *melanogaster* | *ananassae* | DSSC (14024-0421.00) | MF | M | M | M | M | M | M | M | M | AY736498 | *ananassae* |
| *Drosophila vallismaia* | *melanogaster* | *ananassae* | Kopp | MF | M | M | M | M | M | M | M | M | AY744446 | *ananassae* |
| *Drosophila varians* | *melanogaster* | *ananassae* | Kopp | MF | M | M | M | M | M | M | M | M | AF136937 | *ananassae* |
| *Drosophila biarmipes* | *melanogaster* | *suzukii* | DSSC (14023-0361.00); David | MF | MF | M | M | M | M | M | M | M | AF462597 | *melanogaster* |
| *Drosophila elegans* | *melanogaster* | *elegans* | DSSC (14027-0461.00); David | MF | M | M | M | M | M | M | M | M | AF136930 | *elegans* |
| *Drosophila erecta* | *melanogaster* | *melanogaster* | DSSC (14021-0224.00) | MF | MF | M | M | M | M | M | M | M | AF039562 | *melanogaster* |
| *Drosophila eugracilis* | *melanogaster* | *eugracilis* | DSSC (14026-0451.02) | MF | MF | M | M | M | M | M | M | M | AF250055 | *melanogaster* |
| *Drosophila ficusphila* | *melanogaster* | *ficusphila* | DSSC (14025-0441.00) | MF | M | M | M | M | M | M | M | M | AF462600 | *ficusphila* |
| *Drosophila fuyamai* | *melanogaster* | *rhopaloa* | DSSC (14029-0011.00) | MF | M | M | M | M | M | M | M | M |  | *elegans* |
| *Drosophila lamottei* | *dentissima* | *-* | David | MF | M | M | M | M | M | M | M | M |  | *elegans* |
| *Drosophila lucipennis* | *melanogaster* | *suzukii* | DSSC (14023-0331.00) | MF | M | M | M | M | M | M | M | M | AF251138 | *elegans* |
| *Drosophila lutescens* | *melanogaster* | *takahashii* | DSSC (14022-0271.00) | MF | MF | M | M | M | M | M | M | M | AF491637 | *melanogaster* |
| *Drosophila mauritiana* | *melanogaster* | *melanogaster* | Clark | MF | MF | M | M | M | M | M | M | M | U96157 | *melanogaster* |
| *Drosophila melanogaster* | *melanogaster* | *melanogaster* | DSSC (14021-0231.36); Clark | MF | MF | M | M | M | M | M | M | M | AF022713 | *melanogaster* |
| *Drosophila mimetica* | *melanogaster* | *suzukii* | DSSC (14023-0381.00) | MF | MF | M | M | M | M | M | M | M | AY733058 | *melanogaster* |
| *Drosophila nepalensis* | *melanogaster* | *takahashii* | David | MF | MF | M | M | M | M | M | M | M |  | *melanogaster* |
| *Drosophila orena* | *melanogaster* | *melanogaster* | DSSC (14021-0245.01) | MF | MF | M | M | M | M | M | M | ? | U96158 | *melanogaster* |
| *Drosophila paralutea* | *melanogaster* | *takahashii* | DSSC (14022-0281.00) | MF | MF | M | M | M | M | M | M | M |  | *melanogaster* |
| *Drosophila prolongata* | *melanogaster* | *rhopaloa* | David | MF | M | M | M | M | M | ? | M | M |  | *elegans* |
| *Drosophila prostipennis* | *melanogaster* | *takahashii* | DSSC (14022-0291.00) | MF | MF | M | M | M | M | M | M | M |  | *melanogaster* |
| *Drosophila pseudotakahashii* | *melanogaster* | *takahashii* | DSSC (14022-0301.01) | MF | MF | M | M | M | M | M | M | M | AY736499 | *melanogaster* |
| *Drosophila rhopaloa* | *melanogaster* | *rhopaloa* | DSSC (14029-0021.01) | MF | M | M | M | M | M | ? | M | M | FlyBase | *elegans* |
| *Drosophila santomea* | *melanogaster* | *melanogaster* | DSSC (14021-0271.00) | MF | MF | M | M | M | M | M | M | M | AY736503 | *melanogaster* |
| *Drosophila sechellia* | *melanogaster* | *melanogaster* | DSSC (14021-0248.25 Robertson 3C) | MF | MF | M | M | M | M | M | M | M | AF039558 | *melanogaster* |
| *Drosophila simulans* | *melanogaster* | *melanogaster* | Clark | MF | MF | M | M | M | M | M | M | M | U96159 | *melanogaster* |
| *Drosophila subpulchrella* | *melanogaster* | *suzukii* | DSEU (E-15201) | MF | MF | M | M | M | M | M | M | M |  | *melanogaster* |
| *Drosophila suzukii* | *melanogaster* | *suzukii* | Vilela | MF | MF | M | M | M | M | M | M | M | DS10_00003293 | *melanogaster* |
| *Drosophila takahashii* | *melanogaster* | *takahashii* | DSSC (14022-0311.00) | MF | MF | M | M | M | M | M | M | M | U96161 | *melanogaster* |
| *Drosophila teissieri* | *melanogaster* | *melanogaster* | DSSC (14021-0257.00) | MF | MF | M | M | M | M | M | M | M | AF039557.2 | *melanogaster* |
| *Drosophila yakuba* | *melanogaster* | *melanogaster* | DSSC (14021-0261.01); Clark | MF | MF | M | M | M | M | M | M | M | AF039561 | *melanogaster* |
| *Drosophila ambigua* | *obscura* | *obscura* | Cariou | MF | MF | M | M | MF | M | M | MF | MF | AF306712 | *obscura* |
| *Drosophila bifasciata* | *obscura* | *obscura* | DSSC (14012-0181.01) | MF | ? | M | M | MF | M | M | ? | MF | AF251135 | *obscura* |
| *Drosophila guanche* | *obscura* | *obscura* | DSSC (14011-0095.00) | MF | MF | M | M | MF | M | M | ? | MF | AF306711 | *obscura* |
| *Drosophila imaii* | *obscura* | *obscura* | DSEU (E-13801) | MF | MF | M | M | MF | M | M | ? | MF | AF251133 | *obscura* |
| *Drosophila microlabis* | *obscura* | *obscura* | David | MF | MF | M | M | MF | M | M | ? | MF | AF150684 | *obscura* |
| *Drosophila obscura* | *obscura* | *obscura* | DSSC (14011-0151.00) | MF | MF | M | M | MF | M | M | MF | MF | AF306713 | *obscura* |
| *Drosophila subobscura* | *obscura* | *obscura* | Bachtrog | MF | MF | M | M | MF | M | M | ? | MF | U79724 | *obscura* |
| *Drosophila subsilvestris* | *obscura* | *obscura* | David | MF | MF | M | M | MF | M | M | ? | MF | AF306714 | *obscura* |
| *Drosophila tristis* | *obscura* | *obscura* | DSSC (14011-0141.00) | MF | MF | M | M | MF | M | M | MF | MF | AF150683 | *obscura* |
| *Drosophila tsukubaensis* | *obscura* | *obscura* | DSEU (E-15501) | MF | MF | M | M | MF | M | ? | MF | MF | AF150681 | *obscura* |
| *Drosophila lowei* | *obscura* | *pseudoobscura* | Noor | MF | MF | MF | MF | MF | MF | MF | MF | MF |  | *pseudoobscura* |
| *Drosophila miranda* | *obscura* | *pseudoobscura* | DSSC (14011-0101.08) | MF | MF | MF | MF | MF | MF | MF | MF | MF | FlyBase | *pseudoobscura* |
| *Drosophila persimilis* | *obscura* | *pseudoobscura* | DSSC (14011-0111.49); Clark | MF | MF | MF | MF | MF | MF | MF | MF | MF | AF129823 | *pseudoobscura* |
| *Drosophila pseudoobscura bogotana* | *obscura* | *pseudoobscura* | Clark | MF | MF | MF | MF | MF | MF | MF | MF | MF | AF312739 | *pseudoobscura* |
| *Drosophila pseudoobscura pseudoobscura* | *obscura* | *pseudoobscura* | Clark | MF | MF | MF | MF | MF | MF | MF | MF | MF | U82556 | *pseudoobscura* |
| *Drosophila affinis* | *obscura* | *affinis* | DSSC (14012-0141.01); McAllister | MF | MF | MF | MF | MF | MF | MF | MF | MF | AF037353 | *pseudoobscura* |
| *Drosophila algonquin* | *obscura* | *affinis* | DSSC (14012-0161.00, 14012-0161.03); McAllister | MF | MF | M | MF | MF | MF | MF | MF | MF |  | *pseudoobscura* |
| *Drosophila athabasca* | *obscura* | *affinis* | Jaenike; McAllister | MF | MF | M | MF | MF | M | MF | MF | MF | AF306715 | *pseudoobscura* |
| *Drosophila azteca* | *obscura* | *affinis* | DSSC (14012-0171.01) | MF | MF | MF | MF | MF | MF | MF | ? | MF | AF150685 | *pseudoobscura* |
| *Drosophila helvetica* | *obscura* | *affinis* | David | MF | MF | MF | MF | MF | MF | MF | MF | MF | AF306716 | *pseudoobscura* |
| *Drosophila narragansett* | *obscura* | *affinis* | McAllister | MF | MF | MF | MF | MF | MF | MF | MF | MF |  | *pseudoobscura* |
| *Drosophila tolteca* | *obscura* | *affinis* | DSSC (14012-0201.00) | MF | MF | MF | MF | MF | MF | MF | MF | MF | AF306717 | *pseudoobscura* |
| *Drosophila capricorni* | *willistoni* | *bocainensis* | DSSC (14030-0721.00); Klaczko | MF | M | M | M | MF | M | M | M | MF |  | *willistoni* |
| *Drosophila equinoxialis* | *willistoni* | *willistoni* | DSSC (14030-0741.00, 14030-0741.02) | MF | M | M | M | MF | M | M | M | MF |  | *willistoni* |
| *Drosophila fumipennis* | *willistoni* | *bocainensis* | DSSC (14030-0751.01) | MF | M | M | M | MF | M | M | M | MF |  | *willistoni* |
| *Drosophila nebulosa* | *willistoni* | *bocainensis* | DSSC (14030-0761.00) | MF | M | M | M | MF | M | M | M | MF | AY733060 | *willistoni* |
| *Drosophila parabocainensis* | *willistoni* | *bocainensis* | Vilela | MF | M | M | M | MF | M | M | M | MF |  | *willistoni* |
| *Drosophila paulistorum* | *willistoni* | *willistoni* | DSSC (14030-0771.00) | MF | M | M | M | MF | M | M | M | MF |  | *willistoni* |
| *Drosophila sucinea* | *willistoni* | *bocainensis* | DSSC (14030-0791.00) | MF | M | M | M | MF | M | M | M | MF |  | *willistoni* |
| *Drosophila tropicalis* | *willistoni* | *willistoni* | DSSC (14030-0801.00) | MF | M | M | M | MF | M | M | M | MF | AF251140 | *willistoni* |
| *Drosophila willistoni willistoni* | *willistoni* | *willistoni* | DSSC (14030-0811.00, 14030-0811.24) | MF | M | M | M | MF | M | M | M | MF | AF039560 | *willistoni* |
| *Drosophila willistoni winge* | *willistoni* | *willistoni* | Klaczko | MF | M | M | M | MF | M | M | M | MF |  | *willistoni* |
| *Drosophila austrosaltans* | *saltans* | *saltans* | DSSC (14045-0881.00) | MF | M | M | M | MF | M | M | M | MF |  | *willistoni* |
| *Drosophila dacunhai* | *saltans* | *sturtevanti* | DSSC (14043-0854.00) | MF | M | M | M | MF | M | M | M | MF |  | *willistoni* |
| *Drosophila emarginata* | *saltans* | *elliptica* | DSSC (14042-0841.06) | MF | M | M | M | MF | M | M | ? | MF |  | *willistoni* |
| *Drosophila lusaltans* | *saltans* | *saltans* | DSSC (14045-0891.00) | MF | M | M | M | MF | M | M | M | MF |  | *willistoni* |
| *Drosophila milleri* | *saltans* | *sturtevanti* | DSSC (14043-0861.00) | MF | M | M | M | MF | M | M | M | MF |  | *willistoni* |
| *Drosophila neocordata* | *saltans* | *cordata* | DSSC (14041-0831.00) | MF | M | M | M | MF | M | M | M | MF | AY736480 | *willistoni* |
| *Drosophila prosaltans* | *saltans* | *saltans* | DSSC (14045-0901.02) | MF | M | M | M | MF | M | M | M | MF |  | *willistoni* |
| *Drosophila saltans* | *saltans* | *saltans* | DSSC (14045-0911.00) | MF | M | M | M | MF | M | M | M | MF |  | *willistoni* |
| *Drosophila sturtevanti* | *saltans* | *sturtevanti* | DSSC (14043-0871.01); Klaczko | MF | M | M | M | MF | M | M | M | MF | AY736506 | *willistoni* |
| *Drosophila subsaltans* | *saltans* | *parasaltans* | DSSC (14044-0872.00) | MF | M | M | M | MF | M | M | M | MF |  | *willistoni* |
| *Drosophila I62F9* | *saltans* | *elliptica* | Vilela | MF | M | M | M | MF | M | M | M | MF |  | *willistoni* |
| *Drosophila aldrichi* | *repleta* | *mulleri* | DSSC (15081-1251.01) | M | M | M | M | M | M | M | MF | MF |  | *mojavensis* |
| *Drosophila arizonae* | *repleta* | *mulleri* | DSSC (15081-1271.00) | M | M | M | M | M | M | M | MF | MF |  | *mojavensis* |
| *Drosophila borborema* | *repleta* | *mulleri* | DSSC (15081-1281.01) | M | M | M | M | M | M | M | MF | MF |  | *mojavensis* |
| *Drosophila buzzatii* | *repleta* | *mulleri* | DSSC (15081-1291.01) | M | M | M | M | M | M | M | MF | MF | KF632677 | *mojavensis* |
| *Drosophila hamatofila* | *repleta* | *mulleri* | DSSC (15081-1301.00) | M | M | M | M | M | M | M | MF | MF |  | *mojavensis* |
| *Drosophila hexastigma* | *repleta* | *mulleri* | DSSC (15081-1302.00) | M | M | M | M | M | M | M | MF | MF |  | *mojavensis* |
| *Drosophila huaylasi* | *repleta* | *mulleri* | DSSC (15081-1303.00) | M | M | M | M | M | M | M | MF | MF |  | *mojavensis* |
| *Drosophila koepferae* | *repleta* | *mulleri* | DSSC (15081-1305.00) | M | M | M | M | M | M | M | MF | MF |  | *mojavensis* |
| *Drosophila longicornis* | *repleta* | *mulleri* | DSSC (15081-1311.02) | M | M | M | M | M | M | M | MF | MF |  | *mojavensis* |
| *Drosophila mainlandi* | *repleta* | *mulleri* | DSSC (15081-1315.00) | M | M | M | M | M | M | M | MF | MF |  | *mojavensis* |
| *Drosophila mayaguana* | *repleta* | *mulleri* | DSSC (15081-1397.00) | M | M | M | M | M | M | M | MF | MF |  | *mojavensis* |
| *Drosophila mojavensis baja* | *repleta* | *mulleri* | Markow | M | M | M | M | M | M | M | MF | MF |  | *mojavensis* |
| *Drosophila mojavensis sonorensis* | *repleta* | *mulleri* | DSSC (15081-1351.00) | M | M | M | M | M | M | M | MF | MF |  | *mojavensis* |
| *Drosophila mojavensis wrigleyi* | *repleta* | *mulleri* | DSSC (15081-1352.22) | M | M | M | M | M | M | M | MF | MF | FlyBase | *mojavensis* |
| *Drosophila mulleri* | *repleta* | *mulleri* | DSSC (15081-1371.00) | M | M | M | M | M | M | M | MF | MF |  | *mojavensis* |
| *Drosophila navojoa* | *repleta* | *mulleri* | DSSC (15081-1374.00) | M | M | M | M | M | M | M | MF | MF |  | *mojavensis* |
| *Drosophila nigrodumosa* | *repleta* | *mulleri* | DSSC (15081-1385.00) | M | M | M | M | M | M | M | MF | MF | AY736482 | *mojavensis* |
| *Drosophila pachuca* | *repleta* | *mulleri* | DSSC (15081-1391.02) | M | M | M | M | M | M | M | MF | MF |  | *mojavensis* |
| *Drosophila parisiena* | *repleta* | *mulleri* | DSSC (15081-1392.00) | M | M | M | M | M | M | M | MF | MF |  | *mojavensis* |
| *Drosophila propachuca* | *repleta* | *mulleri* | DSSC (15081-1411.05) | M | M | M | M | M | M | M | MF | MF |  | *mojavensis* |
| *Drosophila richardsoni* | *repleta* | *mulleri* | DSSC (15081-1421.00) | M | M | M | M | M | M | M | MF | MF |  | *mojavensis* |
| *Drosophila ritae* | *repleta* | *mulleri* | DSSC (15081-1471.02) | M | M | M | M | M | M | M | MF | MF |  | *mojavensis* |
| *Drosophila serido* | *repleta* | *mulleri* | DSSC (15081-1431.01) | M | M | M | M | M | M | M | MF | MF |  | *mojavensis* |
| *Drosophila sonorae* | *repleta* | *mulleri* | DSSC (15081-1312.00) | M | M | M | M | M | M | M | MF | MF |  | *mojavensis* |
| *Drosophila spenceri* | *repleta* | *mulleri* | DSSC (15081-1441.00) | M | M | M | M | M | M | M | MF | MF |  | *mojavensis* |
| *Drosophila stalkeri* | *repleta* | *mulleri* | DSSC (15081-1451.00) | M | M | M | M | M | M | M | MF | MF |  | *mojavensis* |
| *Drosophila starmeri* | *repleta* | *mulleri* | DSSC (15081-1461.02) | M | M | M | M | M | M | M | M | MF |  | *mojavensis* |
| *Drosophila straubae* | *repleta* | *mulleri* | DSSC (15081-1462.00) | M | M | M | M | M | M | M | MF | MF |  | *mojavensis* |
| *Drosophila venezolana* | *repleta* | *mulleri* | DSSC (15081-1496.00) | M | M | M | M | M | M | M | M | MF |  | *mojavensis* |
| *Drosophila wheeleri* | *repleta* | *mulleri* | DSSC (15081-1501.10) | M | M | M | M | M | M | M | MF | MF | AY736514 | *mojavensis* |
| *Drosophila anceps* | *repleta* | *mulleri* | DSSC (15081-1261.00) | M | M | M | M | M | M | M | ? | MF |  | *mojavensis* |
| *Drosophila bifurca* | *repleta* | *hydei* | DSSC (15085-1621.00) | M | M | M | M | M | M | M | MF | MF |  | *mojavensis* |
| *Drosophila eleonorae* | *repleta* | *repleta* | Vilela | M | M | M | M | M | M | M | MF | MF |  | *mojavensis* |
| *Drosophila ellisoni* | *repleta* | *fasciola* | DSSC (15083-1551.00) | M | M | M | M | M | M | M | M | MF |  | *mojavensis* |
| *Drosophila eohydei* | *repleta* | *hydei* | DSSC (15085-1631.00) | M | M | M | M | M | M | M | MF | MF |  | *mojavensis* |
| *Drosophila eremophila* | *repleta* | *mulleri* | DSSC (15081-1292.00) | M | M | M | M | M | M | M | ? | MF |  | *mojavensis* |
| *Drosophila fulvimacula flavorepleta* | *repleta* | *repleta* | DSSC (15084-1561.00) | M | M | M | M | M | M | M | MF | MF |  | *mojavensis* |
| *Drosophila fulvimacula fulvimacula* | *repleta* | *repleta* | DSSC (15084-1571.00) | M | M | M | M | M | M | M | MF | MF |  | *mojavensis* |
| *Drosophila fulvimaculoides* | *repleta* | *repleta* | DSSC (15084-1581.00) | M | M | M | M | M | M | M | MF | MF |  | *mojavensis* |
| *Drosophila guayllabambae* | *repleta* | *hydei* | DSSC (15085-1651.00) | M | M | M | M | M | M | M | MF | MF |  | *mojavensis* |
| *Drosophila hydei* | *repleta* | *hydei* | DSSC (15085-1641.00) | M | M | M | M | M | M | M | MF | MF | AY733042 | *mojavensis* |
| *Drosophila leonis* | *repleta* | *mulleri* | DSSC (15081-1395.00) | M | M | M | M | M | M | M | ? | MF |  | *mojavensis* |
| *Drosophila limensis* | *repleta* | *repleta* | DSSC (15084-1591.00, 15084-1591.02) | MF | MF | MF | MF | MF | MF | MF | MF | MF | AY736479 | *mojavensis* |
| *Drosophila mercatorum mercatorum* | *repleta* | *mercatorum* | DSSC (15082-1521.00) | M | M | M | M | M | M | M | MF | MF |  | *mojavensis* |
| *Drosophila mercatorum pararepleta* | *repleta* | *mercatorum* | DSSC (15082-1531.00) | M | M | M | M | M | M | M | MF | MF |  | *mojavensis* |
| *Drosophila meridiana meridiana* | *repleta* | *mulleri* | DSSC (15081-1331.00) | M | M | M | M | M | M | M | MF | MF |  | *mojavensis* |
| *Drosophila meridiana rioensis* | *repleta* | *mulleri* | DSSC (15081-1341.00) | M | M | M | M | M | M | M | MF | MF |  | *mojavensis* |
| *Drosophila meridionalis* | *repleta* | *mulleri* | DSSC (15081-1344.00) | M | M | M | M | M | M | M | MF | MF |  | *mojavensis* |
| *Drosophila mettleri* | *repleta* | *mulleri* | DSSC (15081-1502.00) | M | M | M | M | M | M | M | ? | MF |  | *mojavensis* |
| *Drosophila micromettleri* | *repleta* | *mulleri* | DSSC (15081-1346.00) | M | M | M | M | M | M | M | MF | MF |  | *mojavensis* |
| *Drosophila neorepleta* | *repleta* | *repleta* | DSSC (15083-1554.00, 15084-1601.00) | M | M | M | M | M | M | M | MF | MF |  | *mojavensis* |
| *Drosophila nigricruria* | *repleta* | *mulleri* | DSSC (15081-1381.02) | M | M | M | M | M | M | M | MF | MF |  | *mojavensis* |
| *Drosophila nigrospiracula* | *repleta* | *mulleri* | DSSC (15081-1503.00) | M | M | M | M | M | M | M | MF | MF |  | *mojavensis* |
| *Drosophila paranaensis* | *repleta* | *mercatorum* | DSSC (15082-1541.00) | M | M | M | M | M | M | M | MF | MF |  | *mojavensis* |
| *Drosophila pegasa* | *repleta* | *mulleri* | DSSC (15081-1398.00) | M | M | M | M | M | M | M | MF | MF |  | *mojavensis* |
| *Drosophila peninsularis* | *repleta* | *mercatorum* | DSSC (15081-1401.00) | M | M | M | M | M | M | M | MF | MF |  | *mojavensis* |
| *Drosophila repleta* | *repleta* | *repleta* | DSSC (15084-1611.00) | M | M | M | M | M | M | M | MF | MF | AY736496 | *mojavensis* |
| *Drosophila zottii* | *repleta* | *repleta* | Vilela | M | M | M | M | ? | ? | M | ? | MF |  | *mojavensis* |
| *Drosophila I63F4* | *repleta* | *repleta* | Vilela | M | ? | M | M | M | M | M | MF | MF |  | *mojavensis* |
| *Drosophila T52W2* | *repleta* | *fasciola* | Carvalho/Vaz | M | M | M | M | M | M | M | M | MF |  | *mojavensis* |
| *Drosophila acanthoptera* | *nannoptera* | *-* | DSSC (15090-1693.00) | M | M | M | M | M | M | M | ? | MF | KF632675 | *mojavensis* |
| *Drosophila annulimana* | *annulimana* | *annulimana* | Klaczko | M | M | M | M | M | M | MF | MF | MF |  | *mojavensis* |
| *Drosophila aracataca* | *annulimana* | *annulimana* | DSSC (15040-1171.00) | M | M | M | M | M | M | M | MF | MF | AY733052 | *mojavensis* |
| *Drosophila arapuan* | *annulimana* | *arassari* | Vilela | M | M | M | M | M | M | M | M | MF |  | *mojavensis* |
| *Drosophila ararama* | *annulimana* | *gibberosa* | Vilela | M | M | M | M | M | ? | M | ? | MF |  | *mojavensis* |
| *Drosophila brncici* | *mesophragmatica* | *mesophragmatica* | Loreto/Robe | M | M | M | M | M | ? | M | ? | MF |  | *mojavensis* |
| *Drosophila bromeliae* | *bromeliae* | *-* | DSSC (15085-1682.00) | M | M | M | M | M | M | M | MF | MF | AY733049 | *mojavensis* |
| *Drosophila bromelioides* | *bromeliae* | *-* | Carvalho/Vaz | M | M | M | M | M | ? | M | MF | MF |  | *mojavensis* |
| *Drosophila calatheae* | *-* | *-* | Carvalho/Vaz | M | M | M | M | M | M | M | MF | MF |  | *mojavensis* |
| *Drosophila camargoi* | *dreyfusi* | *-* | DSSC (15060-1221.01) | M | ? | M | M | M | M | M | MF | MF | AF462598 | *mojavensis* |
| *Drosophila canalinea* | *canalinea* | *-* | DSSC (15050-1201.00) | M | ? | M | M | M | M | MF | MF | MF | KF632678 | *mojavensis* |
| *Drosophila caponei* | *caponei* | *-* | Carvalho/Vaz | M | ? | M | M | M | M | M | MF | MF |  | *mojavensis* |
| *Drosophila cestri* | *flavopilosa* | *-* | Ludwig | ? | M | M | M | M | M | M | MF | MF |  | *mojavensis* |
| *Drosophila coffeata* | *coffeata* | *-* | Carvalho/Vaz | M | M | M | M | M | M | M | MF | MF |  | *mojavensis* |
| *Drosophila dreyfusi* | *dreyfusi* | *-* | Klaczko | M | M | M | M | M | M | M | MF | MF |  | *mojavensis* |
| *Drosophila flexa* | *-* | *-* | Carvalho/Vaz | MF | M | M | M | M | M | ? | MF | MF |  | *mojavensis* |
| *Drosophila fuscolineata* | *coffeata* | *-* | Vilela | M | ? | M | M | M | M | M | MF | ? |  | *mojavensis* |
| *Drosophila gaucha* | *mesophragmatica* | *mesophragmatica* | DSSC (15070-1231.00); David | M | M | M | M | M | M | M | MF | MF |  | *mojavensis* |
| *Drosophila gasici* | *mesophragmatica* | *mesophragmatica* | Loreto/Robe | M | M | M | M | M | ? | M | M | MF |  | *mojavensis* |
| *Drosophila gibberosa* | *annulimana* | *gibberosa* | DSSC (15040-1181.00) | M | M | M | M | M | M | M | MF | MF | AY733041 | *mojavensis* |
| *Drosophila incompta* | *flavopilosa* | *nesiota* | Ludwig | M | M | M | M | M | M | ? | MF | MF |  | *mojavensis* |
| *Drosophila morelia* | *morelia* | *-* | Carvalho/Vaz | ? | M | M | ? | M | M | ? | MF | MF |  | *mojavensis* |
| *Drosophila nannoptera* | *nannoptera* | *-* | DSSC (15090-1692.00) | M | M | M | M | M | MF | M | MF | MF | KF632682 | *mojavensis* |
| *Drosophila pachea* | *nannoptera* | *-* | DSSC (15090-1698.01) | M | M | M | M | M | M | M | MF | MF | KF632683 | *mojavensis* |
| *Drosophila pavani* | *mesophragmatica* | *mesophragmatica* | DSSC (15070-1241.00) | M | M | M | M | M | M | M | MF | MF | AY736490 | *mojavensis* |
| *Drosophila pseudotalamancana* | *annulimana* | *gibberosa* | DSSC (15040-1191.01) | M | M | M | M | M | M | M | MF | MF | AY736509 | *mojavensis* |
| *Drosophila wassermani* | *nannoptera* | *-* | DSSC (15090-1697.10) | M | M | M | M | M | M | M | MF | MF | KF632686 | *mojavensis* |
| *Drosophila T10W2* | *canalinea* | *-* | Carvalho/Vaz | M | ? | M | M | M | M | ? | ? | MF |  | *mojavensis* |
| *Drosophila T18W3/4* | *xanthopallescens* | *-* | Carvalho/Vaz | M | M | M | M | M | M | M | MF | MF |  | *mojavensis* |
| *Drosophila T18W5/6* | *xanthopallescens* | *-* | Carvalho/Vaz | M | M | M | M | M | M | M | MF | MF |  | *mojavensis* |
| *Drosophila T52W1 (I4)* | *-* | *-* | Carvalho/Vaz | M | M | M | M | M | M | M | MF | MF |  | *mojavensis* |
| *Drosophila T21W2* | *mesophragmatica* | *-* | Carvalho/Vaz | MF | M | M | M | M | M | M | MF | MF |  | *mojavensis* |
| *Drosophila T53W3* | *bromeliae* | *-* | Carvalho/Vaz | M | M | M | M | M | ? | M | ? | MF |  | *mojavensis* |
| *Drosophila T58W3* | *dreyfusi* | *-* | Carvalho/Vaz | M | M | M | M | M | M | ? | MF | MF |  | *mojavensis* |
| *Drosophila americana americana* | *virilis* | *americana* | DSSC (15010-0951.00) | M | M | M | M | M | M | M | M | MF | AY736529 | *virilis* |
| *Drosophila americana texana* | *virilis* | *americana* | DSSC (15010-1041.00) | M | M | M | M | M | M | M | M | MF | AY736540 | *virilis* |
| *Drosophila borealis* | *virilis* | *-* | DSSC (15010-0961.00) | M | M | M | M | M | M | M | M | MF |  | *virilis* |
| *Drosophila canadiana* | *virilis* | *-* | DSSC (15010-1091.00) | M | M | M | M | M | M | M | M | MF |  | *virilis* |
| *Drosophila ezoana* | *virilis* | *-* | DSSC (15010-0971.00) | M | M | M | M | M | M | M | M | MF |  | *virilis* |
| *Drosophila flavomontana* | *virilis* | *-* | DSSC (15010-0981.00) | M | M | M | M | M | M | M | M | MF |  | *virilis* |
| *Drosophila kanekoi* | *virilis* | *-* | DSSC (15010-1061.00) | M | M | M | M | M | M | M | M | MF | AY736535 | *virilis* |
| *Drosophila lacicola* | *virilis* | *-* | DSSC (15010-0991.00) | M | M | M | M | M | M | M | M | MF |  | *virilis* |
| *Drosophila littoralis* | *virilis* | *-* | DSSC (15010-1001.00) | ? | M | M | M | M | M | M | M | MF | AY733045 | *virilis* |
| *Drosophila lummei* | *virilis* | *-* | DSSC (15010-1011.01) | M | M | M | M | M | M | M | M | MF | AY733046 | *virilis* |
| *Drosophila montana* | *virilis* | *montana* | DSSC (15010-1021.09) | M | M | M | M | M | M | M | M | MF |  | *virilis* |
| *Drosophila novamexicana* | *virilis* | *-* | DSSC (15010-1031.00) | M | M | M | M | M | M | M | M | MF | AY736484 | *virilis* |
| *Drosophila virilis* | *virilis* | *virilis* | DSSC (15010-1051.87, 15010-1051.00) | M | M | M | M | M | M | M | M | MF | AF136603 | *virilis* |
| *Drosophila carbonaria* | *carbonaria* | *-* | DSSC (15400-0011.00) | M | M | M | M | M | M | M | M | MF |  | *melanica* |
| *Drosophila euronotus* | *melanica* | *-* | DSSC (15030-1131.01) | M | M | M | M | M | M | M | M | MF |  | *melanica* |
| *Drosophila lacertosa* | *robusta* | *lacertosa* | DSSC (15020-1101.00) | M | M | M | M | M | M | M | M | MF |  | *melanica* |
| *Drosophila melanica* | *melanica* | *-* | DSSC (15030-1141.00) | M | M | M | M | M | M | M | M | MF | AY733056 | *melanica* |
| *Drosophila micromelanica* | *melanica* | *-* | DSSC (15030-1151.00) | M | M | M | M | M | M | M | M | MF | JF735880 | *melanica* |
| *Drosophila nigromelanica* | *melanica* | *-* | DSSC (15030-1171.00) | M | M | M | M | M | M | M | M | MF |  | *melanica* |
| *Drosophila paramelanica* | *melanica* | *-* | DSSC (15030-1161.00) | M | M | M | M | M | M | M | M | MF |  | *melanica* |
| *Drosophila robusta* | *robusta* | *robusta* | DSSC (15020-1111.01) | M | M | M | M | M | M | M | M | MF | KF632684 | *melanica* |
| *Drosophila sordidula* | *robusta* | *robusta* | DSSC (15020-1121.00) | M | M | M | M | M | M | M | M | MF |  | *melanica* |
| *Drosophila tsigana* | *melanica* | *-* | DSEU (E-15401) | M | M | M | M | M | M | M | M | MF | AY736513 | *melanica* |
| *Drosophila daruma* | *polychaeta* | *-* | DSSC (15100-0120.00) | M | ? | M | M | M | ? | M | ? | MF | AY736532 | *polychaeta* |
| *Drosophila fraburu* | *polychaeta* | *-* | DSSC (15000-2597.00) | M | M | M | M | M | M | M | ? | MF |  | *polychaeta* |
| *Drosophila hirtipes* | *polychaeta* | *-* | DSSC (15000-2595.00) | M | M | M | M | M | M | M | ? | MF | AF491633 | *polychaeta* |
| *Drosophila latifshahi* | *polychaeta* | *-* | David | M | M | M | M | M | ? | M | ? | MF | AY736536 | *polychaeta* |
| *Drosophila polychaeta* | *polychaeta* | *-* | DSSC (15100-1711.00) | M | M | M | M | M | M | M | ? | MF | AY736494 | *polychaeta* |
| *Drosophila biseriata* | *modified mouthparts* | *mitchelli* | DSSC (15291-2551.00) | MF | ? | M | M | M | M | loss | M | MF |  | *grimshawi* |
| *Drosophila crucigera* | *picture wing* | *grimshawi* | DSSC (15287-2531.00) | MF | M | M | M | M | M | loss | M | MF |  | *grimshawi* |
| *Drosophila eurypeza* | *modified mouthparts* | *setiger* | DSSC (15290-2581.00) | MF | ? | M | MF | M | M | loss | M | MF |  | *grimshawi* |
| *Drosophila grimshawi* | *picture wing* | *grimshawi* | DSSC (15287-2541.00) | MF | M | M | M | M | M | loss | M | MF | AY736533 | *grimshawi* |
| *Drosophila mimica* | *modified mouthparts* | *mimica* | DSSC (15292-2561.00) | MF | M | M | M | M | M | loss | M | MF | AY736537 | *grimshawi* |
| *Drosophila picticornis* | *picture wing* | *planitibia* | DSSC (15283-2491.00) | MF | M | M | M | M | M | loss | M | MF |  | *grimshawi* |
| *Drosophila soonae* | *modified mouthparts* | *mimica* | DSSC (15290-2591.00) | MF | M | M | M | M | M | loss | M | MF |  | *grimshawi* |
| *Scaptomyza anomala* | *-* | *-* | DSSC (33000-2661.00) | MF | M | M | M | M | ? | ? | ? | MF |  | *grimshawi* |
| *Scaptomyza pallida* | *-* | *-* | DSSC (34000-0010.00) | MF | M | M | M | M | M | ? | ? | MF | AY736542 | *grimshawi* |
| *Scaptomyza palmae* | *-* | *-* | DSSC (33000-2681.01) | MF | M | M | M | M | M | ? | M | MF |  | *grimshawi* |
| *Drosophila angularis* | *quinaria* | *-* | DSEU (E-16901) | MF | M | M | ? | M | M | ? | M | MF | AY736530 | *kuntzei* |
| *Drosophila brachynephros* | *quinaria* | *-* | DSEU (E-16501) | MF | M | M | M | M | M | ? | M | MF |  | *kuntzei* |
| *Drosophila deflecta* | *quinaria* | *-* | DSSC (15130-2018.00) | MF | M | M | M | M | M | M | M | MF |  | *kuntzei* |
| *Drosophila falleni* | *quinaria* | *-* | DSSC (15130-1961.00) | MF | ? | M | M | M | M | ? | ? | MF |  | *kuntzei* |
| *Drosophila innubila* | *quinaria* | *-* | Jaenike | MF | M | M | M | M | M | ? | M | MF |  | *kuntzei* |
| *Drosophila kuntzei* | *quinaria* | *-* | Cariou | MF | M | M | M | M | M | M | M | MF | AF491634 | *kuntzei* |
| *Drosophila nigromaculata* | *quinaria* | *-* | DSEU (E-14201) | MF | M | M | M | M | M | ? | M | MF | AY736483 | *kuntzei* |
| *Drosophila palustris* | *quinaria* | *-* | DSSC (15130-2001.00) | MF | M | M | M | M | M | M | M | MF | AY736538 | *kuntzei* |
| *Drosophila phalerata* | *quinaria* | *-* | DSSC (15130-2031.00) | MF | M | M | M | M | M | ? | M | MF | AY736492 | *kuntzei* |
| *Drosophila quinaria* | *quinaria* | *-* | DSSC (15130-2011.00) | MF | M | M | M | M | M | M | M | MF |  | *kuntzei* |
| *Drosophila recens* | *quinaria* | *-* | Jaenike | MF | M | M | M | M | M | ? | M | MF |  | *kuntzei* |
| *Drosophila subpalustris* | *quinaria* | *-* | DSSC (15130-2071.00) | MF | M | M | M | M | M | M | M | MF | AY736539 | *kuntzei* |
| *Drosophila transversa* | *quinaria* | *-* | Jaenike | MF | M | M | M | M | M | ? | M | MF | AY736511 | *kuntzei* |
| *Drosophila tenebrosa* | *quinaria* | *-* | Jaenike | MF | M | M | M | M | ? | ? | M | MF | JF735881 | *kuntzei* |
| *Drosophila neotestacea* | *testacea* | *-* | Jaenike | MF | M | M | M | MF | ? | ? | MF | MF |  | *cardini* |
| *Drosophila orientacea* | *testacea* | *-* | Jaenike | MF | M | M | M | MF | M | ? | MF | MF |  | *cardini* |
| *Drosophila putrida* | *testacea* | *-* | DSSC (15150-2101.00) | MF | ? | M | M | M | M | ? | ? | MF |  | *cardini* |
| *Drosophila testacea* | *testacea* | *-* | David; Jaenike | MF | M | M | M | MF | M | ? | MF | MF |  | *cardini* |
| *Drosophila bandeirantorum* | *tripunctata* | *III* | Klaczko | MF | M | M | M | M | M | M | MF | MF |  | *cardini* |
| *Drosophila bipunctata* | *tripunctata* | *III* | Klaczko | MF | M | M | M | M | M | ? | ? | MF |  | *cardini* |
| *Drosophila cuaso* | *tripunctata* | *II* | Klaczko | MF | M | M | M | M | M | ? | MF | MF |  | *cardini* |
| *Drosophila frotapessoai* | *tripunctata* | *III* | Klaczko | MF | ? | M | M | M | M | ? | MF | MF |  | *cardini* |
| *Drosophila mediodiffusa* | *tripunctata* | *IV* | DSSC (15220-2351.04) | MF | ? | M | M | M | M | M | MF | MF |  | *cardini* |
| *Drosophila mediopicta* | *tripunctata* | *III* | Klaczko | MF | ? | M | M | M | M | M | MF | MF |  | *cardini* |
| *Drosophila mediopictoides* | *tripunctata* | *III* | DSSC (15220-2371.00) | MF | ? | M | M | M | M | M | MF | MF | AY733055 | *cardini* |
| *Drosophila mediopunctata* | *tripunctata* | *II* | Klaczko | MF | M | M | M | M | M | M | MF | MF |  | *cardini* |
| *Drosophila mediosignata* | *tripunctata* | *II* | Klaczko | MF | M | M | M | M | M | M | MF | MF |  | *cardini* |
| *Drosophila mediostriata* | *tripunctata* | *III* | DSSC (15220-2391.00, 15220-2341.00) | MF | M | M | M | M | M | M | MF | MF |  | *cardini* |
| *Drosophila metzii* | *tripunctata* | *IV* | Klaczko | MF | M | M | M | M | M | M | MF | MF | AY744447 | *cardini* |
| *Drosophila nappae* | *tripunctata* | *I* | Klaczko | MF | M | M | M | M | ? | M | MF | MF |  | *cardini* |
| *Drosophila paraguayensis* | *tripunctata* | *II* | Klaczko; Loreto/Robe | MF | M | M | M | M | M | M | MF | MF |  | *cardini* |
| *Drosophila paramediostriata* | *tripunctata* | *III* | Klaczko | MF | ? | M | M | M | M | M | MF | MF |  | *cardini* |
| *Drosophila roehrae* | *tripunctata* | *II* | Klaczko | MF | M | M | M | M | M | ? | MF | MF |  | *cardini* |
| *Drosophila trifilum* | *tripunctata* | *I* | Klaczko | MF | M | M | M | M | M | M | MF | MF |  | *cardini* |
| *Drosophila tripunctata* | *tripunctata* | *IV* | DSSC (15220-2401.02) | MF | M | M | M | M | M | M | MF | MF |  | *cardini* |
| *Drosophila unipunctata* | *tripunctata* | *II* | DSSC (15220-2411.00) | MF | M | M | M | M | M | M | MF | MF |  | *cardini* |
| *Drosophila I44F5 (V3)* | *tripunctata* | *-* | Vilela | MF | M | M | ? | M | M | M | MF | MF |  | *cardini* |
| *Drosophila acutilabella* | *cardini* | *cardini* | DSSC (15181-2171.01) | MF | M | M | M | M | M | M | MF | MF |  | *cardini* |
| *Drosophila arawakana arawakana* | *cardini* | *dunni* | DSSC (15182-2261.00) | MF | M | M | M | M | M | M | MF | MF | AF491630 | *cardini* |
| *Drosophila arawakana kittensis* | *cardini* | *dunni* | DSSC (15182-2260.00) | MF | M | M | M | M | M | M | MF | MF |  | *cardini* |
| *Drosophila belladunni* | *cardini* | *dunni* | DSSC (15182-2271.01) | MF | ? | M | M | M | M | M | MF | MF |  | *cardini* |
| *Drosophila cardini* | *cardini* | *cardini* | DSSC (15181-2181.03); Klaczko | MF | M | M | M | M | M | M | MF | MF | AF462599 | *cardini* |
| *Drosophila cardinoides* | *cardini* | *cardini* | DSSC (15181-2191.00) | MF | M | M | M | M | M | M | MF | MF |  | *cardini* |
| *Drosophila caribiana* | *cardini* | *dunni* | DSSC (15182-2281.00) | MF | M | M | M | M | M | M | MF | MF | AY733050 | *cardini* |
| *Drosophila dunni dunni* | *cardini* | *dunni* | DSSC (15182-2291.00) | MF | ? | M | M | M | M | M | MF | MF |  | *cardini* |
| *Drosophila dunni thomasensis* | *cardini* | *dunni* | DSSC (15182-2301.00) | MF | ? | M | M | M | M | M | MF | MF |  | *cardini* |
| *Drosophila neocardini* | *cardini* | *cardini* | DSSC (15181-2201.00) | MF | M | M | M | M | M | M | MF | MF |  | *cardini* |
| *Drosophila nigrodunni* | *cardini* | *dunni* | DSSC (15182-2311.00) | MF | M | M | M | M | M | M | MF | MF |  | *cardini* |
| *Drosophila parthenogenetica* | *cardini* | *cardini* | DSSC (15181-2221.00) | MF | M | M | M | M | M | M | MF | MF |  | *cardini* |
| *Drosophila polymorpha* | *cardini* | *cardini* | DSSC (15181-2231.00); Klaczko | MF | M | M | M | M | M | M | MF | MF | AY736495 | *cardini* |
| *Drosophila procardinoides* | *cardini* | *cardini* | DSSC (15181-2241.00) | MF | ? | M | M | M | M | M | MF | MF |  | *cardini* |
| *Drosophila similis* | *cardini* | *dunni* | DSSC (15182-2321.00) | MF | ? | M | M | M | M | M | MF | MF |  | *cardini* |
| *Drosophila atrata* | *calloptera* | *-* | Klaczko | MF | M | M | M | M | ? | M | MF | MF |  | *cardini* |
| *Drosophila bizonata* | *bizonata* | *-* | DSEU (E-12801) | MF | M | M | M | MF | ? | ? | MF | MF |  | *cardini* |
| *Drosophila funebris* | *funebris* | *funebris* | DSSC (15120-1911.00); Cariou | MF | M | M | M | M | M | M | MF | MF | AF335557 | *cardini* |
| *Drosophila griseolineata* | *guarani* | *-* | DSSC (15171-2131.03); Klaczko | MF | M | M | M | M | M | M | MF | MF |  | *cardini* |
| *Drosophila guaraja* | *guarani* | *guaramunu* | Klaczko | MF | M | M | M | M | M | M | MF | MF |  | *cardini* |
| *Drosophila guaru* | *guarani* | *guarani* | Klaczko; Loreto/Robe | MF | M | M | M | M | M | M | MF | MF | AF491631 | *cardini* |
| *Drosophila guttifera* | *guttifera* | *-* | DSSC (15130-1971.01) | MF | M | M | M | M | M | M | M | MF |  | *kuntzei* |
| *Drosophila macrospina limpiensis* | *funebris* | *macrospina* | DSSC (15120-1921.00) | MF | ? | M | M | M | M | ? | MF | MF |  | *cardini* |
| *Drosophila macrospina macrospina* | *funebris* | *macrospina* | DSSC (15120-1931.00) | MF | ? | M | M | M | M | M | MF | MF |  | *cardini* |
| *Drosophila maculifrons* | *guarani* | *guaramunu* | Loreto/Robe | MF | ? | M | M | M | M | ? | MF | MF |  | *cardini* |
| *Drosophila ornatifrons* | *guarani* | *guarani* | Klaczko; Loreto/Robe | MF | M | M | M | M | M | M | MF | MF |  | *cardini* |
| *Drosophila ornatipennis* | *calloptera* | *-* | DSSC (15160-2121.00) | MF | M | M | M | M | M | ? | MF | MF |  | *cardini* |
| *Drosophila pallidipennis* | *pallidipennis* | *-* | DSSC (15210-2331.01) | MF | M | M | M | M | M | M | MF | MF | AY736487 | *cardini* |
| *Drosophila ponera* | *-* | *-* | DSSC (15000-0656.00) | ? | M | M | M | M | M | M | M | MF |  | *kuntzei* |
| *Drosophila quadrum* | *calloptera* | *-* | Vilela | MF | M | M | M | M | M | M | MF | MF |  | *cardini* |
| *Drosophila sternopleuralis* | *histrio* | *-* | DSSC (15270-2461.01) | MF | M | M | M | M | M | M | M | MF | AY736505 | *sternopleuralis* |
| *Drosophila subbadia* | *guarani* | *guarani* | DSSC (15172-2161.00) | MF | M | M | M | M | M | M | MF | MF |  | *cardini* |
| *Drosophila peixotoi* | *guarani* | *guarani* | Carvalho/Vaz | MF | M | M | M | M | ? | M | MF | MF |  | *cardini* |
| *Drosophila butantan* | *guarani* | *-* | Vilela | MF | M | M | M | M | M | M | MF | MF |  | *cardini* |
| *Drosophila albomicans* | *immigrans* | *nasuta* | DSSC (15112-1751.00); David | MF | M | M | M | M | M | M | M | MF | AF462595 | *albomicans* |
| *Drosophila curviceps* | *immigrans* | *curviceps* | DSEU (E-15101) | MF | M | M | M | M | M | M | M | MF |  | *albomicans* |
| *Drosophila formosana* | *immigrans* | *immigrans* | DSSC (15111-1721.00) | MF | M | M | M | M | M | M | M | MF | AF462601 | *albomicans* |
| *Drosophila hypocausta* | *immigrans* | *hypocausta* | DSSC (15115-1871.01) | MF | M | M | M | M | M | M | M | MF | AY733043 | *albomicans* |
| *Drosophila immigrans* | *immigrans* | *immigrans* | DSSC (15111-1731.01) | MF | M | M | M | M | M | M | M | MF | AF491632 | *albomicans* |
| *Drosophila kepulauana* | *immigrans* | *nasuta* | DSSC (15112-1761.01) | MF | M | M | M | M | M | M | M | MF | AY733044 | *albomicans* |
| *Drosophila kohkoa* | *immigrans* | *nasuta* | DSSC (15112-1771.01) | MF | M | M | M | M | M | M | M | MF |  | *albomicans* |
| *Drosophila nasuta* | *immigrans* | *nasuta* | DSSC (15112-1781.00); David | MF | M | M | M | M | M | M | M | MF | AY733059 | *albomicans* |
| *Drosophila neohypocausta* | *immigrans* | *hypocausta* | DSSC (15115-1881.00) | MF | ? | M | M | M | M | M | M | MF |  | *albomicans* |
| *Drosophila neonasuta* | *immigrans* | *nasuta* | David | MF | M | M | M | M | M | M | M | MF |  | *albomicans* |
| *Drosophila pallidifrons* | *immigrans* | *nasuta* | DSEU (E-19901) | MF | M | M | M | M | ? | M | M | MF | AY736486 | *albomicans* |
| *Drosophila pararubida* | *immigrans* | *hypocausta* | DSSC (15115-1891.00) | MF | M | M | M | M | M | M | M | MF |  | *albomicans* |
| *Drosophila pulaua* | *immigrans* | *nasuta* | DSSC (15112-1801.00) | MF | M | M | M | M | M | M | M | MF |  | *albomicans* |
| *Drosophila quadrilineata* | *immigrans* | *quadrilineata* | David | MF | ? | M | M | M | ? | ? | ? | MF |  | *albomicans* |
| *Drosophila ruberrima* | *immigrans* | *immigrans* | DSEU (E-14601) | MF | M | M | M | M | ? | ? | M | MF | AY736501 | *albomicans* |
| *Drosophila rubida* | *immigrans* | *hypocausta* | DSSC (15115-1901.01) | MF | M | M | M | M | M | ? | M | MF | AY736502 | *albomicans* |
| *Drosophila siamana* | *immigrans* | *hypocausta* | DSEU (E-22701) | MF | M | M | M | M | M | M | M | MF | AY736504 | *albomicans* |
| *Drosophila signata* | *immigrans* | *immigrans* | DSSC (15111-1741.00) | MF | M | M | M | M | M | M | M | MF |  | *albomicans* |
| *Drosophila sulfurigaster albostrigata* | *immigrans* | *nasuta* | DSSC (15112-1811.00) | MF | M | M | M | M | M | M | M | MF |  | *albomicans* |
| *Drosophila sulfurigaster bilimbata* | *immigrans* | *nasuta* | DSSC (15112-1821.00) | MF | M | M | M | M | M | M | M | MF |  | *albomicans* |
| *Drosophila sulfurigaster sulfurigaster* | *immigrans* | *nasuta* | DSSC (15112-1831.00); David | MF | M | M | M | M | M | M | M | MF | AY736508 | *albomicans* |
| *Drosophila ustulata* | *immigrans* | *immigrans* | David | MF | M | M | M | M | M | M | M | MF | AY736541 | *albomicans* |
| *Samoaia attenuata* | *attenuata* | *-* | Finet | MF | ? | M | M | M | ? | ? | M | MF |  | *albomicans* |
| *Samoaia hirta* | *attenuata* | *-* | Finet | MF | ? | M | M | M | ? | ? | M | MF |  | *albomicans* |
| *Samoaia leonensis* | *leonensis* | *-* | DSSC (80000-2761.00, 80000-2761.03) | MF | M | M | M | M | ? | ? | M | MF | EU161100 | *albomicans* |
| *Samoaia ocellaris* | *leonensis* | *-* | David; Finet | MF | ? | M | M | M | ? | M | M | MF |  | *albomicans* |
| *Zaprionus africanus* | *armatus* | *-* | David | ? | M | M | M | M | MF | M | M | MF |  | *Zaprionus* |
| *Zaprionus bogoriensis* | *-* | *-* | David | M | M | M | M | M | MF | ? | MF | MF | AY736516 | *Zaprionus* |
| *Zaprionus camerounensis* | *armatus* | *-* | DSSC (50001-1010.00) | M | M | M | M | M | MF | ? | M | MF | EF458332 | *Zaprionus* |
| *Zaprionus capensis* | *armatus* | *-* | David | M | M | M | M | M | MF | ? | M | MF | EF458326 | *Zaprionus* |
| *Zaprionus davidi* | *armatus* | *-* | DSSC (50001-1040.00) | M | M | M | M | M | MF | ? | M | MF | EF458323 | *Zaprionus* |
| *Zaprionus gabonicus* | *armatus* | *-* | DSSC (50002-2755.00) | M | M | M | M | M | ? | ? | M | MF |  | *Zaprionus* |
| *Zaprionus ghesquierei* | *inermis* | *-* | DSSC (50000-2743.00) | ? | M | M | M | M | MF | M | M | MF | AY736518 | *Zaprionus* |
| *Zaprionus indianus* | *armatus* | *-* | Bitner-Mathé | M | M | M | M | M | MF | ? | M | MF | EF458322 | *Zaprionus* |
| *Zaprionus inermis* | *inermis* | *-* | DSSC (50000-2746.00) | M | M | M | M | M | MF | M | M | MF | AY736519 | *Zaprionus* |
| *Zaprionus kolodkinae* | *armatus* | *-* | DSSC (50000-2748.00) | M | M | M | M | M | ? | M | M | MF | AY736520 | *Zaprionus* |
| *Zaprionus lachaisei* | *armatus* | *-* | DSSC (50002-2750.00) | M | ? | M | M | M | MF | ? | M | MF | EF458330 | *Zaprionus* |
| *Zaprionus mascariensis* | *armatus* | *-* | DSSC (50001-0002.00) | M | M | M | M | M | MF | M | MF | MF | AY736522 | *Zaprionus* |
| *Zaprionus neglectus* | *inermis* | *-* | DSSC (50003-2753.00) | M | ? | M | M | M | MF | M | M | MF |  | *Zaprionus* |
| *Zaprionus ornatus* | *armatus* | *-* | DSSC (50002-2749.00) | M | M | M | M | M | MF | ? | M | MF |  | *Zaprionus* |
| *Zaprionus proximus* | *armatus* | *-* | DSSC (50002-2756.00) | M | M | M | M | M | MF | ? | M | MF |  | *Zaprionus* |
| *Zaprionus sepsoides* | *armatus* | *-* | DSSC (50000-2744.00) | M | M | M | M | M | MF | M | M | MF | AY736523 | *Zaprionus* |
| *Zaprionus taronus* | *armatus* | *-* | DSSC (50001-1020.00) | M | M | M | M | M | MF | ? | M | MF | EF458324 | *Zaprionus* |
| *Zaprionus tsacasi* | *armatus* | *-* | DSSC (50000-2751.00) | M | M | M | M | M | MF | ? | M | MF | EF458325 | *Zaprionus* |
| *Zaprionus tuberculatus* | *armatus* | *-* | DSSC (50001-0001.06); Bitner-Mathé | M | M | M | M | M | MF | M | M | MF | AY736524 | *Zaprionus* |
| *Hirtodrosophila confusa* | *quadrivittata* | *confusa* | David | MF | M | M | M | M | M | M | MF | MF | AF335559 | *Hirtodrosophila* |
| *Hirtodrosophila duncani* | *duncani* | *-* | DSSC (92000-0075.00) | MF | M | M | M | MF | M | ? | ? | MF |  | *duncani* |
| *Hirtodrosophila ramulosa* | *-* | *-* | Carvalho/Vaz | MF | ? | M | M | M | ? | ? | ? | MF |  | *Hirtodrosophila* |
| *Hirtodrosophila spinipes* | *-* | *-* | Cariou | MF | ? | M | M | MF | ? | M | M | ? |  | *Hirtodrosophila* |
| *Hirtodrosophila thoracis* | *thoracis* | *-* | Carvalho/Vaz | MF | ? | M | ? | M | ? | ? | MF | MF |  | *Hirtodrosophila* |
| *Hirtodrosophila T17W1B* | *hirticornis* | *-* | Carvalho/Vaz | MF | M | M | M | M | M | M | ? | MF |  | *Hirtodrosophila* |
| *Hirtodrosophila T21W1A* | *hirticornis* | *-* | Carvalho/Vaz | MF | M | M | M | M | ? | ? | MF | MF |  | *Hirtodrosophila* |
| *Hirtodrosophila T56F3* | *hirticornis* | *-* | Carvalho/Vaz | MF | ? | ? | M | M | ? | ? | ? | MF |  | *Hirtodrosophila* |
| *Hirtodrosophila T56W10/11* | *hirticornis* | *-* | Carvalho/Vaz | MF | M | M | M | M | M | ? | ? | MF |  | *Hirtodrosophila* |
| *Hirtodrosophila T57W4* | *hirticornis* | *-* | Carvalho/Vaz | MF | M | M | M | M | M | ? | MF | MF |  | *Hirtodrosophila* |
| *Hirtodrosophila T60W1* | *thoracis* | *-* | Carvalho/Vaz | MF | M | M | M | M | M | M | MF | MF |  | *Hirtodrosophila* |
| *Hirtodrosophila T60W2* | *-* | *-* | Carvalho/Vaz | MF | M | M | M | M | M | ? | MF | MF |  | *Hirtodrosophila* |
| *Hirtodrosophila T60W3* | *-* | *-* | Carvalho/Vaz | MF | M | M | M | M | ? | M | MF | MF |  | *Hirtodrosophila* |
| *Zygothrica atriangula* | *atriangula* | *-* | Carvalho/Vaz | MF | ? | M | ? | M | M | ? | MF | MF |  | *Hirtodrosophila* |
| *Zygothrica microeristes* | *dispar* | *aldrichii* | Carvalho/Vaz | MF | ? | M | M | M | ? | ? | ? | MF |  | *Hirtodrosophila* |
| *Zygothrica nigropleura* | *dispar* | *aldrichii* | Carvalho/Vaz | MF | ? | M | M | M | ? | ? | ? | MF |  | *Hirtodrosophila* |
| *Zygothrica sectipoeyi* | *sectipoeyi* | *-* | Carvalho/Vaz | MF | ? | ? | M | ? | ? | M | MF | MF |  | *Hirtodrosophila* |
| *Zygothrica vitticlara* | *vittimaculosa* | *-* | Carvalho/Vaz | MF | ? | M | M | M | ? | ? | ? | ? |  | *Hirtodrosophila* |
| *Zygothrica T17W1C* | *dispar* | *-* | Carvalho/Vaz | MF | ? | M | ? | ? | ? | ? | ? | MF |  | *Hirtodrosophila* |
| *Drosophila denieri* | *-* | *-* | Goni | MF | M | M | M | M | M | M | ? | MF |  | *cardini* |
| *Drosophila repletoides* | *tumiditarsus* | *-* | DSSC (15250-2451.00) | ? | M | M | M | M | M | M | M | MF | AY736500 | *repletoides* |
| *Drosophila T54F3* | *-* | *-* | Carvalho/Vaz | MF | M | M | M | M | M | ? | ? | MF |  | *cardini* |
| *Mycodrosophila sp.* | *-* | *-* | Carvalho/Vaz | MF | ? | M | M | M | M | M | MF | MF |  | *Hirtodrosophila* |
| *Mycodrosophila neoprojectans* | *-* | *-* | Krsticevic | MF | ? | ? | M | M | ? | ? | MF | MF |  | *Hirtodrosophila* |
| *Dettopsomyia nigrovittata* | *-* | *-* | Carvalho/Vaz | MF | M | M | M | M | M | M | M | MF |  | *Dettopsomyia* |
| *Drosophila busckii* | *busckii* | *-* | DSSC (13000-0081.00) | ? | M | M | M | M | M | M | MF | MF |  | *busckii* |

##### Table B. Degenerate PCR success rate in the nine sampled genes. The overall success rate was 94%: 3378 reactions produced M or MF results and 215 failed.

| Gene | M | MF | Genomic loss | Failure | % PCR success |
| --- | --- | --- | --- | --- | --- |
| *CG11719* | 133 | 259 |  | 8 | 98.0 |
| *JY-alpha* | 276 | 80 |  | 44 | 89.0 |
| *kl-2* | 375 | 22 |  | 3 | 99.3 |
| *kl-3* | 340 | 54 |  | 6 | 98.5 |
| *kl-5* | 310 | 87 |  | 3 | 99.3 |
| *ORY* | 294 | 69 |  | 37 | 90.8 |
| *Ppr-Y* | 277 | 52 | 7 | 64 | 84.0 |
| *PRY* | 165 | 191 |  | 44 | 89.0 |
| *WDY* | 68 | 326 |  | 6 | 98.5 |
|  |  |  |  |  |  |
| Total | 2238 | 1140 | 7 | 215 | 94.0 |

##### Table C. Gene loss events in the Y chromosome.

| Affected gene | Affected species (number of species in our sample) | Node | Comments |
| --- | --- | --- | --- |
| All genes (11) | *montium* subgroup (40 spp.) | Root of *montium* subgroup | Y incorporation event. |
| All genes (5) | *pseudoobscura* + *affinis* subgroups (12 spp.) | Root of *affinis* and *pseudoobscura* subgroups | Y incorporation event previously described in ref . |
| All genes (7) | *D. limensis* | - | Probably a Y chromosome non-disjunction. (SI section "The case of *D. limensis*"). |
| *PRY* + *JYα* | *obscura* group (22 spp.) | Root of the *obscura* group. | These genes are side-by-side in the X chromosome in *D. pseudoobscura* (Fig. A in S1 File) |
| *CG11719* | *D. flexa* | - | Belongs to the *Siphlodora* subgenus |
| *CG11719* | *Drosophila* sp. T21W2 | - | Belongs to *mesophragmatica* group. The gene is Y-linked for the other four sampled species from this group. |
| *kl-3* | *D. eurypeza* | - | The gene is Y-linked for the other seven sampled species of the *Hawaiian* *Drosophila* group. |
| *kl-5* | *testacea* group (3 spp.) *+  D. bizonata* | Root of the *testacea* group + *bizonata* group | Event previously described for three species of the *testacea* group . *D. bizonata* (of homonymous single species group) is related with *testacea* group . |
| *kl-5* | *Hirtodrosophila spinipes* | - | - |
| *Ppr-Y* | *D. canalinea* | - | *-* |
| *JY-alpha* | “*melanogaster* clade” (20 spp.) | Inside *melanogaster* group after the divergence between *melanogaster* and *ficusphila* subgroups. | Event previously described for the *melanogaster* subgroup . Here we found that the event occurred after the divergence between *D. ficusphila* and subgroups *melanogaster* *eugracilis* *takahashii* and part of the *suzukii* subgroup (*D. suzukii* *D. biarmipes* and *D. mimetica*) which are a monophyletic clade . |
| *ORY* | *Zaprionus* genus (20 spp.) | root of the *Zaprionus* genus. | Yassin et al. 2010 showed that *Zaprionus* genus is polyphyletic. However all species in our sample belong to the main monophyletic clade . |
| *ORY* | *D. nannoptera* | - | The gene is Y-linked for the other three sampled species of the *nannoptera* group. |
| *Ppr-Y* | *D. annulimana* | - | The gene is Y-linked for the other five sampled species of the *annulimana* group. |
| *Ppr-Y* | *Hawaiian Drosophila* (7 spp.) | Root of the *Hawaiian* *Drosophila* clade. | Sole confirmed case that the gene have disappeared from the genome.; previously described |
| *PRY* | *repleta* clade (93 spp.) | Root of the *repleta* clade | The “*repleta* clade” is a monophyletic branch including the sampled *Drosophila* Subgenus groups *annulimana, bromeliae, canalinea, caponei, coffeata, dreyfusi, flavopilosa, mesophragmatica, morelia,* *nannoptera*, *repleta*, *xanthopallescens*, the *Siphlodora* Subgenus and two unclassified species (*D. calathea* and T52W1) . |
| *PRY* | Part of the *immigrans-tripunctata* radiation (55 spp) | After the divergence between *tripunctata* and *immigrans* groups. | The *immigrans-tripunctata* Radiation is a *Drosophila* Subgenus monophyletic branch including groups where *PRY* is “M” or “MF” PCR results. The sampled groups with “MF” results are *cardini* *tripunctata* g*uarani calloptera pallidipennis testacea bizonata and funebris*; while the groups with “M” results are *immigrans* (the “basal group”) *guttifera quinaria* and *histrio*. The phylogenetic relationships of these groups are unclear and the *PRY* results could be explained from a single or multiples events to out the Y chromosome . |
| *PRY* | *Zaprionus mascariensis* (1 sp) | - | Taking into consideration the phylogeny the *Z. bogoriensis* and *Z. mascariensis* events are independent |
| *PRY* | *Zaprionus* (*Anaprionus*) *bogoriensis* (1 sp) | - | Taking into consideration the phylogeny the *Z. bogoriensis* and *Z. mascariensis* events are independent |
| *PRY* | *Zygothrica* genus group (21 spp) | Root of the *Hirtodrosophila* *Zygothrica* and *Mycodrosophila* genera. | The *Zygothrica* genus group is a monophyletic clade including the mushroom-associated genera *Hirtodrosophila* *Zygothrica* and *Mycodrosophila* .  *PRY* is Y-linked (“M”) in two *Hirtodrosophila* species: *H. spinipes* and *H. duncani*. Both are not related with the *Hirtodrosophila* genus the first seems related to other species of the *Drosophila* (*Drosophila*) (unpublished) and the second belongs to *Sophophora* . |
| *PRY* | *D. busckii* (1 sp) | - | *-* |

##### Table D. Morphology of Y chromosomes of *montium* species, and the number of Y-linked genes. There is no clear association between the two characters.

| Species | Y chromosome morphology a | Number of retained  Y-linked genes | Reference |
| --- | --- | --- | --- |
| *D. auraria* | SJ / R | 0 | / |
| *D. baimaii* | LJ / R | 1 | / |
| *D. barbarae* | LJ / R | 2 | / |
| *D. biauraria* | SR | 0 |  |
| *D. bicornuta* | SR | 2 |  |
| *D. birchii* | LJ | 3 |  |
| *D. bocki* | LV | 4 |  |
| *D. bocqueti* | SR | 2 |  |
| *D. greeni* | SR | 2 |  |
| *D. jambulina* | MV / V or R | 2 | / |
| *D. kanapiae* | SDH | 4 |  |
| *D. kikkawai* | LV / R | 4 | / |
| *D. lacteicornis* | R | 0 |  |
| *D. leontia* | LV / V | 3 | / |
| *D. lini* | SJ / J | 3 | / |
| *D. mayri* | LJ | 2 |  |
| *D. nikananu* | MV | 6 |  |
| *D. orosa* | LJ | 2 |  |
| *D. parvula* | SDH / J | 3 | / |
| *D. punjabiensis* | R | 2 |  |
| *D. rufa* | SJ | 0 |  |
| *D. seguyi* | LJ | 2 |  |
| *D. serrata* | SV | 2 |  |
| *D. tani* | J | 0 |  |
| *D. triauraria* | SR | 0 |  |
| *D. truncata* | V | 2 |  |
| *D. tsacasi* | J | 2 |  |
| *D. vulcana* | SR | 2 |  |

a Y chromosome morphology: R, rod shape (acrocentric); J, J-shaped (submetacentric), SJ and LJ, small and large J-shaped, respectively; V, V-shaped (metacentric); SV, MV and LV, small, medium and large V-shaped, respectively; SDH, small dense and heterochromatic. When two references are available for the same species we listed both.

Table E. Causes of re-appearance of Y-linked genes in the *pseudoobscura* and *affinis* subgroups. The hypothesis of general increase in gene gain is rejected (*P* = 0.035; two-tailed Fisher exact test) ; only formerly Y-linked genes seem to move to the Y.

|  | Moved to the Y | Not moved to the Y |
| --- | --- | --- |
| Present in the *aff.* / *pse.* ancestor Y ^a^ | 3 ^b^ | 2 ^c^ |
| Absent from in the *aff.* / *pse.* ancestor Y | 0 | 8 ^d^ |

^a^ Fig 4 and Fig A in S1 File data.

^b^ genes *kl-2* *ORY* *ARY*

^c^ genes *kl-3* *Ppr-Y*

^d^ genes *CG11719* *CG2964* *PRY* *kl-5* *WDY* *CG14339* *Pp1Y1* *Pp1Y2*

##### Table F. *Drosophila* species and groups with X-autosome fusions. Except for the *affinis* and *pseudoobscura* subgroups, none of them has Y incorporations, which shows that Y incorporations are not a necessary consequence of X-A fusions.

| Affected species/groups | Sampled species | Comment | Reference |
| --- | --- | --- | --- |
| *willistoni* gr. + *saltans* gr. | 21 | X-autosome (D element) |  |
| *affinis* and *pseudoobscura* subgroups (*obscura* group) | 12 | X-autosome (D element) |  |
| *D. albomicans* (*immigrans* gr.) | 1 | X-autosome (C+D elements) |  |
| *D. fuscolineata* (*coffeata* gr.) | 1 | Metacentric X chromosome and the karyotype only have 5 arms + dot chromosome indicated a possible X-autosome fusion. |  |
| *nannoptera* gr. | 3 | Metacentric X chromosome (V-shaped) is common in many nannoptera species (*D. nannoptera* *D. pachea* *D. acanthoptera*). and the authors believe that it is a product of a fusion X-autosome. |  |
| *D. americana americana* | 1 | X-autosome (B element) |  |
| *melanica* gr. | 4 | X-autosome (D element). Observed in four related species (*D. melanica D. paramelanica D. euronotus* and *D. nigromelanica*) |  |
| *robusta* gr. | 2 | X-autosome (D element). Observed in two related species (*D. robusta* and *D. sordidula*) |  |
| *D. dreyfusi* (*dreyfusi* gr.) | 1 | Submetacentric X chromosome. The karyotype only has 5 arms + dot chromosome indicated a possible X-autosome fusion. |  |
| *D. busckii* | 1 | X-autosome (F element) |  |

##### Table G. Genomic effects of the Y incorporation in *montium* and *pseudoobscura* subgroup species, and assembly comparisons.

| Species | Assembly | Technology | Ref. | Evidence for reduced intergenic distances | Evidence for reduced intron size | OOF indels a | % missing sequence b |
| --- | --- | --- | --- | --- | --- | --- | --- |
| *D. pseudoobscura* | AAFS00000000.1 | Sanger |  | Scaffold 396212 contains *kl-2*, *ARY* and the N-term of the *ORY* gene; CH396235 contains *Ppr-Y* and the C-term of *ORY*. | *kl-2*, *kl-3* and *ARY* genes were fully assembled in single scaffolds and normally size. | 0 | 0.1 |
| *D. pseudoobscura* | - | PacBio |  | All five formerly Y-linked genes in a single 320 kb region | All five formerly Y-linked genes fully assembled and normal size. | 0 | 0 |
| *D. kikkawai* | GCA_000224215.2 | Illumina + 454 |  | none found | none found (but assembly is highly fragmented) | 1 | 0 |
| *D. serrata* | [GCA_002093755.1](https://www.ncbi.nlm.nih.gov/assembly/1072081) | PacBio |  | scaffold NW_018367086 contains *PP1Y2* and the first exon of *Ppr-Y* | none found (but assembly is highly fragmented) | 21 | 23.0 |

a Total number of out-of-frame indels in the three genes that are shared between the Y incorporations in the three species (*kl-3, ARY, ORY*). The number of OOF indels was measured with GeneWise , using the *D. melanogaster* kl-3, ARY, and ORY proteins as the reference and the listed assemblies as the DNA source.

b Percentage of missing exonic sequence in the shared three genes (*kl-3, ARY, ORY*). The amount of missing exonic sequence was measured with GeneWise , using the *D. melanogaster* kl-3, ARY, and ORY proteins as the reference and the listed assemblies as the DNA source.

##### Table H. Gene gains by the Y chromosome in the 400 species sample.

| Affected gene | Affected species (number of species in our sample) | Node | Comments |
| --- | --- | --- | --- |
| *CG11719* | *virilis-repleta* Radiation (122 spp.) | After the divergence between *repleta* group and Hawaiian *Drosophila* group. | Event previously described by . Here we showed that the event included most of the groups of the *virilis-repleta* radiation. |
| *CG11719* | *Zaprionus* genus (20 spp.) | Ancestral of *Zaprionus* genus. | New result. |
| *kl-5* | All *melanogaster* group (126 spp.) | Ancestral of the *melanogaster* group | Event described by . |
| *kl-5* | All *Drosophila* subgenus (278 sp.) | Ancestral of *Drosophila* subgenus | Event described by . |
| *PRY* | Species from the “*repleta* clade” (6 spp.) | See the comments | New result. There are four events with “gene return” to Y chromosome in the *repleta* clade: *D. starmeri* + *D. venezolana* (*repleta* gr.) *D. ellisoni* + *D. aff. ellisoni*  (*repleta* gr.) *D. arapuan* (*annulimana* gr.) and *D. gasici* (*mesophragmatica* gr.). These events could be 4 independent return movements to Y chromosome or the 2 copies were maintained across the speciation with loss of the autosome copies in these 4 events. |
| *WDY* | All *melanogaster* group | Ancestral of the *melanogaster* group | Event described by . |

##### Table I. Comparison between PASTIS and molecular clock branch length estimates.

|  | Number of our sampled species | | Total branch length (Myr) | | |  |
| --- | --- | --- | --- | --- | --- | --- |
| Clade | Total | With *Amyrel* data | Yule | PASTIS | Molecular clock (RelTime) | |
| *ananassae* subgroup | 16 | 16 | 297.6 | 186.7 | 124.5 | |
| *montium* subgroup | 40 | 37 | 523.9 | 468.2 | 233.7 | |
| *pseudoobscura* + *affinis* subgroups | 12 | 10 | 98.8 | 71.0 | 51.2 | |

##### Table J. PCR primers

| Gene | Primer name | Sequence |
| --- | --- | --- |
| *Amyrel* | AMR_RELZONE2 | TCGTAAATTGGACCCAAGCG |
| *Amyrel* | AMR_RELAVALREV | CATACATTATGTGCGTTCG |
| *ARY* | kik_ARY_F1 | ACAAATGACGTTGATTATATAGATACTTGG |
| *ARY* | kik_ARY_R1 | ATTTCTTAGTCAATCGTTTCATACCTTC |
| *CG11719* | CG11719_EPMV_F1 | CGAGAAGATCGTNCCNGARCCNATGGT |
| *CG11719* | CG11719_WNTH_R1 | CAGGGCCRCARTGNGTRTTCCA |
| *CG11719* | CG11719_EPMV_Fnd | AGCGAGAAGATCGTGCC |
| *CG11719* | CG11719_WNTH_Rnd | CGCAGGGCCACAATG |
| *JY-alpha* | JYa_AHLW_F1 | ACTCAAAATCGAATGACTGTNGCNCAYYTNTGG |
| *JY-alpha* | JYa_IMDS_F1 | TCAAGTACATAAATCTTCTGCNATHATGGAYTCNTT |
| *JY-alpha* | JYa_MGGM_R1 | AAATCCTAGAACTCGTTCNCCCATNCCNCCCAT |
| *JY-alpha* | JYa_MGER_R1 | AGCATTAGATCACAAAATCCTAGAACNCKYTCNCCCAT |
| *JY-alpha* | JYa_MKGA_R1 | GATCTAGCATTCGTTCTGGNGCNCCYTTCAT |
| *JY-alpha* | JYa_AHLW_nd | CGCACTCAAAATCGAATGACTGT |
| *JY-alpha* | JYa_IMDS_nd | CGCTCAAGTACATAAATCTTCTGC |
| *JY-alpha* | JYa_MGGM_nd | AAATCCTAGAACTCGTTCACCCAT |
| *JY-alpha* | JYa_MGER_nd | CCAGCATTAGATCACAAAATCCTAGAAC |
| *kl-2* | kl2_KVME_F1 | CGCCCATGAARATHGARAARGTNATGGA |
| *kl-2* | kl2_QMQE_R1 | TGTTCTTGATCCARTTRTTNGCYTGCATYTG |
| *kl-2* | kl2_KVME_nd | ACGCGCCCATGAAGAT |
| *kl-2* | kl2_QMQE_nd | ACGTGTTCTTGATCCAGTTGTT |
| *kl-2* | kik_kl2_F | GAGGTCGTAATGCCCATGATAGA |
| *kl-2* | kik_kl2_R | TGTAAGCTGTCAACGAAAATACATACAT |
| *kl-3* | kl3_AMKE_F2 | GKGARCTGATGACBAAYRARGCNATGAARGA |
| *kl-3* | kl3_EMQD_R2 | CAGAATRGTSCKRATVGTRTCYTGCATYTC |
| *kl-3* | moj_AMKE_F1 | ATTAATGACGAATAAGGCAATGAAGGA |
| *kl-3* | moj_EMQD_R1 | CGAGAATAGTTCTTACAGTATCTTGCATTTC |
| *kl-3* | kl3_DKMD_F | GGCCAAGAARTGYCANGAYAARATGGA |
| *kl-3* | kl3_MYQT_R | GGAACTGCACCAGNGANGTYTGRTACAT |
| *kl-3* | kl3_AMKE_F2nd | gagctgatgaccaataaagc |
| *kl-3* | kl3_EMQD_R2nd | cagaatggtcctgatcgtat |
| *kl-3* | kl3_DKMD_Fnd | ACGGCCAAGAAGTGTCA |
| *kl-3* | kl3_MYQT_Rnd | GGAACTGCACCAGCGA |
| *kl-5* | FIFM_F | ggarccbttyathttyatgga |
| *kl-5* | MMVY_R | wrcarttraanacrtanaccatcat |
| *kl-5* | LGMM2 | gngcnytnggnatgatg |
| *kl-5* | P4 | ccnggrttcatngtdatraa |
| *kl-5* | ana_kl5_F1 | CTTGGGAACCGTTTATATTTATAGA |
| *kl-5* | ana_kl5_R1 | GAACAATTAAACACATAAACCATCAT |
| *kl-5* | FIFM_F_nd2 | ggaaccgttcattttcatgg |
| *kl-5* | MMVY_R_nd2 | tgcagttgaacacatacacca |
| *ORY* | ORY_YKNI_F1 | GCCATCTGGCAAARTAYAARAAYAT |
| *ORY* | ORY_IEKE_R1 | CTGCATTTGAYTTYTCYTTYTCDAT |
| *ORY* | ORY_YKNI_F1_nd | ACGCCATCTGGCAAAGTA |
| *ORY* | ORY_IEKE_R1_nd | GCGCTGCATTTGACTTCTC |
| *Pp1-Y1* | kik_PP1Y1_F10 | GAAGTTATAATGTCGGAGCCAATG |
| *Pp1-Y1* | kik_PP1Y1_R10 | AGGCTTCATGACTACCAAAGTAATGTT |
| *Pp1-Y2* | kik_PP1Y2_F10 | GCAGCAAATGACAACAGTAACAGA |
| *Pp1-Y2* | kik_PP1Y2_F12 | TCAGACCCTGACCCCAAAATAATG |
| *Pp1-Y2* | kik_PP1Y2_R12 | CTTAAGAACATAGAAAGAGCACAT |
| *Ppr-Y* | PPrY_FVEH_ns_F1 | GCCTAGCTTCAAGTTTYGTVGANCA |
| *Ppr-Y* | PPrY_MHGE_R1 | CAGGTGTATCWTCATCNTCNCCRTGCAT |
| *Ppr-Y* | PPrY_FVEH_F2 | GCCTAGCTTCAAGYTTYGTNGANCA |
| *Ppr-Y* | PPrY_MHGE_R2 | CAGGTGTATCWTCNTCNTCNCCRTGCAT |
| *Ppr-Y* | PPrY_FVEH_nd2 | CGCGCCTAGCTTCAAG |
| *Ppr-Y* | PPrY_MHGE_nd2 | GCGCAGGTGTATCATCGTC |
| *Ppr-Y* | moj_PPrY_F10 | TGAACGAGATGAAGAAATAAGG |
| *Ppr-Y* | moj_PPrY_R10 | CATCCCGATCATCCATACAC |
| *Ppr-Y* | mel_PPrY_F10 | GGAAATTGAAGATAAACAAGAAAAGGAGATACA |
| *Ppr-Y* | mel_PPrY_R10 | TCATGCAAATATAACTCCTGGGCCAT |
| *PRY* | PRY_YWWY_F1 | AGTCATAAGCAGagycARtaytggtggta |
| *PRY* | PRY_PWRF_R1 | GCGCTTTGTTCCRAANCKCCANGG |
| *PRY* | PRY_AHWK_F1 | ATAAACAACTGGTTACaygcnCaytggaa |
| *PRY* | PRY_MGNW_R1 | TTGGTTGAAATAARTACCANTTNCCCAT |
| *PRY* | PRY_YWWY_Fnd | AGTCATAAGCAGAGTCAATACTGGTGGTA |
| *PRY* | PRY_MGNW_Rnd | TTGGTTGAAATAAATACCACTTGCCCAT |
| *PRY* | PRY_AHWK_Fnd | ATAAACAACTGGTTACATGCACATTGGAA |
| *PRY* | PRY_PWRF_Rnd | CGCTTTGTTCCAAATCTCCAAGG |
| *PRY* | kik_PRY_F12 | AGTTGCTTATTTTATATGGGAATGGG |
| *PRY* | kik_PRY_R12 | ATCTATTCCAAAACGCCACGG |
| *WDY* | WDY_IWDV_F1 | GAGGACCAGATACTTTTGTTCGNATHTGGGAYGT |
| *WDY* | WDY_WNYN_R1 | CATATTTCGAACAACAACNGCRTTRTTRTARTTCCA |
| *WDY* | WDY_IKVW_F1 | CCGTGGATTACCACAARATHATHAARGTNTGG |
| *WDY* | WDY_VIWV_R1 | ACATACCGATCNACNACCCADATNAC |
| *WDY* | WDY_IWDV_nd | CGAGGACCAGATACTTTTGTTCG |
| *WDY* | WDY_IKVW_nd | GCCGTGGATTACCACAAGAT |
| *WDY* | WDY_WNYN_nd | GCATATTTCGAACAACAACCGC |
| *WDY* | WDY_VIWV_nd | ACATACCGATCGACTACCCA |

##### Table K. PCR Conditions

| **Gene** | **Product size** | **Primer Forward** | **Primer Reverse** | **Annealing** |
| --- | --- | --- | --- | --- |
| *Amyrel* | 1700 bp | AMR_RELZONE2 | AMR_RELAVALREV | 53 ºC |
| *ARY* | 310 bp | kik_ARY_F1 | kik_ARY_R1 | 52/48 ºC* |
| *CG11719* | 780 bp | CG11719_EPMV_F1 | CG11719_WNTH_R1 | 68/64 ºC* |
| *JY-alpha* | 516 bp | JYa_AHLW_F1 | JYa_MGGM_R1 | 64/60 ºC* |
| *JY-alpha* | 528 bp | JYa_AHLW_F1 | JYa_MGER_R1 | 60/56 ºC* |
| *JY-alpha* | 1095 bp | JYa_IMDS_F1 | JYa_MKGA_R1 | 64/60 ºC* |
| *kl-2* | 860 bp | kl2_KVME_F1 | kl2_QMQE_R1 | 58/54 ºC* |
| *kl-2* | 1650 bp | kik_kl2_F | kik_kl2_R | 50 ºC |
| *kl-3* | 920 bp | kl3_AMKE_F2 | kl3_EMQD_R2 | 56 ºC |
| *kl-3* | 920 bp | moj_AMKE_F1 | moj_EMQD_R1 | 52 ºC |
| *kl-3* | 1140 bp | kl3_DKMD_F | kl3_MYQT_R | 54 ºC |
| *kl-5* | 1600 bp | FIFM_F | MMVY_R | 53 ºC |
| *kl-5* | 270 bp | LGMM2 | P4 | 48 ºC |
| *kl-5* | 1600 bp | ana_kl5_F1 | ana_kl5_R1 | 50 ºC |
| *ORY* | 500 bp | ORY_YKNI_F1 | ORY_IEKE_R1 | 54 ºC |
| *Pp1-Y1* | 765 bp | kik_PP1Y1_F10 | kik_PP1Y1_R10 | 58 ºC |
| *Pp1-Y2* | 890 bp | kik_PP1Y2_F10 | kik_PP1Y2_R12 | 58 ºC |
| *Pp1-Y2* | 270 bp | kik_PP1Y2_F12 | kik_PP1Y2_R12 | 56 ºC |
| *Ppr-Y* | 380 bp | PPrY_FVEH_ns_F1 | PPrY_MHGE_R1 | 53 ºC |
| *Ppr-Y* | 380 bp | PPrY_FVEH_F2 | PPrY_MHGE_R2 | 56 ºC |
| *Ppr-Y* | 550 bp | moj_PPrY_F10 | moj_PPrY_R10 | 51 ºC |
| *Ppr-Y* | 550 bp | mel_PPrY_F10 | mel_PPrY_R10 | 44 ºC |
| *PRY* | 1300 bp | PRY_YWWY_F1 | PRY_PWRF_R1 | 62/58 ºC* |
| *PRY* | 1600 bp | PRY_AHWK_F1 | PRY_MGNW_R1 | 60/56 ºC* |
| *PRY* | 1600 bp | kik_PRY_F12 | kik_PRY_R12 | 54 ºC |
| *WDY* | 490 bp | WDY_IWDV_F1 | WDY_WNYN_R1 | 56/52 ºC* |
| *WDY* | 680 bp | WDY_IKVW_F1 | WDY_VIWV_R1 | 58/54 ºC* |

# * Anneling temperature in Touchdown PCR. 20 cycles decreasing -0.2 ºC by cycle. PCR protocols were deposited in [**dx.doi.org/10.17504/protocols.io.szyef7w**](http://dx.doi.org/10.17504/protocols.io.szyef7w) .

# References

1. Koerich LB, Wang X, Clark AG, Carvalho AB (2008) Low conservation of gene content in the *Drosophila* Y chromosome. Nature 456: 949-951.

2. Carvalho AB, Clark AG (2013) Efficient identification of Y chromosome sequences in the human and *Drosophila* genomes. Genome Res 23: 1894-1907.

3. Carvalho AB, Koerich LB, Clark AG (2009) Origin and evolution of Y chromosomes: *Drosophila* tales. Trends Genet 25: 270-277.

4. Lynch M, Conery JS (2003) The evolutionary demography of duplicate genes. J Struct Funct Genomics 3: 35-44.

5. Carvalho AB, Vicoso B, Russo CA, Swenor B, Clark AG (2015) Birth of a new gene on the Y chromosome of *Drosophila melanogaster*. Proc Natl Acad Sci USA 112: 12450-12455.

6. Dyer KA, White BE, Bray MJ, Piqué DG, Betancourt AJ (2011) Molecular evolution of a Y chromosome to autosome gene duplication in *Drosophila*. Mol Biol Evol 28: 1293.

7. Tamura K, Subramanian S, Kumar S (2004) Temporal patterns of fruit fly (*Drosophila*) evolution revealed by mutation clocks. Mol Biol Evol 21: 36-44.

8. Mooers A, Gascuel O, Stadler T, Li H, Steel M (2012) Branch lengths on birth-death trees and the expected loss of phylogenetic diversity. Syst Biol 61: 195-203.

9. Thomas GH, Hartmann K, Jetz W, Joy JB, Mimoto A, et al. (2013) PASTIS: an R package to facilitate phylogenetic assembly with soft taxonomic inferences. Methods Ecol Evol 4: 1011-1017.

10. Jetz W, Thomas GH, Joy JB, Hartmann K, Mooers AO (2012) The global diversity of birds in space and time. Nature 491: 444-448.

11. DaLage J-L, Kergoat GJ, Maczkowiak F, Silvain J-F, Cariou M-L, et al. (2007) A phylogeny of Drosophilidae using the *Amyrel* gene: questioning the *Drosophila melanogaster* species group boundaries J Zool Syst Evol Res 45: 47-63.

12. Tamura K, Battistuzzi FU, Billing-Ross P, Murillo O, Filipski A, et al. (2012) Estimating divergence times in large molecular phylogenies. Proc Natl Acad Sci U S A 109: 19333-19338.

13. Obbard DJ, Maclennan J, Kim KW, Rambaut A, O'Grady PM, et al. (2012) Estimating divergence dates and substitution rates in the *Drosophila* phylogeny. Mol Biol Evol 29: 3459-3473.

14. Oliveira DC, Almeida FC, O'Grady PM, Armella MA, DeSalle R, et al. (2012) Monophyly, divergence times, and evolution of host plant use inferred from a revised phylogeny of the *Drosophila repleta* species group. Mol Phylogenet Evol 64: 533-544.

15. Clayton FE (1998) Published karyotypes of the Drosophilidae. DIS 81: 5-125.

16. Pavan C, da Cunha AB (1947) Espécies brasileiras de *Drosophila*. Bol Fac Filos, Cienc e Letras Univ S Paulo, Biologia Geral 86: 3-46.

17. Wharton L. Interspecific hybridization in the *melanopalpa* subgroup of the *repleta* complex; 1946. GSA. pp. 235-235.

18. Vibranovski MD, Koerich LB, Carvalho AB (2008) Two new Y-linked genes in *Drosophila melanogaster*. Genetics 179: 2325-2327.

19. Chen ZX, Sturgill D, Qu J, Jiang H, Park S, et al. (2014) Comparative validation of the *D. melanogaster* modENCODE transcriptome annotation. Genome Res 24: 1209-1223.

20. Richards S, Liu Y, Bettencourt BR, Hradecky P, Letovsky S, et al. (2005) Comparative genome sequencing of *Drosophila pseudoobscura*: chromosomal, gene, and cis-element evolution. Genome Res 15: 1-18.

21. Rose TM, Henikoff JG, Henikoff S (2003) CODEHOP (COnsensus-DEgenerate hybrid oligonucleotide primer) PCR primer design. Nucleic Acids Research 31: 3763-3766.

22. International Glossina Genome I (2014) Genome sequence of the tsetse fly (*Glossina morsitans*): vector of African trypanosomiasis. Science 344: 380-386.

23. Holt RA, Subramanian GM, Halpern A, Sutton GG, Charlab R, et al. (2002) The genome sequence of the malaria mosquito *Anopheles gambiae*. Science 298: 129-+.

24. Nene V, Wortman JR, Lawson D, Haas B, Kodira C, et al. (2007) Genome sequence of *Aedes aegypti*, a major Arbovirus vector. Science 316: 1718-1723.

25. Reugels AM, Kurek R, Lammermann U, Bunemann H (2000) Mega-introns in the dynein gene *DhDhc7(Y)* on the heterochromatic Y chromosome give rise to the giant *Threads* loops in primary spermatocytes of *Drosophila hydei*. Genetics 154: 759-769.

26. Compton T (1990) Degenerate primers for DNA amplification. In: Innis MA, Gelfand DH, Sninsky JJ, White TJ, editors. PCR Protocols: A Guide to Methods and Applications. New York: Academic Press Inc. pp. 39-45.

27. Dieffenbach CW, Dveksler GS (1995) PCR primer : a laboratory manual. Plainview, N.Y.: Cold Spring Harbor Laboratory Press. xii, 714 p.

28. Diaz-Castillo C, Golic KG (2007) Evolution of gene sequence in response to chromosomal location. Genetics 177: 359-374.

29. Carvalho AB, Clark AG (2005) Y chromosome of *D. pseudoobscura* is not homologous to the ancestral *Drosophila* Y. Science 307: 108-110.

30. Larracuente AM, Noor MAF, Clark AG (2010) Translocation of Y-linked genes to the dot chromosome in *Drosophila pseudoobscura*. Mol Biol Evol 27: 1612.

31. Chang CH, Larracuente AM (2017) Genomic changes following the reversal of a Y chromosome to an autosome in *Drosophila pseudoobscura*. Evolution 71: 1285-1296.

32. Nozawa M, Onizuka K, Fujimi M, Ikeo K, Gojobori T (2016) Accelerated pseudogenization on the neo-X chromosome in *Drosophila miranda*. Nat Commun 7: 13659.

33. *Drosophila* 12 Genomes Consortium (2007) Evolution of genes and genomes on the *Drosophila* phylogeny. Nature 450: 203-218.

34. dos Santos G, Schroeder AJ, Goodman JL, Strelets VB, Crosby MA, et al. (2015) FlyBase: introduction of the *Drosophila melanogaster* Release 6 reference genome assembly and large-scale migration of genome annotations. Nucleic Acids Res 43: D690-697.

35. Gatti M, Pimpinelli S (1983) Cytological and genetic analysis of the Y-chromosome of *Drosophila melanogaster* .1. Organization of the fertility factors. Chromosoma 88: 349-373.

36. Kennison JA (1981) The genetic and cytological organization of the Y-chromosome of *Drosophila melanogaster*. Genetics 98: 529-548.

37. Carvalho AB, Lazzaro BP, Clark AG (2000) Y chromosomal fertility factors *kl-2* and *kl-3* of *Drosophila melanogaster* encode dynein heavy chain polypeptides. Proc Natl Acad Sci U S A 97: 13239-13244.

38. Carvalho AB, Dobo BA, Vibranovski MD, Clark AG (2001) Identification of five new genes on the Y chromosome of *Drosophila melanogaster*. Proc Natl Acad Sci U S A 98: 13225-13230.

39. Gepner J, Hays TS (1993) A fertility region on the Y-chromosome of *Drosophila melanogaster* encodes a dynein microtubule motor. Proc Natl Acad Sci U S A 90: 11132-11136.

40. Izumitani HF, Kusaka Y, Koshikawa S, Toda MJ, Katoh T (2016) Phylogeography of the subgenus *Drosophila* (Diptera: Drosophilidae): evolutionary history of faunal divergence between the Old and the New Worlds. PLoS One 11: e0160051.

41. van der Linde K, Houle D, Spicer GS, Steppan SJ (2010) A supermatrix-based molecular phylogeny of the family Drosophilidae. Genet Res (Camb) 92: 25-38.

42. Yassin A, Da Lage JL, David JR, Kondo M, Madi-Ravazzi L, et al. (2010) Polyphyly of the Zaprionus genus group (Diptera: Drosophilidae). Mol Phylogenet Evol 55: 335-339.

43. Russo CAM, Takezaki N, Nei M (1995) Molecular phylogeny and divergence times of drosophilid species. Mol Biol Evol 12: 391-404.

44. Yassin A (2013) Phylogenetic classification of the Drosophilidae Rondani (Diptera): the role of morphology in the postgenomic era. Systematic Entomology 38: 349-364.

45. Throckmorton LH (1975) The phylogeny, ecology and geography of *Drosophila*. In: King RC, editor. Handbook of Genetics. New York: Plenum Publishing Corporation. pp. 421–469.

46. Hatadani LM, McInerney JO, de Medeiros HF, Junqueira AC, de Azeredo-Espin AM, et al. (2009) Molecular phylogeny of the *Drosophila tripunctata* and closely related species groups (Diptera: Drosophilidae). Mol Phylogenet Evol 51: 595-600.

47. Robe LJ, Valente VL, Loreto EL (2010) Phylogenetic relationships and macro-evolutionary patterns within the *Drosophila tripunctata* "radiation" (Diptera: Drosophilidae). Genetica 138: 725-735.

48. Yassin A, Araripe LO, Capy P, Da Lage JL, Klaczko LB, et al. (2008) Grafting the molecular phylogenetic tree with morphological branches to reconstruct the evolutionary history of the genus *Zaprionus* (Diptera: Drosophilidae). Mol Phylogenet Evol 47: 903-915.

49. Grimaldi DA (1990) A phylogenetic, revised classification of genera in the Drosophilidae (Diptera). Bull Am Mus Nat Hist: 1-139.

50. Baimai V (1980) Metaphase karyotypes of certain species of the *Drosophila montium* subgroup. Japan J Genet 55: 165-175.

51. Deng Q, Zeng Q, Qian Y, Li C, Yang Y (2007) Research on the karyotype and evolution of *Drosophila melanogaster* species group. J Genet Genomics 34: 196-213.

52. Shyamala BV, Ranganath HA (1989) Metaphase karyotype differentiation in 8 species of the *montium* subgroup of *Drosophila*. Genetica 79: 191-196.

53. Sturtevant HA, Novitski E (1941) The homologies of the chromosome elements in the genus *Drosophila*. Genetics 26: 517-541.

54. Rao PM, Ranganath HA (1991) Karyotype differentiation among members of the *immigrans* species group of *Drosophila*. Genetica 83: 145-152.

55. Ward BL, Heed WB (1970) Chromosome phylogeny of *Drosophila pachea* and related species. J Hered 61: 248-258.

56. Muller HJ (1940) Bearings of the *Drosophila* work on systematics. In: Huxley J, editor. New Systematics. Oxford: Clarendon Press. pp. 185–268.

57. Vieira CP, Almeida A, Dias JD, Vieira J (2006) On the location of the gene(s) harbouring the advantageous variant that maintains the X/4 fusion of *Drosophila americana*. Genet Res 87: 163-174.

58. Flores SV, Evans AL, McAllister BF (2008) Independent origins of new sex-linked chromosomes in the *melanica* and *robusta* species groups of *Drosophila*. BMC Evol Biol 8: 33.

59. Allen SL, Delaney EK, Kopp A, Chenoweth SF (2017) Single-molecule sequencing of the *Drosophila serrata* genome. G3 7: 781-788.

60. Birney E, Durbin R (2000) Using GeneWise in the *Drosophila* annotation experiment. Genome Res 10: 547-548.
